# Persistent Vomiting Among Children With Acute Gastroenteritis: A Secondary Analysis of a Randomized Clinical Trial

**DOI:** 10.1001/jamanetworkopen.2026.10898

**Published:** 2026-05-06

**Authors:** Madeleine Sumner, Jianling Xie, Sarah Williamson-Urquhart, Amy C. Plint, Andrew Dixon, Darcy Beer, Gary Joubert, Yaron Finkelstein, Petros Pechlivanoglou, Terry Klassen, Stephen B. Freedman, on behalf of Pediatric Emergency Research Canada (PERC)

**Affiliations:** 1 Department of Pediatrics, Cumming School of Medicine, University of Calgary, Calgary, Alberta, Canada; 2 Section of Pediatric Emergency Medicine, Department of Pediatrics, Cumming School of Medicine, University of Calgary, Calgary, Alberta, Canada; 3 Section of Emergency Medicine, Department of Pediatrics, Alberta Children’s Hospital and Cumming School of Medicine, University of Calgary, Calgary, Alberta, Canada; 4 Department of Pediatrics, Children’s Hospital of Eastern Ontario, University of Ottawa, Ottawa, Ontario, Canada; 5 Children’s Hospital of Eastern Ontario Research Institute, Ottawa, Ontario, Canada; 6 Department of Emergency Medicine, University of Ottawa, Ottawa, Ontario, Canada; 7 Department of Pediatrics, Stollery Children’s Hospital, Edmonton, Alberta, Canada; 8 Faculty of Medicine and Dentistry, Women and Children’s Health Research Institute, University of Alberta, Edmonton, Alberta, Canada; 9 Max Rady College of Medicine, Pediatrics and Child Health, Rady Faculty of Health Sciences, University of Manitoba, Winnipeg, Manitoba, Canada; 10 Children’s Hospital Research Institute of Manitoba, Winnipeg, Manitoba, Canada; 11 Division of Emergency Medicine, London Health Sciences Centre, London, Ontario, Canada; 12 Department of Paediatrics, Schulich School of Medicine and Dentistry, Western University, London, Ontario, Canada; 13 Division of Emergency Medicine, Department of Paediatrics, Hospital for Sick Children, University of Toronto, Toronto, Ontario, Canada; 14 Division of Clinical Pharmacology and Toxicology, Department of Paediatrics, Hospital for Sick Children, University of Toronto, Toronto, Ontario, Canada; 15 Child Health Evaluative Sciences, SickKids Research Institute, Toronto, Ontario, Canada; 16 Institute of Health Policy Management and Evaluation, University of Toronto, Toronto, Ontario, Canada; 17 Department of Pediatrics, College of Medicine, University of Saskatchewan, Saskatoon, Saskatchewan, Canada; 18 Jim Pattison Children’s Hospital, Saskatoon, Saskatchewan, Canada; 19 Sections of Pediatric Emergency Medicine and Gastroenterology, Department of Pediatrics, Cumming School of Medicine, University of Calgary, Calgary, Alberta, Canada; 20 Department of Emergency Medicine, Cumming School of Medicine, University of Calgary, Calgary, Alberta, Canada

## Abstract

**Importance:**

Children with acute gastroenteritis–associated vomiting discharged from emergency departments (EDs) have improved outcomes when provided with ondansetron for home use. However, only one-third of children who present with significant vomiting experience ongoing vomiting after discharge.

**Objective:**

To identify characteristics associated with 3 or more vomiting episodes among pediatric patients within 24 hours of ED discharge.

**Design, Setting, and Participants:**

This nonprespecified secondary analysis of a randomized clinical trial of children aged 6 months to less than 18 years presenting to EDs between September 14, 2019, and June 27, 2024, with acute gastroenteritis–associated vomiting and who were followed up for 7 days. A prognostic score was derived using generalized linear mixed models across 10 imputed datasets. Data analysis was performed between May 9, 2025, and February 13, 2026.

**Main Outcomes and Measures:**

The primary outcome was 3 or more episodes of vomiting within 24 hours of ED discharge. Secondary outcomes included unscheduled health care revisits, intravenous fluid administration, and hospitalization within 7 days after the ED visit.

**Results:**

Of 1030 children enrolled, 977 had follow-up data available and were included in this analysis (median age, 47.0 months [IQR, 22.1-80.1 months]; 493 [50.5%] girls; 925 [89.8%] with complete follow-up data). Eighty of 927 children (8.6%) had 3 or more episodes of vomiting in the 24 hours after ED discharge. In unadjusted analysis, only age 6 months to less than 2 years was associated with ongoing vomiting after discharge (odds ratio [OR], 2.17; 95% CI, 1.37-3.43). In multivariable regression analysis, variables associated with postdischarge vomiting included age 6 months to less than 2 years, symptom duration of 24 to 48 hours, or 10 or more vomiting episodes in the 24 hours preceding the ED visit. In a predictive model, a score of 4 points or more was associated with a 13.6% (95% CI, 9.9%-18.1%) probability of 3 or more vomiting episodes within 24 hours of ED discharge, with a sensitivity of 0.50 (95% CI, 0.39-0.61) and specificity of 0.70 (95% CI, 0.67-0.73). Children with 3 or more vomiting episodes within 24 hours of discharge, compared with those without, were more likely to have an unscheduled health care visit (33 of 80 [41.3%] vs 65 of 846 [7.7%]; difference, 33.6%; 95% CI, 22.6%-44.5%), receive intravenous fluids (9 of 80 [11.3%] vs 15 of 846 [1.8%]; difference, 9.5%; 95% CI, 2.5%-16.5%), and be hospitalized (5 of 80 [6.2%] vs 9 of 846 [1.1%]; difference, 5.2%; 95% CI, −0.2% to 10.5%) within 7 days of discharge.

**Conclusions and Relevance:**

In this analysis of children presenting for ED care with vomiting, younger children and those unwell for 24 to 48 hours with 10 or more episodes of vomiting at presentation were more likely to have persistent vomiting after discharge. Based on these findings, these children are most likely to benefit from being provided ondansetron for home administration.

**Trial Registration:**

ClinicalTrials.gov Identifier: NCT03851835

## Key Points

**Question:**

Among children discharged from the emergency department (ED) with vomiting due to acute gastroenteritis, which patient characteristics are associated with persistent vomiting?

**Findings:**

In this secondary analysis of a randomized clinical trial, among 977 children, those aged 6 months to less than 2 years, those with a short duration of vomiting and/or diarrhea, and those with 10 episodes or more of vomiting during the 24 hours preceding ED presentation were statistically significantly more likely to have 3 or more episodes of postdischarge vomiting in a 24-hour period, as well as greater health care utilization.

**Meaning:**

These findings suggest that young children and those early in their illness with significant vomiting are most likely to experience ongoing vomiting after ED discharge and to utilize health care resources.

## Teaser

This secondary analysis of a randomized clinical trial examines independent risk factors associated with ongoing vomiting among pediatric patients within 24 hours of ED discharge.

## Introduction

Acute gastroenteritis accounts for over 10 million emergency department (ED) visits and 1 million hospitalizations in the US each year.^1^ Although most children with acute gastroenteritis have mild illness, up to 20% of those presenting to the ED experience moderate to severe disease, 15% return within 7 days, and 2% require hospitalization.^2,3^ The ability to reduce symptoms and ongoing care needs after discharge could mitigate the economic and clinical impacts of illness.

Although numerous factors are associated with ED revisits, including younger age,^2,3^ intravenous fluid administration,^2,3^ increased number of vomiting episodes^2,3^ and days of diarrhea,^3^ and absence of a primary care physician,^4^ vomiting frequency is most strongly associated with adverse outcomes. Children vomiting more than 5 times within the 24 hours preceding ED presentation are 3 times as likely to have a moderate to severe disease course compared with those with less vomiting.^2^ Single-dose ondansetron therapy in the ED reduces vomiting and decreases intravenous fluid administration,^5^ and as-needed ondansetron during the 48 hours after ED discharge further reduces disease severity.^6^ Given that 70% of children with significant vomiting at ED presentation have no further vomiting after discharge,^6^ the ability to identify those most likely to have ongoing vomiting would enable judicious use of postdischarge ondansetron.

To help inform optimal ondansetron dispensing after an ED visit, we conducted a secondary analysis of an existing dataset to (1) identify independent risk factors associated with ongoing vomiting during the 24 hours after ED discharge; (2) derive and validate a score to identify which patients are most likely to experience ongoing vomiting after ED discharge; and (3) evaluate the ability of the score to identify children most likely to experience unscheduled health care visits and receive intravenous fluid administration after ED discharge.

## Methods

### Study Design

We conducted a secondary analysis of a multicenter randomized clinical trial in which children with frequent vomiting seeking ED care between September 14, 2019, and June 27, 2024, were randomized to receive ondansetron or matching placebo every 8 hours, as needed, for ongoing vomiting (trial protocol in Supplement 1).^7^ Participants were followed up for 7 days to collect outcomes. For each participant, written informed consent was obtained from the participant’s caregiver, from the participant, or from both, along with assent, as appropriate according to participant age and institutional requirements. Research ethics board approval was obtained at all sites. This secondary analysis, which follows the Transparent Reporting of a Multivariable Prediction Model for Individual Prognosis or Diagnosis (TRIPOD) reporting guidelines,^8^ was not prespecified in the original trial protocol. This analysis was approved by the Conjoint Health Research Ethics Board of the University of Calgary.

### Participants

Eligible children were aged 6 months to less than 18 years, had an acute intestinal infection, and were administered ondansetron during their ED visit. They had 3 or more vomiting episodes in the preceding 24 hours, symptoms for less than 72 hours, and 1 or more vomiting episode within 6 hours. Children were excluded if they had bilious or bloody vomitus or allergy to ondansetron or any investigational medication components. They were also ineligible if they had a past medical history of significant cardiac disease or glucose-6 phosphate dehydrogenase deficiency or took any QTc-prolonging medications.^7^ In this analysis we also excluded children who were admitted at the index ED visit. Participation was limited to 1 gastroenteritis episode per child.

### Outcomes

Our primary outcome was 3 or more episodes of vomiting within 24 hours of ED discharge. Secondary outcomes included unscheduled health care visits, intravenous fluid administration, and hospitalization within 7 days after the index ED visit. Unscheduled health care visits were defined as presentation for assessment and/or care in a primary care clinic, walk-in clinic, or ED related to vomiting, diarrhea, dehydration, fever, abdominal pain, or fluid refusal; medical appointments that were set up prior to illness onset or those booked or recommended at the time of ED discharge were excluded.

### Data Collection

Baseline data were collected at the index visit by trained research nurses. Telephone or email-based electronic surveys (based on parental preference) were administered 24 and 48 hours after enrollment. On day 7, caregivers provided summary data covering the interval from 48 hours after enrollment through day 7. Medical record review was performed to verify data related to ED revisits, intravenous fluid administration, and hospitalizations.

### Statistical Analysis

Statistical analysis was performed between May 9, 2025, and February 13, 2026. Baseline characteristics were summarized; continuous variables were reported with medians and interquartile ranges (IQRs) and categorical variables as counts and percentages. Candidate variables were selected a priori based on clinical relevance; these included patient characteristics (sex, age^9^), illness severity at presentation (duration of symptoms,^10^ number of vomiting^10,11^ and diarrheal episodes in the 24 hours prior to the ED visit,^10,12^ presence of fever^11^), and treatments that may modify postdischarge symptoms (receipt of intravenous fluids^13^ and ondansetron at the ED visit^14^). Multiple imputation using chained equations was performed to address missing data in the regression analyses; other analyses were conducted without missing value imputation.^15^ The imputation model included all candidate variables, the primary outcome, and study site to preserve associations between covariates and outcome. To avoid inducing deterministic relationships, only missing components were imputed; these were then used to calculate derived variables, as required. Ten imputed datasets were generated.^16^ Ondansetron administration was defined as positive if a participant randomized to ondansetron reported administering the medication within 24 hours of ED discharge or a participant in the placebo arm reported taking ondansetron outside of the study protocol (ie, crossover).

We assessed the unadjusted association between prespecified variables and the primary outcome using generalized linear mixed models (GLMM) with a binomial distribution and logit link, incorporating a random intercept for study site to account for clustering. Each covariate was modeled individually; odds ratios (ORs) were pooled across the 10 imputed datasets.^17^ As additional sensitivity analyses, we compared effect estimates from (1) fully specified complete-case mixed-effects logistic regression models including all candidate variables, (2) reduced complete-case mixed-effects models including variables retained after LASSO selection, (3) fully specified and reduced mixed-effects models using multiply imputed data, and (4) a fully specified linear probability model with Eicker-Huber-White heteroskedasticity-robust standard errors. To quantify the impact of missing data on estimation precision, the fraction of missing information was calculated for each pooled regression coefficient from multiply imputed models. These analyses were conducted to evaluate robustness of regression estimates to model specification and missing data handling. Multicollinearity was assessed using generalized variance inflation factors; no evidence of problematic collinearity (variance inflation factors >5) was observed.

To develop a clinical score for the outcome of 3 or more vomiting episodes within 24 hours of discharge, we applied GLMM with least absolute shrinkage and selection operator (GLMM-LASSO) on each of the imputed datasets.^18^ Each model included a random intercept for study site to account for within-site correlation. Ondansetron administration group (yes vs no) was included as a fixed effect covariate in the model to adjust for the known randomized treatment effect on vomiting outcomes,^6^ allowing unbiased estimation of effects independent of treatment assignment. Additional details of model derivation are provided in the eMethods in Supplement 2.

We evaluated score performance, using the original, nonimputed dataset, to identify children most likely to experience 4 outcomes after ED discharge: (1) 3 or more episodes of vomiting within 24 hours, (2) unscheduled health care visits within 7 days, (3) intravenous fluid administration within 7 days, and (4) hospital admission within 7 days. For each outcome, we fitted a GLMM with the score as a continuous variable and a random intercept for study site. Discrimination was evaluated using the area under the receiver operating characteristic curve (AUC).^19^ To estimate the variability of the AUC, we generated 1000 bootstrap samples and calculated the mean AUC, standard deviation, standard error, and 95% CI. The optimal score threshold was determined using the Youden Index,^20^ with corresponding sensitivity and specificity reported. Model calibration was assessed by comparing predicted probabilities with observed outcome rates at each score level using calibration plots with 95% CIs. Model fit was evaluated using likelihood ratio tests comparing the full model (with the score) with a null model including only the site-level random intercept. The same evaluation process was applied independently to each outcome to assess the broader utility of the risk score.

All statistical tests were 2-sided, and *P* < .05 was considered statistically significant. Analyses were conducted using R, version 4.3.0 (R Project for Statistical Computing).

## Results

### Participants

A total of 2431 patients were assessed, and 1030 (42.4%) were eligible and consented to participate: 517 (50.2%) were allocated to ondansetron, 512 (49.8%) were allocated to placebo, and 1 participant was not randomized (Figure). Of the 1029 patients allocated, 977 (94.9%) had some follow-up data available, of whom 925 (94.7%) had complete data and 52 (5.3%) required multiple imputation for data completion. Median age was 47.0 months (IQR, 22.1-80.1 months), 493 of 977 were girls (50.5%), and 484 of 977 were boys (49.5%), and 18.7% (174 of 930) received a dose of ondansetron within 24 hours of discharge (Table 1).

**Figure.**
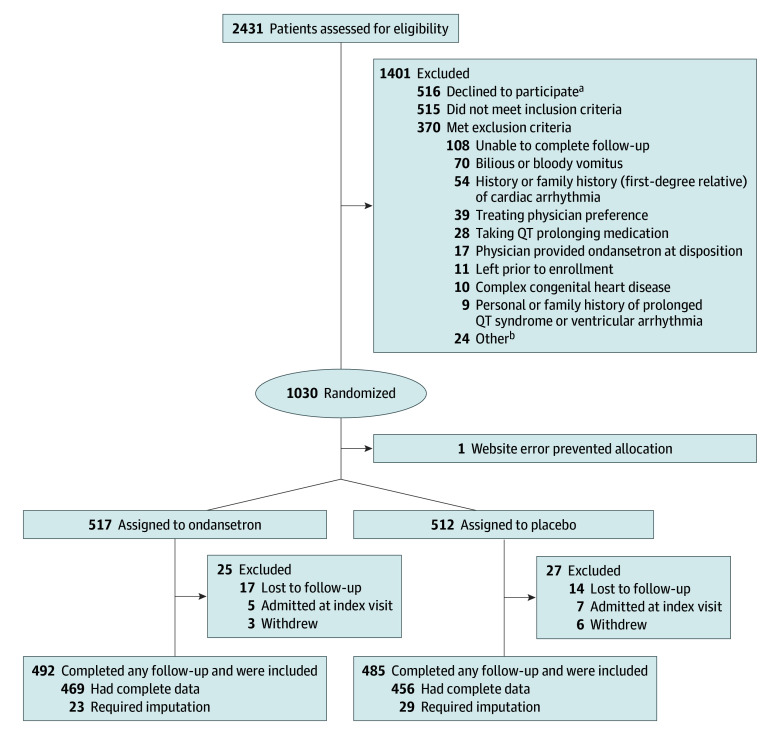
Participant Eligibility, Enrollment, and Study Completion ^a^Patients may have met more than 1 exclusion criterion. ^b^Other includes taking medication known to cause electrolyte abnormalities (n = 8), taking serotonergic or neuroleptic medication (n = 4), taking another 5-HT3 antagonist (n = 4), previously enrolled in this trial (n = 4), caregiver uncertain about exclusion criteria (n = 2), allergy to serotonin receptor antagonists or components of active or placebo elixir (n = 1), and glucose-6-phosphate dehydrogenase deficiency (n = 1).

**Table 1.** Baseline Characteristics of the Study Cohort Based on Occurrence of At Least 3 Episodes of Vomiting Within 24 Hours of ED Discharge^a^

Variable	Total cohort (N = 977)	≥3 Episodes of vomiting within 24 h after ED discharge^b^		
		No (n = 847)	Yes (n = 80)	*P* value
Sex, No. (%)				
Female	493 (50.5)	432 (51.0)	36 (45.0)	.36
Male	484 (49.5)	415 (49.0)	44 (55.0)	
Age group, No. (%)				
6 mo to <2 y	260 (26.6)	213 (25.1)	35 (43.8)	.005
2 y to <5 y	343 (35.1)	306 (36.1)	21 (26.2)	
5 y to <10 y	291 (29.8)	257 (30.3)	18 (22.5)	
≥10 y	83 (8.5)	71 (8.4)	6 (7.5)	
Baseline duration of symptoms, No. (%)				
<24 h	687 (70.3)	601 (71.0)	51 (63.7)	.35
24 h to <48 h	189 (19.3)	158 (18.7)	20 (25.0)	
48 h to 72 h	101 (10.3)	88 (10.4)	9 (11.2)	
Baseline vomiting episodes in 24 h prior to the ED visit, No. (%)				
3 to <5	171 (17.5)	152 (17.9)	12 (15.0)	.37
5 to <10	411 (42.1)	361 (42.6)	30 (37.5)	
≥10	395 (40.4)	334 (39.4)	38 (47.5)	
Baseline diarrhea episodes in 24 h prior to the ED visit, No./total No. (%)^c^				
0 to <5	874/976 (89.5)	764 (90.2)	71 (88.8)	.86
≥5	102/976 (10.5)	82 (9.7)	9 (11.2)	
Fever at baseline, No. (%)	338 (34.6)	297 (35.1)	26 (32.5)	.74
Intravenous fluids during index ED visit, No. (%)	70 (7.2)	58 (6.8)	6 (7.5)	>.99
Ondansetron administered within 24 h of ED discharge, No./total No. (%)^d^	174/930 (18.7)	145 (17.1)	29 (36.2)	<.001
Unscheduled health care visit within 7 d after ED discharge, No./total No. (%)^e^	109/974 (11.2)	65 (7.7)	33 (41.2)	<.001
Hospitalized within 7 d after ED discharge, No./total No. (%)^e^	14/974 (1.4)	9 (1.1)	5 (6.2)	.001
Received intravenous fluids within 7 d after ED discharge, No./total No. (%)	24/974 (2.5)	15 (1.8)	9 (11.3)	<.001

Abbreviation: ED, emergency department.

^a^ Total cohort includes all children included in the analyses (N = 977); in this table, data reported regarding occurrence of 3 or more episodes of vomiting within 24 hours after ED discharge includes only those with complete data (N = 927).

^b^ Fifty children with missing data on number of vomiting episodes within 24 hours of discharge, values imputed for calculation of the logistic model.

^c^ One child with missing data.

^d^ Forty-seven children with missing data.

^e^ Three children with missing data.

### Primary Outcome

In the 24 hours after the index ED visit, 80 of 927 children (8.6%) had 3 or more episodes of vomiting, of whom 35 (43.8%) were 6 months to less than 2 years old (Table 1). In unadjusted analysis, the only candidate variable associated with the primary outcome was age 6 months to less than 2 years (OR, 2.17; 95%CI: 1.37-3.43) (Table 2). LASSO regression identified the following variables for inclusion in the score: age 6 months to less than 2 years (6 points), duration of symptoms 24 to 48 hours (2 points), and 10 or more episodes of vomiting in the 24 hours preceding ED presentation (2 points) (Table 3). Age 5 years to less than 10 years had a very small association with the likelihood of vomiting (mean log OR, −0.076), and this variable did not meet the threshold for score assignment. In mixed-effect logistic regression, the scoring system identified children with 3 or more episodes of vomiting in the 24 hours after ED discharge (OR, 1.16; 95% CI, 1.08-1.25; *P* < .001) (eTable 1 in Supplement 2). The receiver-operating characteristic curve for the score had a mean AUC of 0.63 (95% CI, 0.56-0.69) using 1000 bootstrapped samples (eFigure 1 in Supplement 2). With the Youden Index, an optimal score threshold of 3 points (rounded to 4, because 3 points is not a possible total score) was identified for the outcome of 3 or more episodes of vomiting in the 24 hours after ED discharge, with a sensitivity of 0.50 (95% CI, 0.39-0.61) and specificity of 0.70 (95% CI, 0.67-0.73). A score of 4 or more had a positive predictive value of 13.6% (95% CI, 9.9%-18.1%) and negative predictive value of 93.7% (95% CI, 91.5%-95.4%) for 3 or more vomiting episodes within 24 hours and a positive predictive value of 15.6% (95% CI, 11.7%-20.1%) and negative predictive value of 90.8% (95% CI, 88.4%-92.9%) for an unscheduled health care revisit within 7 days after ED discharge (Table 4). Predicted and observed probabilities are depicted in eFigure 2 in Supplement 2. Additional modeling to assess the robustness of our results revealed that across all approaches (complete case, multiply imputed datasets, logistic and linear models), effect estimates were consistent in direction and magnitude (eTable 2 in Supplement 2).

**Table 2.** Association Between Individual Variables and the Likelihood of Having ≥3 Vomiting Episodes Within 24 Hours After the Index ED Visit in GLMM

Variable	Unadjusted OR (95% CI)^a^
Sex	
Female	0.79 (0.50-1.26)
Male	1.26 (0.79-2.01)
Age	
6 mo to <2 y	2.17 (1.37-3.43)
2 to <5 y	0.64 (0.38-1.07)
5 to <10 y	0.66 (0.38-1.14)
≥10 y	1.08 (0.50-2.36)
Baseline duration of symptoms	
<24 h	0.69 (0.43-1.12)
24 to <48 h	1.55 (0.91-2.62)
48 to 72 h	1.06 (0.51-2.20)
Baseline vomiting episodes in 24 h prior to the ED visit	
3 to <5	0.75 (0.40-1.43)
5 to <10	0.79 (0.50-1.26)
≥10	1.47 (0.94-2.30)
≥5 Baseline diarrhea episodes in 24 h prior to the ED visit	1.41 (0.71-2.78)
Fever at baseline	0.88 (0.54-1.43)
Intravenous fluids during index ED visit	1.14 (0.48-2.71)
Ondansetron administered within 24 h of ED discharge	2.51 (1.55-4.06)

Abbreviations: ED, emergency department; GLMM, generalized linear mixed models; OR, odds ratio.

^a^ Each variable was modeled separately in a univariate regression model. Models were run on 10 multiple-imputed datasets, and the ORs were pooled. Study site was included as a random intercept to account for clustering.

**Table 3.** Model Performance to Identify Children With ≥3 Vomiting Episodes in 24 Hours After ED Discharge

Variable	Mean log OR	Assigned points in pediatric ongoing vomiting score
Age 6 mo to <2 y	0.630	6
Age 5 to <10 y^a^	−0.076	0
Baseline duration of symptoms 24-48 h	0.160	2
≥10 Baseline vomiting episodes in 24 h prior to the ED visit	0.151	2
Ondansetron administered within 24 h of ED discharge^b^	0.901	0
Maximum score	NA	10
Optimal threshold^c^	NA	4

Abbreviations: ED, emergency department; NA, not applicable; OR, odds ratio.

^a^ Variable age 5 years to less than 10 years was selected by the model but assigned a score of 0 because its mean log OR (–0.076) was below the predefined threshold for score assignment (absolute value <0.10). This approach supports a parsimonious risk score by prioritizing variables with stronger associations.

^b^ Variable of ondansetron administration in the 24 hours after ED discharge was adjusted for in the model but excluded from the score system because this does not have clinical meaning for a predictive score applied at initial patient presentation.

^c^ Based on calculated Youden Index the optimal threshold of the total score was 3 points to classify individuals who had 3 or more vomiting episodes after ED discharge, with a sensitivity of 0.50 (95% CI, 0.39-0.61) and specificity of 0.70 (95% CI, 0.67-0.73). This was rounded to 4 points because 3 is not a possible total score.

**Table 4.** Pediatric Ongoing Vomiting Score and Likelihood of ≥3 Vomiting Episodes in 24 Hours After the ED Discharge

Pediatric ongoing vomiting score	Model-predicted probability of outcome, % (95% CI)	Observed occurrence of ≥3 vomiting episodes in 24 h after ED discharge, No./total No. (%) [95% CI]
0	5.4 (3.8-7.6)	16/334 (4.8) [3.0-7.6]
2	7.2 (5.6-9.2)	24/332 (7.2) [4.9-10.5]
4	9.4 (7.6-11.6)	5/51 (9.8) [4.3-21.0]
6	12.3 (9.6-15.6)	15/127 (11.8) [7.3-18.6]
8	15.9 (11.5-21.6)	16/116 (13.8) [8.7-21.2]
10	20.4 (13.4-29.6)	4/17 (23.5) [9.6-47.3]

Abbreviation: ED, emergency department.

### Secondary Outcomes

In the 7 days after the index ED visit, 109 of 974 children (11.2%) had an unscheduled health care revisit (Table 1), including 73 (7.5%) to the ED, 25 (2.6%) to a primary care physician, and 11 (1.1%) to a walk-in clinic. Twenty-four children (2.5%) received intravenous fluids, and 14 (1.4%) were hospitalized for ongoing symptoms within 7 days of the index ED visit. Children with 3 or more episodes of vomiting in the 24 hours after ED discharge, compared with those with fewer episodes, were more likely to have unscheduled health care visits (33 of 80 [41.3%] vs 65 of 846 [7.7%]; difference, 33.6%; 95% CI, 22.6%-44.5%), receive intravenous fluids (9 of 80 [11.3%] vs 15 of 846 [1.8%]; difference, 9.5%; 95% CI, 2.5%-16.5%), and be hospitalized (5 of 80 [6.2%] vs 9 of 846 [1.1%]; difference, 5.2%; 95% CI, −0.2% to 10.5%) during the 7 days after the index visit. Evaluating the performance of our proposed score for these secondary outcomes revealed AUC values of 0.57 (95% CI, 0.52-0.63) for unscheduled health care revisits, 0.52 (95% CI, 0.43-0.64) for intravenous fluid administration, and 0.57 (95% CI, 0.45-0.69) for hospitalization within 7 days of ED discharge (eFigure 3 in Supplement 2).

## Discussion

In this secondary analysis of data from a large multicenter randomized clinical trial among children presenting to the ED with clinically significant vomiting from acute gastroenteritis, postdischarge vomiting was uncommon but was associated with increased health care utilization. Only 8.6% of children, or 1 in 12, had 3 or more episodes of vomiting in the 24 hours after ED discharge, and more than 40% of these children were aged 6 months to less than 2 years. Those with 3 or more episodes of vomiting during the 24 hours after ED discharge were over 5 times more likely to return for an unscheduled health care revisit, receive intravenous fluids, and be hospitalized within 7 days of the index visit. These children were also twice as likely to receive 1 or more as-needed home ondansetron doses within 24 hours of discharge, compared with those with less vomiting.

Understanding who may continue to vomit after ED discharge, and thus who may benefit from additional doses of ondansetron, is highly clinically relevant. Most children who present with vomiting to the ED are treated with a dose of oral ondansetron and subsequently experience a cessation of vomiting.^21^ In a study of children younger than 4 years with diarrhea-associated acute gastroenteritis, although 76% of children had vomiting at the time of the index ED visit, the median number of vomiting episodes in the 24 hours after the ED visit was zero.^22^ In a clinical trial that failed to demonstrate clinical benefit from administering post-ED discharge dimenhydrinate to children with acute gastroenteritis-associated vomiting, only 29% of children in the placebo group experienced 2 or more vomiting episodes in the 24 hours after ED discharge.^23^ The findings in the clinical trial from which our data were derived were consistent with the aforementioned studies: 67% of participants in the placebo group experienced no additional vomiting after ED discharge.^6^

Although home ondansetron administration after ED discharge leads to a lower risk of moderate to severe gastroenteritis during the subsequent 7 days than the provision of placebo, the number needed to treat is 15.^6^ Although ondansetron is generally a safe medication, in a clinical trial, children who received 3 or more doses of ondansetron after ED discharge experienced more frequent diarrhea.^6^ Although more than 90% of children eligible for our study experienced fewer than 3 episodes of vomiting after ED discharge, ondansetron ED discharge prescriptions have become ubiquitous in the US.^24,25^ Thus, to minimize adverse events, optimize benefit, and constrain costs, there is the need to promote clinical stewardship, as suggested by Zhao et al,^26^ by identifying children most likely to experience significant vomiting.

We identified 3 characteristics of children who experienced significant vomiting after discharge: age 6 months to less than 2 years, duration of symptoms of 24 to 48 hours at the time of index ED visit presentation, and 10 or more episodes of vomiting in the 24 hours preceding the ED visit. These factors were combined to derive a score with the optimal cut-point being 4 or more points. To exceed this score cut-point, children had to either be aged 6 months to less than 2 years or have both duration of symptoms of 24 to 48 hours and 10 or more episodes of vomiting in the 24 hours preceding ED presentation. Children with scores above this cut-point had a 13.6% probability of ongoing vomiting and a 15.6% probability of unscheduled health care revisit within 7 days, about twice the likelihood of children with scores less than 4. Based on practice preference, the score could assist in identifying those most likely to benefit from home ondansetron to enhance precision pediatric acute care therapeutics.

This secondary analysis identified children most likely to benefit from home ondansetron administration, as only 8% of children provided ondansetron were administered 3 or more doses after ED discharge. We found that children younger than 2 years of age are most likely to experience ongoing vomiting after ED discharge. This finding aligns with previous reports that have found younger age to be associated with more severe and prolonged episodes of gastroenteritis^2,27^ and ED revisits.^3,28^ Possible explanations include the high rates of norovirus infection among children with vomiting seeking ED care,^29^ with over 55% of all childhood norovirus infections occurring in children younger than 2 years of age.^30^ Although our model included multiple risk factors, it was dominated by age; thus, we support the targeted provision of as-needed home ondansetron, in addition to an ED-administered dose, to children 6 months to less than 2 years old presenting with significant and recent vomiting from acute gastroenteritis. We believe such an approach can reduce the likelihood of experiencing ongoing vomiting and moderate to severe gastroenteritis after ED discharge.

Other children who should be considered for home ondansetron doses to reduce the likelihood of ongoing vomiting include those early in the course of illness (day ≤2) and those with severe vomiting (eg, >10 episodes in the preceding 24 hours). These factors have been previously associated with increased ED revisits.^3^

### Limitations

This study has some limitations. Our target population was children who are well enough to be discharged from the ED; thus, we did not include laboratory or microbiologic covariates in our models, as most children with acute gastroenteritis who are discharged do not have such testing performed.^31^ Our model did not account for the presence of medical comorbidities; thus, our results apply primarily to children without significant underlying diseases. Given this was a secondary analysis of a double-blinded randomized clinical trial, all children in our cohort were sent home with an intervention medication (ie, ondansetron or placebo). It is possible there were inaccuracies in parent-reported outcome data that could be influenced by parental recall bias, anxiety, and what is considered as an episode of “vomiting.” We used multiple imputation to address missing variables in our model under the assumption of missing-at-random; however, residual bias due to departures from missing-at-random cannot be excluded. Last, we did not account for race and ethnicity in our model, although we acknowledge this has been shown to be associated with acute gastroenteritis severity.^27^

## Conclusions

In this analysis of children with recent and frequent vomiting who received ondansetron as part of clinical care in the ED, less than 10% experienced persistent significant vomiting after ED discharge. The characteristic most strongly associated with ongoing vomiting in the 24 hours after discharge was age, with vomiting most frequently occurring among those younger than 2 years. Based on these findings, in conjunction with the demonstrated efficacy of home-administered ondansetron to improve outcomes among children in our study population, our data support the targeted provision of ondansetron to be taken at home, as needed, for children aged 6 months to less than 2 years who present for ED care with significant and recent vomiting. In addition, children on day 2 of illness or less with more than 10 episodes of vomiting in the 24 hours prior to ED presentation were also likely to benefit from additional doses of ondansetron for home.

## Notes

Supplement 1.
Trial Protocol and Statistical Analysis Plan

Supplement 2. **eAppendix.** Model Derivation **eFigure 1.** Receiver Operating Characteristic Curve of the Derived Score to Correctly Classify the Occurrence of ≥3 Episodes of Vomiting 24 hours Following ED Discharge **eFigure 2.** Predicted and Observed Probabilities of (A) ≥3 Vomiting Episodes During the 24-Hours Following the Index Emergency Department Visit and (B) Unscheduled Health Care Visit in 7 Days After Index Emergency Department Discharge **eFigure 3.** Receiver Operating Characteristic Curve of the Derived Score to Correctly Classify the Occurrence of (A) Unscheduled Healthcare Revisit, (B) Intravenous Fluid Administration, and (C) Hospitalization Within 7-Days of Index ED Discharge **eTable 1.** Results of Mixed-Effect Logistic Regression Model for Each Outcome, With the Derived Score as the Independent Variable **eTable 2.** Effect Estimates for Occurrence of ≥3 Episodes of Vomiting in the 24-Hours Following Emergency Department Discharge Evaluated Using Complete Case and Multiple Imputation Datasets Analyzed Using Mixed-Effects Logistic Regression, Linear Probability Models With Robust Standard Errors, and LASSO-Selected Models
eReferences.

Supplement 3.
Members of Pediatric Emergency Research Canada

Supplement 4.
Data Sharing Statement
