# Supplementary material for: Persistent Vomiting Among Children With Acute Gastroenteritis: A Secondary Analysis of a Randomized Clinical Trial
Source: JAMA Netw Open. 2026 May 6;9(5):e2610898. doi: 10.1001/jamanetworkopen.2026.10898 (PMC13150644; doi:10.1001/jamanetworkopen.2026.10898)
Supplement: Supplement 1. — Trial Protocol and Statistical Analysis Plan [file jamanetwopen-e2610898-s001.pdf]

MULTI-DOSE ORAL ONDANSETRON FOR PEDIATRIC GASTROENTERITIS: A PRAGMATIC RANDOMIZED CONTROLLED TRIAL

Protocol Acronym or Number: OND18 – 2045

THE DOSE-AGE STUDY: Multi-**DOSE** Oral Ondansetron for Pediatric **A**cute **G**astro**E**nteritis

Clinicaltrials.gov Identified Number: NCT 03851835

Principal Investigator: Dr. Stephen Freedman

Clinical Trial Sponsor: The University of Calgary

Funded by: The Canadian Institutes of Health Research (CIHR)

Version Number: 4.0

Version Date: 25 November 2022

Summary of Changes from the Previous Version:

**Affected Section(s)**	**Summary of Revisions Made**	**Rationale**
Title page & all page headers	Previous approved version: 2020 March 02<br>Current version date: 2022 November 2022<br>Addition of a version number: 4.0	To reflect changes to document and more accurately track protocol version.<br>V1.0 – 03 February 2019<br>V2.0 – 14 March 2020<br>V3.0 – 02 March 2020<br>V4.0 – 25 November 2022
Various	Grammatical and typographical	Fix any grammatical and typographical issues
6.1.1<br>6.2.1<br>6.2.2<br>6.2.4	Minor changes to include both Zofran® and the generic ondansetron oral solution as options for the interventional product.	Due to intermittent availability of the brand name interventional product, Zofran®, the protocol will now allow for the use of both generic oral ondansetron and Novartis brand Zofran®. Use is based on availability of the primary product, Zofran®. The generic

	Addition of a second placebo formulation to match the smell of the generic ondansetron product.	ondansetron oral solution can be used if Zofran® is not available.
10.4	Update to Protocol Change Amendment Table	Summary of changes made to the study protocol: Inclusion of the generic oral ondansetron product and matching placebo formulation.
12	Inclusion of additional appendices. Now included are Appendices A, B, C, and D.	Inclusion of JAMP Pharmaceuticals generic Ondansetron Oral Solution Product Monograph.<br>Inclusion of the generic ondansetron oral solution matching placebo formulation.

Table of Contents

STATEMENT OF COMPLIANCE 1
1 PROTOCOL SUMMARY 1
1.1 Synopsis 1
1.2 Schema 2
1.3 Schedule of Activities (SoA) 4
2 INTRODUCTION 4
2.1 Study Rationale 4
2.2 Background 5
2.3 Risk/Benefit Assessment 7
2.3.1 Known Potential Risks 7
2.3.2 Known Potential Benefits 8
2.3.3 Assessment of Potential Risks and Benefits 9
3 OBJECTIVES AND ENDPOINTS 9
4 STUDY DESIGN 12
4.1 Overall Design 12
4.2 Scientific Rationale for Study Design 13
4.3 Justification for Dose 13
4.4 End of Study Definition 13
5 STUDY POPULATION 14
5.1 Inclusion Criteria 14
5.2 Exclusion Criteria 14
5.3 Lifestyle Considerations 15
5.4 Screen Failures 15
5.5 Strategies for Recruitment and Retention 15
6 STUDY INTERVENTION 16
6.1 Study Intervention(s) Administration 16
6.1.1 Study Intervention Description 16
6.1.2 Dosing and Administration 16
6.2 Preparation/Handling/Storage/Accountability 17
6.2.1 Acquisition and accountability 17
6.2.2 Formulation, Appearance, Packaging, and Labeling 17
6.2.3 Product Storage and Stability 18
6.2.4 Preparation 19
6.3 Measures to Minimize Bias: Randomization and Blinding 19
6.4 Study Intervention Compliance 20
6.5 Concomitant Therapy 20
6.5.1 Rescue Medicine 21
7 STUDY INTERVENTION DISCONTINUATION AND PARTICIPANT DISCONTINUATION/WITHDRAWAL 21
7.1 Discontinuation of Study Intervention 21
7.2 Participant Discontinuation/Withdrawal from the Study 21
7.3 Lost to Follow-Up 22
8 STUDY ASSESSMENTS AND PROCEDURES 22
8.1 Efficacy Assessments 22
8.2 Safety and Other Assessments 23
8.3 Adverse Events and Serious Adverse Events 25

8.3.1 Definition of Adverse Events (AE) 25
8.3.2 Definition of Serious Adverse Events (SAE) 25
8.3.3 Classification of an Adverse Event 25
8.3.4 Time Period and Frequency for Event Assessment and Follow-Up 27
8.3.5 Adverse Event Reporting 27
8.3.6 Serious Adverse Event Reporting 28
8.3.7 Reporting Events to Participants 29
8.3.8 Events of Special Interest 29
8.3.9 Reporting of Pregnancy 29
8.4 Unanticipated Problems 30
8.4.1 Definition of Unanticipated Problems (UP) 30
8.4.2 Unanticipated Problem Reporting 30
8.4.3 Reporting Unanticipated Problems to Participants 31
9 STATISTICAL CONSIDERATIONS 31
9.1 Statistical Hypotheses 31
9.2 Sample Size Determination 31
9.3 Populations for Analyses 32
9.4 Statistical Analyses 32
9.4.1 General Approach 32
9.4.2 Analysis of the Primary Efficacy Endpoint(s) 33
9.4.3 Analysis of the Secondary Endpoint(s) 35
9.4.4 Safety Analyses 36
9.4.5 Baseline Descriptive Statistics 37
9.4.6 Planned Interim Analyses 37
9.4.7 Sub-Group Analyses 38
9.4.8 Tabulation of Individual participant Data 38
9.4.9 Exploratory Analyses 38
10 SUPPORTING DOCUMENTATION AND OPERATIONAL CONSIDERATIONS 38
10.1 Regulatory, Ethical, and Study Oversight Considerations 38
10.1.1 Informed Consent Process 38
10.1.2 Study Discontinuation and Closure 40
10.1.3 Confidentiality and Privacy 40
10.1.4 Future Use of Stored Specimens and Data 41
10.1.5 Key Roles and Study Governance 42
10.1.6 Safety Oversight 43
10.1.7 Clinical Monitoring 43
10.1.8 Quality Assurance and Quality Control 44
10.1.9 Data Handling and Record Keeping 45
10.1.10 Protocol Deviations 46
10.1.11 Publication and Data Sharing Policy 46
10.1.12 Conflict of Interest Policy 47
10.2 Additional Considerations 47
10.3 Abbreviations 48
10.4 Protocol Amendment History 49
11 References 50
12 Appendices 53

STATEMENT OF COMPLIANCE

The trial will be conducted in accordance with Good Clinical Practice (GCP) as described in Health Canada's section C.05.010/Division 5 of the Food and Drug Regulations, International Conference on Harmonisation-Good Clinical Practice (ICH-GCP E11 and ICH-GCP E6 R2), Tri-Council Policy Statement (TCPS2, 2018); applicable federal, provincial and local regulatory and legislative requirements and in compliance with the Terms and Conditions of the CIHR SPOR Award. The Principal Investigator will assure that no deviation from, or changes to the protocol will take place without prior documented authorization/approval from a duly constituted Research Ethics Board (REB), except where necessary to eliminate an immediate hazard(s) to the trial participants. All personnel involved in the conduct of this study have completed Human Subjects Protection and ICH-GCP Training.

The protocol, informed consent form(s), recruitment materials, and all participant materials will be submitted to the REB for review and approval. Approval of both the protocol and the consent form must be obtained before any participant is enrolled. Any amendment to the protocol will require review and approval by the REB before the changes are implemented to the study. All changes to the consent form will be REB approved and a determination will be made regarding whether a new consent needs to be obtained from active participants who provided consent using a previous approved version of the consent form.

A Health Canada Clinical Trial Application (CTA) for this study will not be submitted as per written waiver provided by a Health Canada representative during the pre-CTA consultation phase (*email correspondence dated 18 December 2017*).

1 PROTOCOL SUMMARY

1.1 SYNOPSIS

**Title:**	MULTI-DOSE ORAL ONDANSETRON FOR PEDIATRIC GASTROENTERITIS: A RANDOMIZED CONTROLLED TRIAL
**Sponsor**	The University of Calgary
**Lead Investigator**	Dr. Stephen Freedman
**Study Description :**	A phase III, double-blind, parallel-design, randomized, placebo controlled trial to compare multi-dose oral Ondansetron with placebo as treatment for vomiting secondary to acute gastroenteritis (AGE), after Emergency Department discharge.<br><br>The primary outcome will be the post-index visit Modified Vesikari Scale (MVS) score.
**Objectives:**	**Primary Objective:** To determine if in previously healthy children, who present to an ED with gastroenteritis associated vomiting, is the proportion who develop *moderate to severe disease* [Modified Vesikari Score (MVS) ≥ 9] following ED evaluation, significantly different in those who receive ondansetron at home compared to those who receive placebo.

	<u>**Secondary Objectives:**</u> In this group of patients, amongst those receiving active treatment versus placebo:<br>1. Is there a difference in the (a) *duration of vomiting,* (b) *frequency of vomiting*, or (c) *the proportion who experience vomiting* following ED disposition?<br>2. Is there a difference in the proportion who require an *unscheduled health care provider* visit?<br>3. Is there a difference in the proportion *who require intravenous rehydration?*<br>4. Is there a difference in the *caregiver satisfaction with the therapy provided?*
**Endpoints:**	**Primary Endpoint:** Post-index visit MVS score.<br>**Secondary Endpoints will include:**<br>1. Vomiting: Frequency, Duration, Proportion (i.e. yes/no)<br>2. Health care use: Emergency Department, Primary Care<br>3. Intravenous rehydration<br>4. Caregiver Satisfaction with Therapy Provided: Likert Scale
**Study Population:**	Children and youth, age 6 months to 17.99 years will be enrolled at six (6) Canadian Emergency Departments. The total number of participants recruited will be 1030.
**Phase:**	Phase III
**Description of Sites/Facilities Enrolling Participants:**	Participants will be enrolled at six (6) pediatric emergency departments across Canada: Alberta Children's Hospital (Calgary, AB), Centre Hospitalier Universitaire de Sainte Justine (Montréal, QC), Children's Hospital of Eastern Ontario (Ottawa, ON), London Health Sciences Centre – Children's Hospital (London, ON), Stollery Children's Hospital (Edmonton, AB), and the Health Sciences Centre – Children's Hospital (Winnipeg, MB)
**Description of Study Intervention:**	Children who are provided a minimum of one dose of ondansetron as part of their routine clinical care AND meet other eligibility criteria will be randomized to receive an at-home kit with six (6) doses *of* ***Ondansetron Hydrochloride Dihydrate Oral Solution (4mg/5mL solution; dosed at 0.15mg/kg to a maximum single dose of 8mg)*** or equivalent volume in a *Placebo Oral Solution* to be administered no sooner than 8 hours after the initial clinical dose was provided by the ED physician. Over the subsequent 48 hours, the study intervention will be administered at a rate of <u>1 dose every 8 hours (q8h) to a maximum of 3 doses a day (in a 24 hour period (TID)) at the caregiver's discretion</u>. Two (2) additional doses will be provided to the caregiver in case the child vomits a dose.
**Study Duration:**	Approximately 36 months of active recruiting will be required to meet the sample size of 1030.
**Participant Duration:**	Screening, consent, and randomization will take place at the initial ED (baseline) visit. The follow-up period will last 7 days with questionnaires issued 24 hours, 48 hours and 168 hours (7 days) after informed consent is received, by telephone or email.

1.2 SCHEMA

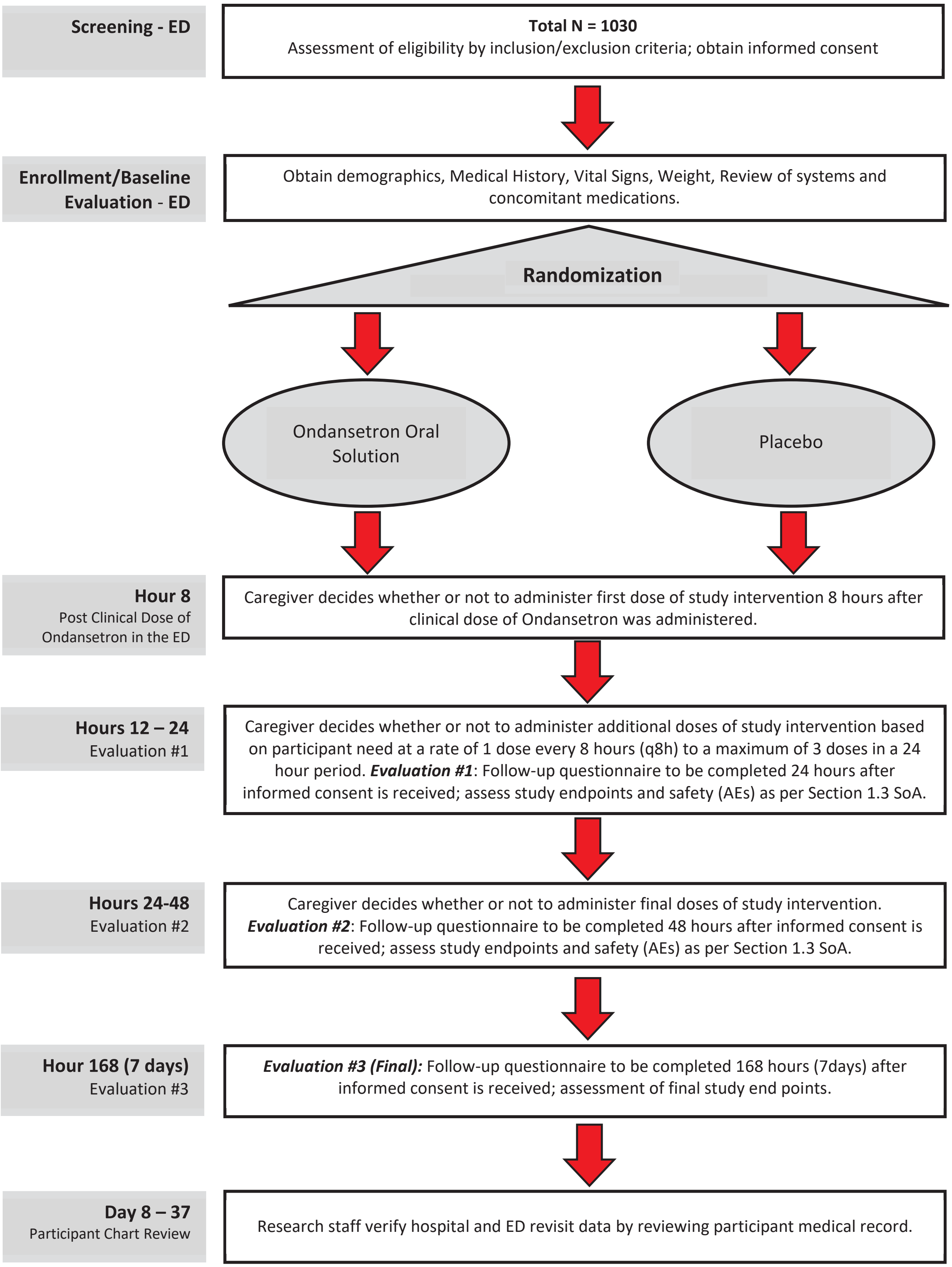
Screening - ED
Total N = 1030
Assessment of eligibility by inclusion/exclusion criteria; obtain informed consent
Enrollment/Baseline Evaluation - ED
Obtain demographics, Medical History, Vital Signs, Weight, Review of systems and concomitant medications.
Randomization
Ondansetron Oral Solution
Placebo
Hour 8
Post Clinical Dose of Ondansetron in the ED
Caregiver decides whether or not to administer first dose of study intervention 8 hours after clinical dose of Ondansetron was administered.
Hours 12 – 24
Evaluation #1
Caregiver decides whether or not to administer additional doses of study intervention based on participant need at a rate of 1 dose every 8 hours (q8h) to a maximum of 3 doses in a 24 hour period. Evaluation #1: Follow-up questionnaire to be completed 24 hours after informed consent is received; assess study endpoints and safety (AEs) as per Section 1.3 SoA.
Hours 24-48
Evaluation #2
Caregiver decides whether or not to administer final doses of study intervention. Evaluation #2: Follow-up questionnaire to be completed 48 hours after informed consent is received; assess study endpoints and safety (AEs) as per Section 1.3 SoA.
Hour 168 (7 days)
Evaluation #3
Evaluation #3 (Final): Follow-up questionnaire to be completed 168 hours (7days) after informed consent is received; assessment of final study end points.
Day 8 – 37
Participant Chart Review
Research staff verify hospital and ED revisit data by reviewing participant medical record.

1.3 SCHEDULE OF ACTIVITIES (SOA)

Below is a list of study activities identified by study time point. The study period will last 168 hours (7 days) from the Screening/Enrollment Visit to the Final Evaluation (*a 14 day and 30 day completion window is provided for follow-up questionnaires and collection of chart review data respectively*).

**Procedures**	**Presenting ED Visit Screening**	**Presenting ED Visit Enrollment/ Baseline Evaluation**	**Hour 8 Post Ondansetron Administration – ED**	**Hour 24* Evaluation #1**	**Hour 48* Evaluation #2**	**Hour 168 (Day 7)* + *14 days* Evaluation #3 (Final)**	**Hour 168 (Day 7) + *30 days* Chart Review**
Assessment of eligibility *Inclusion/Exclusion*	X						
Informed consent *Time 0*	X	X					
Demographics		X					
Medical and illness history		X					
Collect vital signs *(HR, RR, temperature)*		X					
Weight *(obtained from clinical chart)*		X					
Review of systems		X					
Concomitant medication review		X ----------	----------	----------	----------	----------X	
Randomization – *Enrollment Treatment Allocation*		X					
Administer study intervention			X[ab]	X[b]	X[b]		
Adverse Event (AE) review and evaluation		X ----------	----------	----------	----------	---------- X	
Follow-up questionnaire *email or telephone*				X	X	X	
Chart Review *Collect and confirm healthcare re-visit data*							X
Complete Case Report Forms (CRFs)	X	X		X	X	X	X

** Visits will only be considered out of window if the study team is unable to contact the caregiver for follow-up (24, 48, and 168 hours) within 21 days of randomization.*
[a] First dose of study intervention can be administered 8 hours after administration of clinical dose of Ondansetron (at presenting ED visit)
[b] Dose of study intervention will be administered at home based on caregiver determination. The final dose should be administered within 48 hours of first clinical dose of Ondansetron. Should the caregiver administer doses of study medication >72 hours post clinical dose of Ondansetron, it will be considered a protocol deviation. We will collect dose details at each follow-up time point. Any doses administered after the 48 hour follow-up time point will be recorded on the 168 hour (Day 7) follow-up questionnaire.

2 INTRODUCTION

2.1 STUDY RATIONALE

The annual burden of acute gastroenteritis in the United States includes 17 million related episodes and 473,832 hospitalizations.[1] Although oral-rehydration therapy is recommended for children with mild-to-moderate dehydration, it has historically been underused with emergency department (ED) clinicians being more likely to choose intravenous over oral rehydration especially when vomiting is a major symptom.[2] In fact, nearly 95% of children undergoing oral rehydration in Canadian EDs present with recent vomiting.[3] To address this issue, we conducted both a landmark clinical trial[4] and a recent meta-analysis[5] that have demonstrated that the ED use of ondansetron, an anti-emetic, leads to reductions in intravenous rehydration and hospitalization and is cost-effective.[6] However, the available data revealed some associations with increased diarrhea and no evidence of benefits associated with *ongoing* ondansetron use following ED discharge. Despite the lack of available data, the

provision of multiple doses of ondansetron for home use has become routine in many EDs across North America.[7] The literature has differing opinions on the topic of ongoing ondansetron use after ED discharge[8,9] and given the limited evidence supporting its use, the potential side effects and additional cost, there is an urgent need to definitively evaluate the effect of multiple doses of ondansetron in children, focusing on family-centered, post-index visit outcomes.[9]

We hypothesize that the administration of multiple doses of ondansetron will be associated with improved outcomes as compared with the administration of a single dose of ondansetron followed by placebo.

Route of Administration: Children will be administered oral medications as this route has proven to be effective, safe, pragmatic, and easy to perform in young children.

Dose: Eligible children will receive up to 6 doses of medication [ondansetron oral solution (0.15 mg/kg/dose) or placebo] every 8 hours, at the caregivers' discretion. This dose is consistent with those we have previously evaluated in a non-randomized fashion.[10]

Intervention Period: Study participants will receive enough study medication to be able to dispense the study medication following the recommended dosage regimen for a maximum of 48 hours following enrollment.

A placebo controlled trial is needed to properly assess the effect of the intervention as current standard of care recommendations detail no active medical intervention. Assuming an identical placebo is created we do not anticipate any disease or therapy specific challenges to emerge related to the control group.

2.2 BACKGROUND

Antiemetic agents are frequently used to facilitate rehydration therapy in children with vomiting associated with acute gastroenteritis (AGE). In a recent survey, over 80% of physicians routinely administer antiemetics to children who fail oral fluid challenge, regardless of training (pediatric vs. non-pediatric) or setting (community vs. academic center).[11] Use appears to be maximal (87%) among physicians working in pediatric emergency department (ED) with ondansetron being the antiemetic of choice identified by 99% of pediatricians.[11]

A 2013 overview[12] identified four studies that evaluated the efficacy of single dose oral ondansetron compared to placebo in children with gastroenteritis presenting to an ED for medical care in developed countries. Oral ondansetron administration resulted in reductions in the hospital admission and intravenous rehydration rates [relative risk (RR) = 0.40, 95% confidence interval (CI) 0.19, 0.83; number needed to treat (NNT) = 17, 95% CI 13, 60 and RR = 0.41, 95% CI 0.29, 0.59; NNT = 5, 95% CI 4, 8, respectively]. Following discharge, ED revisit rates were similar between the ondansetron and placebo groups. Among the subgroup of children who did revisit an ED, those administered ondansetron at the index visit were less likely to receive intravenous rehydration at the revisit.

An additional open-label clinical trial published in 2013 evaluated the efficacy of oral ondansetron, compared with domperidone, in 73 children presenting with gastroenteritis.[13] The study was powered to detect a reduction in the proportion of children who vomited in the 24h following randomization from 50% (domperidone) to 14% (ondansetron). Despite reporting a clinically relevant reduction of vomiting from 56% to 38% (32% relative risk reduction), this did not achieve statistical significance (P = 0.16).

Notwithstanding the benefits observed in clinical trials, recent database studies have reported less dramatic benefits associated with ondansetron administration. A cross-sectional analysis of over 160000 ED visits for AGE at twenty one US-based pediatric institutions between July 2009 and June 2011 documented ondansetron administration in 52% of the visits.[14] However, there was no significant correlation between oral ondansetron

administration and the administration of intravenous rehydration (P = 0.39). In a follow-up study[15] employing the same database, the authors looked at 804 000 AGE visits between 2002 and 2011. Hospital-level analyses were performed to assess the associations between ondansetron administration and rates of intravenous rehydration, hospitalization, and ED revisits within 3 days from discharge. Although ondansetron administration increased from a median rate of less than 1% to 42%, intravenous rehydration rates only declined from 19% to 18% during the same period. Although there was no change in hospitalization rates, 3-day ED revisits modestly declined (adjusted percentage change = -0.31%; 95% CI, -0.49, -0.13%). The latter study was accompanied by an editorial[16] that explored the potential reasons behind the 'failed implementation' of ondansetron into clinical care. Possibilities include: first, the medicine is not being provided to the best candidate patients. This is evidenced by the administration of oral ondansetron to only 14% of the children receiving intravenous rehydration; second, ondansetron administration has not adequately been incorporated into bundled care pathways; third, the timing of delivery may be optimized in relation to medical evaluation and decision making.

The effectiveness of ondansetron is supported by an Irish study[17] that assessed the efficacy of ondansetron for decreasing the need for intravenous rehydration in children with gastroenteritis who failed oral rehydration. The authors compared data collected over a 6-week period in 2009 (ondansetron period) with a similar period in the preceding year (no antiemetic) and noted that the proportion of children with gastroenteritis who received intravenous rehydration was reduced from 41% to 22%.

While ondansetron is widely used as an adjunct in the treatment of AGE by pediatric ED physicians,[18] sparse literature exists on the utility of discharging patients with an at-home oral dose of ondansetron to be used as prophylaxis for recurrent emesis. The symptomatic period of AGE can exceed seven days.[19] Presumably, a proportion of patients who had taken ondansetron in the ED and were asymptomatic at the time of discharge may develop recurrent emesis at home. As a result, they may ultimately return to the ED, leading to increased use of hospital resources. In Canada, in 2010, the point-of-care administration of ondansetron is estimated to cost $12.86 while an ED visit costs $229.93, not including intravenous catheter insertion ($84), possible hospitalization ($955/admission) or travel expenses for patients ($21.59).[6]

A recent small study found that 24% of pediatric patients who were treated with a single dose of ondansetron for acute gastroenteritis in the ED reported experiencing recurrent emesis within 24 hours of discharge.[20] We hypothesize that the use of a physician-dispensed, as needed home dosing regimen of ondansetron given to patients (in anticipation of recurrent emesis and risk of dehydration) may prevent a return to the ED. While a small 2010 study found no reduction in ED revisits after discharging patients' home with a written prescription for ondansetron, the authors caution that they did not track the number of patients who actually filled their prescriptions.[21] In 2002, Ramsook et al.[22] conducted a single-centre, double-blinded, placebo-controlled trial involving 145 children, comparing six doses of ondansetron/placebo administered every 8 hours for two days (scheduled). Vomiting was reduced after the first dose was administered; however, no further benefit was seen following discharge (vomiting occurred in 42% administered ondansetron versus 46% administered placebo at 24 hours). In addition, at the 24 hour and 48 hour follow-ups, the median number of vomiting episodes remained zero, with no statistically significant difference between groups. During the 48 hours following ED discharge, children administered ondansetron had threefold more diarrhea than those in the placebo group (mean 7.7 versus 2.3 episodes). The revisit rate was also higher in the ondansetron group (P=0.05). Of the four patients with revisits in the ondansetron arm, two returned with persistent vomiting and the other two with persistent diarrhea. Interestingly, providing a home dose of ondansetron to patients by the ED physician as part of discharge planning may/ promote compliance on an outpatient basis.[20]

A second double-blinded, placebo-controlled trial of relevance was published by Yilmaz et al.[23] in 2010, in which the authors provided participants (N=109) with ondansetron/placebo every 8 hours for a total of 24 hours. In this study, although ondansetron administration reduced the frequency of vomiting following discharge (mean of 1.7 versus 0.2 episodes over 24 h; P<0.001), its administration was also associated with an increase in the number of

diarrheal episodes (5.0 versus 4.3 episodes; P=0.04). Of note, there was no difference in study groups with regards to ED return visits (ondansetron, 13%; placebo, 14%; P=0.85).

The proposed comparative effectiveness study will inform a key step in the healthcare provided to children with AGE by providing clarity to the debate surrounding the use of ondansetron therapy following ED discharge. While ondansetron is widely used as an adjunct in the treatment of AGE by pediatric ED physicians, with use have increased dramatically over the past 10 years,[15] very limited literature exists evaluating the impact of discharging patients with a prescription for additional doses of ondansetron to be administered at home to prevent recurrent emesis. The potential benefits that could be accrued from the administration of ondansetron at home include 1) a reduction in nausea (never previously tracked) and vomiting (nearly 50% of children who present to the ED with vomiting continue to vomit in the 24 hours following ED discharge)[22]; 2) reduction in ED revisits (1 in 6 children treated in a Canadian ED with AGE will experience a revisit within 7 days)[24]; 3) reassurance and reduced caregiver stress/distress (if ED ondansetron worked and helped their child feel better – they often worry about the vomiting recurring); 4) reduction in cost (single dose has been shown to be cost-effective; reducing revisits also likely to be cost-effective).[6]

2.3 RISK/BENEFIT ASSESSMENT

2.3.1 KNOWN POTENTIAL RISKS

The overall risk associated with oral ondansetron administration is exceedingly low. In a post-marketing analysis and systematic review of the published literature, grey literature, manufacturer's database, Food and Drug Administration Adverse Events Reporting System, and the World Health Organization Individual Safety Case Reports Database (VigiBase); no documented (or perceived) arrhythmias were identified within 24 hours of single oral dose ondansetron administration.[25] Sixty unique reports were identified describing an arrhythmia associated with ondansetron administration (any dose, frequency). A significant medical history (67%) or concomitant use of a QT-prolonging medication (67%) was identified in 83% of reports. Approximately one third occurred in patients receiving chemotherapeutic agents, many of which are known to prolong the QT interval. An additional third involved administration to prevent post-operative vomiting.

In a retrospective study the risk of ventricular arrhythmia within 24 hours after ondansetron administration was 3 in every 100,000 patients treated annually (0.003%).[26] All 7 patients in whom an arrhythmia occurred had underlying congenital cardiac conduction abnormalities (n = 3) or other major cardiac diagnoses (n = 4). The authors concluded that the findings do not support electrocardiogram screening or continuous monitoring of other pediatric populations receiving ondansetron.

Ondansetron is a 5-HT_3 receptor antagonist indicated for the prevention of:

- nausea and vomiting associated with highly emetogenic cancer chemotherapy
- nausea and vomiting associated with initial and repeat courses of moderately emetogenic cancer chemotherapy
- nausea and vomiting associated with radiotherapy in patients receiving either total body irradiation, single high-dose fraction to the abdomen, or daily fractions to the abdomen
- postoperative nausea and/or vomiting

Contraindications include:

- known hypersensitivity (e.g., anaphylaxis) to ondansetron or any components of its formulation
- concomitant use of apomorphine (a non-opioid medication indicated for the treatment of vomiting or Parkinson's disease)

Warnings and Precautions:

- QT interval prolongation and Torsades de Pointes: Avoid in patients with congenital long QT syndrome; monitor with electrocardiograms (ECGs) if concomitant electrolyte abnormalities, cardiac failure or arrhythmias, or use of other QT prolonging drugs;
- Serotonin syndrome: Reported with 5-HT_3 receptor antagonists alone but particularly with concomitant use of serotonergic drugs;
- Masking of progressive ileus and/or gastric distention following abdominal surgery or chemotherapy-induced nausea and vomiting

Adverse reactions most frequently reported are headache, constipation, feeling of warmness, and flushing[27]. There have been rare reports of tachycardia, angina (chest pain), bradycardia, hypotension, syncope, electrocardiographic alterations, seizures, rash, and hypersensitivity reactions.

Taking in to account our carefully chosen inclusion and exclusion criteria, the risk of adverse events is low in the population of children who will be enrolled in this study. Given the broad use of ondansetron, and the potential benefit associated with the outpatient use of this medication following ED discharge, it is vital that we determine if any benefit is accrued related to its use.

2.3.2 KNOWN POTENTIAL BENEFITS

Antiemetic agents are frequently used to facilitate rehydration therapy in children with vomiting associated with AGE. In a recent survey, over 80% of physicians routinely administer antiemetics to children who fail oral fluid challenge, regardless of training (pediatric vs. non-pediatric) or setting (community vs. academic center).[11] Use appears to be maximal (87%) among physicians working in pediatric EDs with ondansetron being the antiemetic of choice identified by 99% of pediatricians.[11]

A 2013 overview[12] identified four studies that evaluated the efficacy of oral ondansetron compared to placebo in children with gastroenteritis presenting for medical care in developed countries. Oral ondansetron administration resulted in reductions in the hospital admission and intravenous rehydration rates [relative risk (RR): 0.40, 95% confidence interval (CI) 0.19, 0.83; number needed to treat (NNT): 17, 95% CI 13, 60 and RR: 0.41, 95% CI 0.29, 0.59; NNT: 5, 95% CI 4, 8, respectively]. Following discharge, ED revisit rates were similar between the ondansetron and placebo groups. Among the subgroup of children who did revisit an ED, those administered ondansetron at the index visit were *less* likely to receive intravenous rehydration at the revisit.

An updated systematic review and meta-analysis for ondansetron use was published in 2016.[28] This review identified 10 RCTs involving 1,215 participants. Treatment with ondansetron compared with placebo increased the chance for vomiting cessation up to 1 hour after drug administration, relative risk, RR, 1.49 (95% confidence interval 1.17–1.89), but there was no difference between the groups after 4, 24 and 48 hours. Treatment with ondansetron compared with placebo reduced the risk of failure of oral rehydration therapy, RR 0.5 (0.37–0.69), increased the intake of oral rehydration solution in 1 hour and 4 hours, mean difference: 43 mL/1 h (15.5–70.5), and 91 mL/4 h (35–147), respectively, reduced the risk of hospitalization, RR 0.53 (0.29–0.97), and reduced the need for intravenous rehydration, RR 0.45 (0.31–0.63); however, it had no effect on the need for return visits to the ED, RR 1.14 (0.72–1.8). Adverse effects were similar in both groups. The authors concluded that current evidence supports oral administration of ondansetron to increase the success rate of oral rehydration therapy, and this intervention should be endorsed in the management of children with acute gastroenteritis treated in emergency departments.[28] Currently, no strong evidence currently exists for at-home use of oral ondansetron in children with vomiting secondary to acute gastroenteritis.

2.3.3 ASSESSMENT OF POTENTIAL RISKS AND BENEFITS

The main risks associated with this protocol are associated with the administration of oral ondansetron. Study questionnaires are designed to minimize questions which may be perceived as intrusive. To minimize risk associated with the administration of multi-dose ondansetron, the study inclusion and exclusion criteria were carefully selected to avoid known contraindications. Patients who have a known hypersensitivity or allergy to ondansetron or other serotonin receptor antagonist, a history of prolonged QT syndrome or presence of heart disease or arrhythmia, as well as individuals taking SSRI, SNRI, apomorphine, macrolide antibiotics or any medication accepted as causing torsades de pointes[27] will be excluded from participation in the study.

Study medication will be administered at home by the participants' caregiver on an 'as needed' basis. Doses will be based on the weight of the participant at time of enrollment. Trained study research staff will calculate the appropriate dose of study medication to be administered. The dose information will be recorded on discharge instructions provided to the caregiver and the volume of study medication will be marked on a syringe to minimize measuring errors. Research staff will provide one-on-one teaching and printed instructions on how to administer the doses to the participant prior to participant discharge from the ED to minimize the risk of incorrect dosing.

Participants will be contacted 24, 48, and 168 hours (7 days) after randomization to obtain outcome endpoint data and determine if there have been any adverse events (e.g. diarrhea, constipation, headache, etc.). Data regarding ongoing vomiting, diarrhea, and/or fever, and return to healthcare will be collected during scheduled follow-up calls/emails. Caregivers can also provide general information regarding the child's illness or any other symptoms the child may be experiencing when completing the scheduled follow-up questionnaires. This information will be reviewed by a trained research team member for any possible adverse events or symptoms that may require medical care.

3 OBJECTIVES AND ENDPOINTS

The primary objective is to determine if the use of multi-dose oral ondansetron compared with placebo, provided at ED discharge, will result in improved outcomes in children treated in an Emergency Department for vomiting secondary to acute gastroenteritis.

The primary outcome is the development of moderate-severe disease in the 7 days after the index ED visit as measured by the MVS [29,30]. The original 20 point Vesikari Score has been employed as a dichotomous variable in many clinical studies despite limited evidence supporting its use. However, it has been shown to correlate with other meaningful measures such as caregiver anxiety, helplessness, and stress. Recently, increasing severity scores were associated with higher caregiver worry, greater changes in the child's behavior, and trends towards greater impact on the caregivers' daily activities and higher caregiver distress. Moreover, two large multi-centre ED based RCTs have used the MVS as primary outcomes, both published in the New England Journal of Medicine in November 2018.[31,32]

Characteristics of the MVS: We prospectively evaluated the MVS in an 11 centre (455 children) ED study[29] which found that it effectively measures global disease severity. Factor analysis revealed that item correlations were acceptable and supported the appropriateness of retaining all factors. Multi-collinearity was not a problem and the correlations between the MVS and other measures of clinical significance were in the expected direction. Disease severity was associated with prolonged daycare (P = 0.01) and work (P = 0.002) absenteeism. The MVS had a normal distribution with minimal kurtosis (-0.14; SE: 0.24) and skewing (0.39; SE: 0.12). There was good variation

across severity ranges (49% mild; 21% moderate; 30% severe). Variation between institutions was insignificant (P = 0.11) and complete follow-up was achieved in 91% of participants.

How will it be calculated? Once follow-up is complete (Day 7), each variable is assigned a score for the entire study period (Time 0 to Day 7). Each participant gets a single total score for the study. Variables are scored based on the worst 24 hour period (e.g. maximal number of episodes of vomiting in a 24 hour period), the total duration of symptoms (e.g. number of days of vomiting), or are based on the occurrence of an outcome (e.g. hospitalization).

What if at baseline the pre-enrollment MVS is ≥ 9? Regardless of the score assigned at Time 0 (i.e. *pre-enrollment score*), EVERYONE reverts to a score of 0 at enrollment (i.e. the study evaluates the impact on the disease process going forward). The *pre-enrollment score*, which is based on symptoms prior to presentation, will serve as a covariate in a secondary analysis of the primary outcome and will be employed for sub-analysis purposes. **The primary outcome** *(the presence of moderate-severe disease, as defined by a MVS of ≥ 9 during the 7 day follow-up period)* **will ONLY include symptoms and outcomes that occur <u>following</u> the ED visit (i.e. after enrollment) and will not be directly impacted by the *pre-enrollment score*.**

Why a cut-point of 9? With the original score, severe disease was defined as ≥ 11; moderate as ≥ 9. In our derivation study, construct validity was proven by using scores of ≥ 9 to define moderate and ≥ 11 to define severe disease. These cut-points were associated with significant increases in other measures of disease severity [e.g. daycare (P=0.01) and work absenteeism (P=0.002). [7]

Outcome measure data will be collected using participant follow-up questionnaires performed 24, 48, and 168 hours (7 days) after enrollment (Time 0 = consent date and time). At the enrollment visit (Day 0), all caregivers will be asked their preferred method of communication for follow-up: email or telephone. Following discharge from the ED, trained study staff will contact caregivers for the duration of the follow up period as outlined in Section 1.3 SoA. The questionnaire (performed via telephone or secure web based link) will inquire about ongoing symptoms, medical evaluations, treatments, child care, resource utilization, and side effects. If speaking by telephone, detailed questioning will follow positive responses. The web based questionnaire will employ advanced logic to enhance ease of use. If the caregiver does not complete the online questionnaire within 24 hours, a telephone follow-up will be performed by study staff to obtain outcome data. Final data points will be collected from the caregiver after 168 hours (7 days). The follow-up questionnaire will also assess study intervention administration (ondansetron oral solution) at the 24 and 48 hour follow-up assessments. The study team will closely track how many doses the caregiver administered to the child by ensuring the questionnaires are completed in full. If the email questionnaire is not completed in full, a telephone call will be performed to obtain the missing data. If electronic follow-up is chosen, if the questionnaire is not completed within 24 hours of receipt, the study team will switch to phone follow-up to maximize the response rate and minimize recall bias.

The **presence and frequency of vomiting** will be measured following enrollment in the study using follow up questionnaires 24, 48, and 168 hours (7 days) after Time 0. Questionnaires (telephone or email) will contain questions regarding vomiting presence, number of episodes, and vomiting cessation date and time. In previous work, we have concluded that vomiting frequency is a predictor of outcomes in AGE[33].

Return visits for unscheduled care to a health care provider related to vomiting, diarrhea, dehydration, fever, abdominal pain, or fluid refusal within 7 days, not including scheduled visits (e.g. reassessments, vaccinations), will also be measured using follow up questionnaires 24, 48, and 168 hours (7 days) after Time 0. Questionnaires will ask caregivers if they brought their child to a health care provider for an unscheduled visit related to the current illness. If a return visit occurred, further questions will be asked related to **Intravenous (IV) insertion**, treatment course, and disposition. Return to care data will be confirmed by reviewing the participant's hospital chart, where

possible. This outcome is important as > 50% of children have a follow-up office visit, [34] 8-18% require an ED visit,[35] and 5-8% are hospitalized.[34]

A **5-point Likert scale will be used to determine caregiver satisfaction** with the therapy provided (ondansetron or placebo oral solution) in the 48 hours following Enrollment in the study.

The following **secondary safety endpoints** are important as they will add more information about the secondary objective. The safety endpoints will include:

1) Serious Adverse Events (SAEs)
2) Frequency of diarrheal episodes during the 48-hours following ED disposition
3) Maximal number of diarrheal episodes in 24 hours
4) Palpitations
5) Pre-syncope/syncope
6) Chest Pain
7) Arrhythmias

All safety end point data will be obtained via participant follow-up questionnaires performed 24, 48, and 168 hours (7 days) after enrollment (Time 0). **Frequency and maximal number of diarrheal episodes** will be obtained by asking about presence of diarrhea on each questionnaire the caregiver completes. If diarrhea is present, further questions related to number of episodes will be asked. To obtain SAE and palpitation data, each questionnaire will contain open ended, non-symptom specific questions where caregivers can provide the study team with information about their child's illness or any other concerns they may have. Any response provided to this question will result in a trained study team member discussing the information provided in more detail via telephone. Any symptom or condition noted that meets the definition of an SAE (see Section 8.3.2) will be reported as outlined in Section 8.3.6.

Table 1.0 – Summary of Objectives and Endpoints

OBJECTIVES	CANDIDATE ENDPOINTS	JUSTIFICATION FOR ENDPOINTS
**Primary**		
To determine if in previously healthy children, who present to an ED with gastroenteritis associated vomiting; is the proportion who ***develop moderate to severe disease*** [MVS ≥ 9] following ED evaluation significantly different in those who receive home ondansetron compared to those who receive placebo.	Post-index visit Modified Vesikari Scale (MVS) score in the 7 days following ED disposition[29 30]	The MVS is a composite measure which includes several aspects which represent the severity of disease. The score incorporates features of gastroenteritis that are of important to a variety of stakeholders including caregivers, children and physicians (e.g. duration of diarrhea and vomiting, frequency of diarrhea and vomiting, maximal height of fever, the presence of dehydration and the medical interventions required).
**Secondary**		

Table 1.0 – Summary of Objectives and Endpoints

OBJECTIVES	CANDIDATE ENDPOINTS	JUSTIFICATION FOR ENDPOINTS
Among children receiving active treatment versus placebo:<br>(1) Is there a difference in the (a) duration of vomiting, (b) frequency of vomiting, or (c) the proportion who experience vomiting following ED disposition.<br>(2) Is there a difference in the proportion who require an unscheduled health care provider visit?<br>(3) Is there a difference in the proportion who require intravenous rehydration?<br>(4) Is *there a difference in the satisfaction with care?*	1. Vomiting: Frequency, Duration, Proportion<br>2. Health Care Use: ED, Primary Care<br>3. Intravenous rehydration<br>4. Caregiver Satisfaction with Therapy Provided: Likert Scale	
**Safety**		
To determine if the discharge of children with AGE associated vomiting who are administered ondansetron in the ED with additional doses to be taken at home is associated with adverse events (e.g. diarrhea, revisits) as compared with placebo (i.e. safety).	1. Frequency of diarrheal episodes during the 48-hours following ED disposition.<br>2. Maximal number of diarrheal episodes in 24 hours<br>3. Palpitations<br>4. Syncope/pre-syncope<br>5. Chest Pain<br>6. Arrhythmias<br>7. Serious Adverse Events	It is crucial to monitor for potential adverse events associated with the introduction of additional therapies.
**Tertiary/Exploratory**		
None.	Not applicable	Not applicable

Following study completion an economic evaluation will be performed.

4 STUDY DESIGN

4.1 OVERALL DESIGN

We will conduct a Phase III multi-centre, 2-arm, placebo-controlled, double-blind, parallel design randomized controlled trial comparing the effectiveness and adverse event profile of multiple dose (as needed) oral ondansetron compared with placebo when administered to children with acute gastroenteritis (AGE)-associated

vomiting following ED discharge. The study will be stratified by site (6 sites) and weight (< 20kgs and ≥ 20kgs). Weight is being employed, rather than age, as dosing is weight based.

We hypothesize that the administration of ondansetron post-ED discharge will be associated with improved outcomes as compared with placebo.

4.2 SCIENTIFIC RATIONALE FOR STUDY DESIGN

There is currently insufficient data to determine with certainty whether additional doses of ondansetron should be administered following the ED disposition of children with vomiting secondary to AGE. While it may be beneficial, studies to date have not provided robust evidence and the potential benefits need to be balanced with the potential to increase the frequency of diarrhea.

4.3 JUSTIFICATION FOR DOSE

A standardized dose of 0.15mg/kg of oral ondansetron solution will be used in this study. The maximum singular dose of oral ondansetron provided will be 8mg, and the dose to be administered will be based on the weight of the participant at time of study enrollment. Enough study medication will be provided to enable the administration of a maximum of 6 doses at home. This dosing regimen has been shown to be effective, safe and is within the dosing range (0.13-0.26 mg/kg) that has previously been used for this indication.[4,10]

A single dose of 8mg has been demonstrated to have a minimal effect on the QTc – prolonging it by only 0.4 msec.[36] Moreover, within the recommended/commonly used dose range of 0.13-0.26 mg/kg, higher doses of ondansetron have not been found to be superior to lower doses.[10]

Children will be administered oral medications as this route has proven to be effective, safe, pragmatic, and easy to use in young children.

Eligible children will receive up to 6 doses of medication [ondansetron oral solution (0.15 mg/kg/dose) or placebo] to be administered every 8 hours at the caregiver's discretion. Such caregiver-driven, after ED discharge dosing protocols are increasingly common with a recent description of a cohort of 996 children among whom 76% received ondansetron in the ED, and 71% were discharged with prescriptions for ondansetron. Moreover the median number of home doses of ondansetron prescribed was 10 (IQR: 6, 12).[7] In this cohort, 7-day ED return rates were similar between groups (6% with prescription, 5% without, P = 0.66). We will have a standardized written handout that the research staff will employ explaining when we suggest that the medication be given.

4.4 END OF STUDY DEFINITION

The end of the study is defined as completion of the last visit or procedure shown in the SoA in the trial globally.

A participant is considered to have completed the study if they have completed all phases of the study including the Day 7 email or telephone questionnaire shown in the SoA in Section 1.3. Emergency Department chart review of enrollment/baseline data is performed by study staff at each site and does not involve direct participation by the study participant or caregiver.

It is not necessary for caregivers to administer all study medication doses provided to them to remain compliant with the protocol. Doses are administered based on caregiver assessment of the child's need. The number of

doses administered by the caregiver will be recorded on the follow-up questionnaires completed by email or telephone.

5 STUDY POPULATION

5.1 INCLUSION CRITERIA

All patients who present to the ED of the six participating institutions will be assessed for eligibility. The screening process will be compliant with each study site's provincial and institutional policies and legislation.

To be eligible to participate in the study, individuals must meet **all** of the following criteria:

1. Provision of signed and dated informed consent and assent (where applicable) forms
2. Stated willingness to comply with all study procedures and availability for the duration of the study.
3. Diagnosis of acute intestinal infectious process confirmed by the treating ED physician: *The diagnosis of gastroenteritis/acute intestinal infectious process is at the discretion of the emergency department supervising physician. Alternative terminologies that reflect a similar diagnosis are acceptable provided they meet all other eligibility criteria. Examples include: viral illness, diarrhea, vomiting, upper respiratory infection, post-infectious gastroenteritis, antibiotic associated diarrhea, toddlers diarrhea, viral infection, enteritis, viremia, fever, and bronchiolitis.*
4. Child/youth aged 6 months to 17.99 years.
5. Presence of ≥ 3 episodes of vomiting in the preceding 24 hour period (in the 24 hours preceding the screening process performed by the research team).
6. Duration of vomiting and/or diarrheal symptoms < 72 hours (*a 24 hour vomit and diarrhea free interval denotes a separate illness and only continuous days since the last 24 hour vomit and diarrhea free period are included*).
7. A minimum of 1 episode of vomiting within 6 hours of the screening process performed by the research team (time of approach and screening to be recorded): Date of time of assessment of eligibility for this study.
8. A minimum of 1 dose of ondansetron (oral or intravenous) provided during the current emergency department visit.

Due to the nature of the illness being studied and the fast paced environment of the Emergency Department, preliminary screening may occur prior to obtaining a final diagnosis and/or an order for ondansetron.

5.2 EXCLUSION CRITERIA

Research staff will ensure selection of participants is fair and equitable in accordance with Article 4.1 of the Tri Council Policy Statement: "*Researchers shall not exclude individuals from the opportunity to participate in research on the basis of attributes such as culture, language, religion, race, disability, sexual orientation, ethnicity, linguistic proficiency, gender or age, unless there is a valid reason for the exclusion*"

In this study, language barrier may be listed as an exclusion criteria due to the amount of communication needed for the follow-up portion of the study and the potential risks associated with administration of ondansetron. If the participant is unable to speak English or French, and the study team is unable to find an individual who speaks the participant's language (family member, hospital translator, etc.), they will not be consented or enrolled in the study. Similarly, because communication is a key piece of the study, if a person is unable to provide follow-up and comprehend the informed consent form they will not be able to participate in the study.

Individuals who meet **any** of the following criteria will be excluded from participation in this study:

1. Bilious (having a green or bright yellow colour, indicating larger amounts of bile in the stomach) or bloody vomit during current illness: *This may indicate an alternate diagnosis.*
2. Known hypersensitivity to ondansetron or any serotonin receptor antagonist (e.g. palonosetron, dolasetron, granisetron).
3. Known allergic reaction to components of ondansetron *(citric acid, sodium benzoate, sodium citrate dihydrate, and strawberry flavor, sorbitol)*[27] or the placebo medication *(methylparaben, glycerin, citric acid, potassium sorbate, sorbitol, strawberry flavor)*
4. History or family history (first degree relative) of prolonged QT syndrome
5. Presence of complex congenital heart disease
6. History or family history (first degree relative) of cardiac arrhythmia
7. Concomitant use of (within the past 48 hours OR being prescribed) any of the following – *For a full list of possible medications in the following categories, please references the TMOP:*
    a) QTc prolonging medications[27] (e.g. methadone, anti-depressants, antiarrhythmics)
    b) Medications known to cause torsades de pointes[27] (e.g. macrolide antibiotics, apo morphine (*a morphine decomposition product that does not contain morphine or it's skeleton, nor does it bind to opioid receptors; acts as an antagonist of 5-HT$_2$ and α-adrenergic receptors with high affinity*[37]
    c) Medications that cause electrolyte abnormalities[27] (e.g. high dose corticosteroids, diuretics)
    d) Serotonergic or neuroleptic medications: *Risk of serotonin syndrome or neuroleptic malignant syndrome*[27] (e.g. triptans, SSRIs, SNRIs, fentanyl, MAOIs, lithium)
    e) Any 5-HT$_3$ receptor antagonist excluding ondansetron: C*ross reactivity has been reported between different 5-HT$_3$ antagonists*[27]
8. Unable to complete follow-up due to travel, no telephone or internet availability, or a language barrier beyond reasonable accommodation.
9. Previously enrolled in this study.
10. History or family history (first degree relative) of G6PD deficiency: *Due to the small amount of quinine present in the placebo compound which in theory can trigger a hemolytic crisis.*

5.3 LIFESTYLE CONSIDERATIONS

Not Applicable.

5.4 SCREEN FAILURES

Screen failures are defined as participants who are assessed for eligibility to participate in the clinical trial but are not subsequently randomly assigned to the study intervention or entered in the study. A minimal set of screen failure information (e.g. age in months, sex, eligibility criteria, and consent status) is required to ensure transparent reporting of screen failure participants, to meet the Consolidated Standards of Reporting Trials (CONSORT) publishing requirements and to respond to queries from regulatory authorities. Data collected from screen failures will be presented in aggregate form.

Re-screening of patients excluded will not occur during the same ED visit however the same patient may be re-screened during subsequent ED visits if presenting for a new illness. Once a patient is randomized, they will be included in the original sample size and their data will be analyzed.

5.5 STRATEGIES FOR RECRUITMENT AND RETENTION

Participants will be recruited from six EDs across Canada. Patients will be approached for permission to participate in research according to local policy and procedure. Only patients who have been administered a minimum of 1 (one) dose of ondansetron by the clinical team will be approached for participation in the study.

To ensure study retention, trained research study staff will review the study in detail with the patient and/or caregiver emphasizing the risk, benefits, and time commitment required prior to consent.

Follow up will be performed 24 and 48 hours following enrollment, as well as on Day 7 (168 hours after enrollment). Caregivers will have the opportunity to complete follow-up by email or telephone. If no response is received via email within 24 hours of the email send time, a follow-up telephone call will be performed to obtain the necessary data. If unable to make contact via telephone within the first 24 hours, 3 (three) additional attempts per follow-up time point will be made to contact the caregiver and/or participant by telephone over the subsequent 5-14 days.

There will be no financial reimbursement associated with this trial.

For a more detailed description of recruitment and retention plans, see the TMOP and site specific SOPs.

6 STUDY INTERVENTION

6.1 STUDY INTERVENTION(S) ADMINISTRATION

6.1.1 STUDY INTERVENTION DESCRIPTION

Consenting participants will be randomized to receive either ondansetron oral solution (4mg/5mL) or a matching placebo oral solution.

Intervention: Ondansetron oral elixir 4mg/5mL Oral Solution, USP administered at a dosing rate of 0.15mg/kg [Appendix A and B]

Comparator: Matching placebo oral solution [Appendix B and C] administered in the same weight-based volume (mL) as the intervention dose *(contains no active intervention ingredients)*

6.1.2 DOSING AND ADMINISTRATION

Participants will be randomized to receive either ***ondansetron oral solution at a dosing rate of 0.15mg/kg*** or an identical placebo oral solution (containing no active ingredients). Ondansetron oral solution will be provided in 4mg/5mL concentrations.

Study medication will be provided to the caregiver of the participant in sufficient quantity to last 48 hours. Medication can be administered ***every 8 hours (q8h), to a maximum of 3 times a day (24 hour period (TID)),*** as deemed necessary by the participant's caregiver(s).

Participants will not be required to take all 6 doses of the study intervention provided. They will receive doses based on need, as determined by the participant's caregiver. Caregivers will be asked to return unused study medication to the study team. All unassigned study medication will be returned to the study pharmacy for destruction.

6.2 PREPARATION/HANDLING/STORAGE/ACCOUNTABILITY

6.2.1 ACQUISITION AND ACCOUNTABILITY

Zofran® or generic ondansetron oral solution, USP in 4mg/5mL in 50mL bottles manufactured by Sandoz Canada (Zofran®)or JAMP Pharmaceuticals (ondansetron) will be acquired locally by site research pharmacies based on availability; re-packaged in amber bottles and labelled according to section 6.2.2 below (as detailed in the study pharmacy manual). Zofran® should be prioritized for use, if available.

A matching placebo oral solution has been formulated by the University of Manitoba, College of Pharmacy and the Alberta Health Services Research Pharmacy for both Zofran® and the generic ondansetron oral solution. Placebo formulations will be provided to each participating site research pharmacy; where they will be compounded, packaged, and labelled to match the active medication.

The placebo and ondansetron oral solutions will be placed in matching bottles and labelled identically to ensure blinding.

Dispensation of the study intervention from site pharmacies to research staff will be tracked by each research pharmacy on a drug accountability and dispensation log. Dispensation to the caregiver from the research team will be tracked by the research team on study CRFs and drug accountability logs.

For further details on investigational product accountability, please reference the study TMOP or Study Pharmacy Manual.

6.2.2 FORMULATION, APPEARANCE, PACKAGING, AND LABELING

Active Study Medication:
Product: Ondansetron Oral Solution, USP
Manufacturer: Sandoz Canada, a division of Novartis or JAMP Pharmaceuticals, Canada (based on availability)

Summary Product Information – Active		
**Route of Administration**	**Dosage Form / Strength**	**Nonmedicinal Ingredients**
Oral	oral solution/<br>4 mg/5 mL ondansetron (as hydrochloride dihydrate)	citric acid, sodium benzoate, sodium citrate dihydrate, sorbitol, strawberry flavour (contains ethanol)

Ondansetron hydrochloride is a selective antagonist of the serotonin receptor subtype, 5-HT_3. Its precise mode of action in the control of nausea and vomiting is not known.

Pharmacodynamics: In vitro metabolism studies have shown that ondansetron is a substrate for human hepatic cytochrome P450 enzymes, including CYP1A2, CYP2D6 and CYP3A4. In terms of overall ondansetron turnover, CYP3A4 played the predominant role. Because of the multiplicity of metabolic enzymes capable of metabolizing ondansetron, it is likely that inhibition or loss of one enzyme (e.g. CYP2D6 enzyme deficiency) will be compensated by others and will result in little change in overall rates of ondansetron clearance.

Pharmacokinetics: Pharmacokinetic studies in human volunteers showed peak plasma levels of 20-30 ng/mL at around 1½ hours after an 8 mg oral dose of ondansetron. An 8 mg infusion of ondansetron resulted in peak

plasma levels of 80-100 ng/mL. Repeat dosing of an 8 mg tablet every 8 hours for 6 days increased the peak plasma value to 40 ng/mL. A continuous intravenous infusion of 1 mg/hour after the initial 8 mg loading dose of ondansetron maintained plasma levels over 30 ng/mL during the following 24 hour period. The absolute bioavailability of ondansetron in humans is approximately 60% and the plasma protein binding is approximately 73%. Following oral or IV administration, ondansetron is extensively metabolized and excreted in the urine and feces. In humans, less than 10% of the dose is excreted unchanged in the urine. The major urinary metabolites are glucuronide conjugates (45%), sulphate conjugates (20%) and hydroxylation products (10%).

The half-life of ondansetron after either an 8mg oral dose or intravenous dose was approximately 3-4 hours and may be extended to 6-8 hours in the elderly.

Study Comparator
Product: Placebo Oral Solution
Manufacturer/Supplier: Formulation provided by the University of Manitoba, College of Pharmacy. Compounding completed locally at participating site pharmacies.

Summary Product Information – Comparator		
**Route of Administration**	**Dosage Form / Strength**	**Nonmedicinal Ingredients**
Oral	oral placebo solution/<br>4 mg/5 mL	methylparaben, glycerin, citric acid, potassium sorbate, sorbitol, strawberry flavour, quinine concentrate (purified or sterile water)

A Health Canada CTA is not required for this study however compliance with the Food and Drug Act will be maintained. Per Health Canada's Food and Drug Act (C.05.011), the study drugs and placebos will bear a label that sets out the following information in both official languages:

(a) a statement indicating that the drug is an investigational drug to be used only by a qualified investigator;
(b) the name, number or identifying mark of the drug;
(c) the expiration date of the drug;
(d) the recommended storage conditions for the drug;
(e) the lot number of the drug;
(f) the name and address of the sponsor; and
(g) the protocol code or identification;

In addition, the following records will be maintained pertaining to the study drug:

- A copy of all versions of the product monograph and investigator brochure, if applicable
- Records respecting the shipment, receipt, position, return, and destruction of the drug

For more information regarding formulation and stability, please reference the Zofran® andOndansetron Oral Solution 4mg/5mL product monographs (Appendix A and B).

6.2.3 PRODUCT STORAGE AND STABILITY

Ondansetron oral solution (4mg/5ml) and the matching placebo should be kept in the bottles provided and stored upright between 15°C and 30°C and should not be refrigerated.

The study medication labelled as Investigational Product will be stored in the pharmacy or ED of the participating institutions in a locked cabinet or secured room with limited access.

For further detail regarding dispensing procedures and expiration dates, please reference the study TMOP and/or the pharmacy manual.

6.2.4 PREPARATION

Site research pharmacies will order the required number of bottles of Ondansetron Oral Solution available commercially in 50mL bottles. Empty bottles will be ordered by each institutional pharmacy as detailed in the pharmacy manual prepared by the coordinating Alberta Health Services Research Pharmacy. The matching placebo will be compounded locally at the individual site research pharmacy according to the placebo formulation provided by the University of Manitoba, College of Pharmacy. The active medication will be re-packaged in to the empty bottles to match the packaging of the placebo solution. The bottles will be labelled according to section 6.2.2. Study product will be stored in the local site pharmacy until dispensed to the research team. A treatment allocation list will be provided to the pharmacy by a biostatistician and uploaded to randomize.net (see Section 6.3). To maintain blinding, research pharmacies will label each bottle(s) with an individual kit number matching the treatment allocation list provided.

Once assigned via randomization to the participant, a research study team member will enter the patient's weight in to the secure online study database where a weight based dose will be calculated. The research nurse will write the calculated dose (in milligrams (mg) and corresponding volume (mLs)) on the discharge instructions and dose tracking form for the caregiver to take home. The research nurse will describe to the caregiver how to draw up and administer a dose of the study medication. Doses will be measured using an oral syringe, directly from the bottle of study medication. Oral syringes and clear instructions will be provided to the caregivers prior to leaving the ED.

For further details regarding formulation, dispensing procedures and expiration dates, please reference the study TMOP and/or the pharmacy manual.

6.3 MEASURES TO MINIMIZE BIAS: RANDOMIZATION AND BLINDING

Sequence Generation: A biostatistician will generate a randomization list stratified by site and weight, which will then be uploaded to an internet based randomization service that uses random-number generating software. The lists will be sent to the central research pharmacy located in Calgary, Alberta who will disseminate the lists to the appropriate site research pharmacies. Kits will be prepared and labelled on site at each participating institutional research pharmacy. The kits will be stored in the local site research pharmacies until dispensed to the ED for recruitment needs.

Allocation Concealment: Randomize.net uses industry standard security to send data over the internet. Randomization will be blocked using random blocks of 4 and 6 with a 1:1 allocation ratio. Stratifying by clinical site and weight and blocked randomization will ensure that variations (e.g. site specific practice patterns, gastrointestinal pathogens) are comparably distributed across treatment arms. Only the research pharmacy at the coordinating centre and www.randomize.net will retain the randomization code.

Implementation: Potentially eligible patients will be screened. A log of all screened patients will be maintained. If eligible, the details of the study will be discussed with the caregivers and potential patients. If consent is obtained, enrolled participants will be assigned a participant ID number by the clinical site via REDCap (i.e record ID). Baseline demographic clinical variables will be collected and the data collection forms will be completed either on paper or directly into the secure online REDCap database via electronic tablet. The research team member will

then log into randomize.net which will randomize the patient (i.e. it will provide a kit number that corresponds to a study drug kit at the clinical site which will be given to the patient).

Emergency Unblinding*:* Un-blinding should only occur when it is felt by the treating physician that unblinding would alter the clinical care being administered. All participants whose therapy is intentionally un-blinded will discontinue the experimental therapy. Un-blinding should only occur when future clinical treatment of the patient will depend on prior treatment administered. Approval from the principal investigator or designate will be obtained prior to un-blinding. If the principal investigator cannot be reached, the un-blinding can be performed and the principal investigator informed within 24 hours via e-mail or telephone call. Accidental and intentional un-blinding will be documented and reported however, the study team will continue to follow the participant according to the SoA in Section 1.3. In addition to the research pharmacy, the study medical monitor will have access to a randomize.net login and password to unblind treatment should the research pharmacy be unavailable to do so.

6.4 STUDY INTERVENTION COMPLIANCE

After randomization and treatment assignment, the qualified research team member will complete a study medication dispensation form which contains the blinded kit number, expiration date, participant study ID, participant weight and age at time of enrollment, dose dispensed, the name and signature of the dispensing staff member, and the date and time of dispensation.

A drug accountability log will also be maintained at all participating institutions containing the initials of the qualified research team member dispensing the intervention to the participant caregiver, the kit number used, the accompanying participant study ID and date and time of dispensation.

The qualified research team member will demonstrate to the caregiver how to measure and dose the study medication. The exact volume in milliliters of the 4mg/5mL solution will be calculated by the research team member using REDCap prior to the caregiver leaving the ED.

The caregiver will be instructed to provide one dose every 8 hours to a maximum of 3 doses in a 24 hour period for 48 hours after discharge from the ED, as they feel their child requires the intervention. **The caregiver may choose to administer a minimum of zero to a maximum of six doses (excluding vomited doses) to the participant. If the caregiver does not administer any doses of the study intervention; the participant is still considered compliant with the study protocol**.

The number of doses administered to the participant by the caregiver will be recorded via telephone or email questionnaire performed 24 and 48 hours after enrollment (*Time 0 – Consent Date and Time*). Caregivers will be asked to return unused study medication and/or empty bottles to the study team or to a community pharmacy for destruction after the completion of the 7 day follow up period.

The participant will be considered not compliant with the protocol if the study team is unable to contact the caregiver/participant for the follow-up schedule as detailed in the SoA (section 1.3) to a maximum of 21 days after enrollment.

6.5 CONCOMITANT THERAPY

While visiting the ED, additional medications may be administered to the participant as per the treating physician, EXCLUDING ondansetron. Medication information will be documented on the patient medical record, as well as

the study case report forms. Medications to be reported on the Case Report Forms (CRF) are concomitant prescription medications, over-the-counter medications, and supplements.

For this protocol, a prescription medication is defined as a medication that can be prescribed only by a properly authorized/licensed clinician.

Patients who are currently taking any of the following medications will be excluded from participation in the study: SSRI's, SNRI's, apo morphine, any macrolide antibiotic, or medications accepted as causing torsades de pointes.

For eligibility determination and safety reasons, confirmation of medications will be performed prior to randomization.

6.5.1 RESCUE MEDICINE

Not Applicable.

7 STUDY INTERVENTION DISCONTINUATION AND PARTICIPANT DISCONTINUATION/WITHDRAWAL

7.1 DISCONTINUATION OF STUDY INTERVENTION

Discontinuation from the study intervention does not mean discontinuation from the study and remaining study procedures should be completed as indicated by the study protocol. If a clinically significant finding is identified after enrollment, the investigator or qualified designee will determine if any change in participant management is needed or if an AE report must be completed.

Because the study intervention may or may not be given in this trial at the discretion of the caregiver; data collection at time of study intervention discontinuation will include the following:

- All data collection points as listed on The DOSE-AGE Study Case Report Forms
- Date/time of last dose
- Any possible adverse events

The study intervention may be discontinued at any time by the participating caregiver, their physician, or by the site or qualified investigator.

Because doses of the study medication are only given to the child based on caregiver assessment of child's need, it is not mandatory to administer all doses provided therefore the protocol will not change unless discontinuation is due to an Adverse or Serious Adverse Event.

7.2 PARTICIPANT DISCONTINUATION/WITHDRAWAL FROM THE STUDY

Participants are free to withdraw from participation in the study at any time, and for any reason, upon request. The reason for voluntary withdrawal from the study will not be collected.

A site or qualified investigator may discontinue or withdraw a participant from the study for the following reasons:

- If any clinical adverse event (AE), laboratory abnormality, or other medical condition or situation occurs such that continued participation in the study would not be in the best interest of the participant

- If the participant meets an exclusion criterion (either newly developed or not previously recognized) that precludes further study participation (e.g. results in safety concerns)

The reason for participant discontinuation or withdrawal from the study will be recorded on the applicable study Case Report Form (CRF).

All participants who sign the informed consent form, and are randomized and receive the study intervention, and subsequently withdraw, or are withdrawn or discontinued from the study will not be replaced. These participants are accounted for in the sample size calculation. Data collected up to the point of withdraw will be analyzed unless specifically stated otherwise by the participant/caregiver.

All adverse event data will be maintained and analyzed for safety purposes.

7.3 LOST TO FOLLOW-UP

Participants are considered lost to follow up if they fail to complete the 168 hour follow up time-point and are unable to be contacted by the study site staff within the timeframe provided in the SoA (section 1.3).

To minimize the number of participants that are lost to follow up, there will be no in-person visits and minimal telephone/email follow-up.

The following actions must be taken if a participant fails to complete study follow up procedures:

- The site will attempt to contact the participant within 24-48 hours of the follow-up due date and counsel the participant on the importance of maintaining the assigned visit schedule and ascertain if the participant wishes to and should continue in the study. Should the participant wish to remain in the study; all retrospective data for missed follow-up time points should attempt to be collected.

- Before a participant is deemed lost to follow-up, the investigator or designee will make every effort to regain contact with the participant via telephone and/or email.
  - If the participant chose **email follow-up**: If no response is received within 24 hours of the initial follow-up questionnaire email send date and time, the study team will attempt to contact the participant by telephone a maximum of 3 attempts per missed follow up (maximum of 9 attempts to contact) within the 14 days following the follow-up due date (Section 1.3 SoA).
  - If the participant chose **telephone follow-up**: If the participant does not answer the telephone on the day the follow-up questionnaire is due, 3 additional attempts will be made to contact the participant by telephone for a maximum of 9 attempts to contact in 14 days after the follow-up questionnaire due date (Section 1.3 SoA).

Should the participant continue to be unreachable, they will be considered as lost to follow-up.

8 STUDY ASSESSMENTS AND PROCEDURES

8.1 EFFICACY ASSESSMENTS

Primary and secondary endpoints will be determined by the outcome measures. Assessments performed will support the outcome measures listed in Section 3.0 Objectives and Endpoints.

Screening: Participants presenting to the ED of a participating institution will be screened for eligibility using the eligibility criteria listed in sections 5.1 and 5.2 by a trained research team member. The research team member will ask the caregiver questions related to the patient's medical history and current medications that may be contraindicated. Inclusion/exclusion criteria will be determined based on caregiver report of patient's medical history and the details of their current illness. This information will be confirmed with the treating physician to ensure accuracy of reported information and medical oversight. In addition to the patient's medical history, eligibility depends on the treating physician ordering at least 1 dose of ondansetron while the patient is still in the ED, as well as the patient's ED diagnosis. This information will be confirmed using physician or nursing orders and the patient's ED medical chart by the research team member. Permission to access the patient's chart for screening purposes will be obtained from the caregiver/legally authorized individual, in accordance with provincial access to health information legislation.

Enrollment/Baseline Assessments (outcome assessment): A baseline questionnaire will be performed to obtain information supporting the study's outcome measures (e.g. *current medical history, duration and frequency of vomiting, presence of fever, duration and frequency of diarrhea, satisfaction scale, etc.).*

In addition to the questionnaire listed above, the trained research team member will obtain the participant's heart rate (HR) and respiratory rate (RR) at the Enrollment/Baseline visit. The triage temperature and weight will be obtained from the ED medical record. To obtain a baseline understanding of the participant's health and medical condition, a basic review of systems and concomitant medications will also be performed.

Follow-up: Outcome measure end-point data will be obtained using follow-up questionnaires performed 24, 48, and 168 hours (7 days) after the Baseline/Enrollment visit Time 0 (Consent date and time) by research study staff. Questions will be asked related to the following:

- Frequency and duration of vomiting
- Frequency and duration of diarrhea
- Presence of fever
- Return to healthcare provider (resource utilization)
- IV rehydration
- Satisfaction with care and medications provided

Chart Review: The study consent form will contain details regarding the review of medical charts for study purposes. At the baseline/enrollment visit, the ED medical record will be reviewed to collect data points outlined on the Enrollment CRF including, but not limited to; triage date/time, ED disposition, discharge date/time, triage weight, triage temperature, and medications administered during the ED enrollment visit.

A review of the participant's medical chart will also take place between 8 – 30 days after the 168 hour time-point to collect data related to revisits to a health care provider that occurred within 7 days of enrollment for the current illness as defined in Section 3.0. Information regarding type of revisit, treatment provided, discharge diagnoses, and disposition will be collected.

8.2 SAFETY AND OTHER ASSESSMENTS

The following assessments will be performed to ensure the safety of patients as they participate in the study and to assess safety endpoints as described below:

- Frequency and maximal number of diarrheal episodes during the 48 hours following randomization
- Palpitations
- Syncope
- Chest pain

- Arrhythmias
- Serious Adverse Events

Please reference Section 1.3 SoA for a list of all study events/visits**.**

If caregivers report any symptoms of concern to the research team (palpitations, arrhythmias, syncope, etc.) or are concerned about their child's health, they will be advised to follow up with their health care provider or seek immediate medical attention at the nearest hospital. Caregivers will be provided with a list of possible reasons to seek medical care and a disclaimer regarding symptoms of concern and the required action will be included on all follow-up questionnaires.

Screening: A detailed review of the patient's medical history related to the eligibility criteria (e.g. history of prolonged QT syndrome, currently taking apo morphine, etc.) will be performed by the qualified research team member with the caregiver. The information will then be confirmed by the treating physician to comply with medical oversight and ensure patient safety. We will rely on caregiver report of current medications and the medical history of the patient to assess eligibility.

Baseline/Enrollment Visit – Review of Systems: After the patient is deemed eligible and has consented to participate in the study, a review of systems will be performed by the qualified research team member to obtain baseline health information prior to starting the study intervention. The research team member will ask the caregiver/participant questions related to the participant's general health by system (e.g. cardiovascular, respiratory, musculoskeletal, etc.). This allows the research team to more accurately measure any new symptoms or conditions that occur after enrollment in the study. During the review of systems, the research team will also ask the caregiver/participant if they are currently taking any medications or supplements. This information will be recorded on study case report forms. This process will take place after the clinical dose of ondansetron is administered however it must be completed prior to the participant taking the first dose of study medication.

Baseline/Enrollment Visit – Vital Signs: The qualified research team member will obtain the following vitals from the participant prior to study medication administration and/or discharge from the ED (whichever arrives first): Respiratory Rate (RR), Heart Rate (HR), temperature, and weight at triage. All measurements will be obtained from the medical chart however if considered unreliable or if the values are unavailable, research staff will obtain these measurements while the patient is still present in the ED . The procedure for collection of vital signs will be outlined in the TMOP and site specific SOPs.

Hour 24, 48, and 168 Follow-up – Study Intervention Adherence and Administration of Questionnaires: A follow-up questionnaire will be provided to the caregiver via email or telephone (based on their indicated preference). Each questionnaire will ask if the caregiver administered any doses of the study intervention and if administered, will prompt for the most recent date/time of administration. Questionnaires will also provide prompts for feedback regarding the child's illness/condition which will prompt additional questioning related to possible adverse events.

Hour 24, 48 and 168 Follow-up – Assessment of Adverse Events: Obtained from the follow up questionnaire/telephone call completed by trained research staff. Adverse events will be followed until such time as they resolve or are classified as non-progressive to a maximum of 1 month after the start date of the event or until it is apparent that the event will not resolve completely. Trained research staff will follow up with caregivers on a regular basis (daily or weekly, depending on the event details).

Any results obtained from the assessments listed above will not be communicated with the participant or their caregiver unless deemed clinically significant by the site investigator (e.g. Adverse Events reported by the caregiver, abnormal heart rate, etc.).

8.3 ADVERSE EVENTS AND SERIOUS ADVERSE EVENTS

8.3.1 DEFINITION OF ADVERSE EVENTS (AE)

As per the Health Canada Food and Drug Act, an Adverse Event (AE) is defined as: "Any adverse occurrence in the health of a clinical trial subject who is administered a drug that may or may not be caused by the administration of the drug, and includes adverse drug reaction."

An Adverse Drug Reaction (ADR) is defined as "Any noxious and unintended response to a drug that is caused by the administration of any dose of the drug".

8.3.2 DEFINITION OF SERIOUS ADVERSE EVENTS (SAE)

Any adverse occurrence of a clinical trial subject who is administered a drug or placebo that may or may not be caused by the administration of the drug or placebo that results in:

- in-patient hospitalization
- prolongation of existing hospitalization
- that causes a congenital malformation or birth defect
- that results in a persistent or significant disability or incapacity
- is life-threatening
- results in death

An adverse event (AE) or suspected adverse reaction is considered "serious" if, in the view of either the Site Investigator or Sponsor/Principal investigator, it results in any of the following outcomes: death, a life-threatening adverse event, inpatient hospitalization or prolongation of existing hospitalization, a persistent or significant incapacity or substantial disruption of the ability to conduct normal life functions, or a congenital anomaly/birth defect.

Important medical events that may not result in death, be life-threatening, or require hospitalization may be considered serious when, based upon appropriate medical judgment, they may jeopardize the participant and may require medical or surgical intervention to prevent one of the outcomes listed in this definition.

Due to the nature of the illness being studied, some participants may be hospitalized for IV rehydration or ongoing oral rehydration, vomiting, fever, diarrhea, abdominal pain, and/or dehydration. Hospitalization for these reasons will not be reported as a SAE because it is an expected outcome and treatment for this illness.

8.3.3 CLASSIFICATION OF AN ADVERSE EVENT

All adverse events will be classified according to ***MedDRA*** (**Med**ical **D**ictionary for **R**egulatory **A**ctivities) – a multilingual standardized international medical terminology dictionary used for "regulatory communication and evaluation of data pertaining to medicinal products for human use."

8.3.3.1 SEVERITY OF EVENT

Adverse Events will be assessed for severity by the site investigator using the following definitions of severity:

- **Mild:** Events require minimal or no treatment and do not interfere with the participant's daily activities.
- **Moderate:** Events result in a low level of inconvenience or concern with the therapeutic measures. Moderate events may cause some interference with functioning.
- **Severe:** Events interrupt a participant's usual daily activity and may require systemic drug therapy or other treatment. Severe events are usually potentially life-threatening or incapacitating. *Note:* The term "severe" does not necessarily equate to "serious".

8.3.3.2 RELATIONSHIP TO STUDY INTERVENTION

All adverse events (AEs) must have their relationship to study intervention assessed by the site investigator/clinician who examines and evaluates the participant based on temporal relationship and his/her clinical judgment. The degree of certainty about causality will be graded using the categories below. In a clinical trial, the study product must always be suspect.

- **Definitely Related** – There is clear evidence to suggest a causal relationship, and other possible contributing factors can be ruled out. The clinical event, including an abnormal laboratory test result, occurs in a plausible time relationship to study intervention administration and cannot be explained by concurrent disease or other drugs or chemicals. The response to withdrawal of the study intervention (dechallenge) should be clinically plausible. The event must be pharmacologically or phenomenologically definitive, with the use of a satisfactory rechallenge procedure if necessary.
- **Probably Related** – There is evidence to suggest a causal relationship, and the influence of other factors is unlikely. The clinical event, including an abnormal laboratory test result, occurs within a reasonable time after administration of the study intervention, is unlikely to be attributed to concurrent disease or other drugs or chemicals, and follows a clinically reasonable response on withdrawal (dechallenge). Rechallenge information is not required to fulfill this definition.
- **Potentially Related** – There is some evidence to suggest a causal relationship (e.g., the event occurred within a reasonable time after administration of the trial medication). However, other factors may have contributed to the event (e.g., the participant's clinical condition, other concomitant events). Although an AE may rate only as "possibly related" soon after discovery, it can be flagged as requiring more information and later be upgraded to "probably related" or "definitely related", as appropriate.
- **Unlikely to be related** – A clinical event, including an abnormal laboratory test result, whose temporal relationship to study intervention administration makes a causal relationship improbable (e.g., the event did not occur within a reasonable time after administration of the study intervention) and in which other drugs or chemicals or underlying disease provides plausible explanations (e.g., the participant's clinical condition, other concomitant treatments).
- **Not Related** – The AE is completely independent of study intervention administration, and evidence exists that the event is definitely related to another etiology. There must be an alternative, definitive etiology documented by the clinician.

8.3.3.3 EXPECTEDNESS

The site investigator or co-investigator will be responsible for determining whether an adverse event (AE) is expected or unexpected. An AE will be considered unexpected if the nature, severity, or frequency of the event is not consistent with the risk information previously described for the study intervention.

The most common adverse events reported related to the use of ondansetron oral solution are: headache, constipation, and sensation of flushing. These adverse events will be considered expected.

8.3.4 TIME PERIOD AND FREQUENCY FOR EVENT ASSESSMENT AND FOLLOW-UP

The occurrence of an adverse event (AE) or serious adverse event (SAE) may come to the attention of study personnel during the follow-up questionnaires performed with caregivers or if a study participant presents for medical care to the enrolling ED, or upon review of data by a study monitor.

All AEs including local and systemic reactions not meeting the criteria for SAEs will be captured on the appropriate case report form (CRF). Information to be collected includes an event description, date and time of onset, clinician's assessment of severity, relationship to study product (assessed only by those with the training and authority to make a diagnosis), and date and time of resolution/stabilization of the event.

Any medical condition that is present at the time the participant is screened will be considered as baseline and not reported as an AE. However, if the study participant's condition deteriorates at any time during the study, it will be recorded as an AE.

Changes in the severity of an AE will be documented to allow an assessment of the duration of the event at each level of severity to be performed. AEs characterized as intermittent require documentation of onset and duration of each episode.

Study staff and the site or co-investigator will record all reportable events with start dates occurring any time after informed consent is obtained until 7 (for non-serious AEs) or 15 days (for SAEs) after the last day of study participation.

During each follow up contact made, the investigator/study staff will inquire about the occurrence of AE/SAEs since the last contact (via telephone or email). Events will be followed for outcome information until resolution or stabilization.

As listed in section 1.3 Schedule of Activities, Adverse Events will be assessed via telephone or email contact with the caregiver 24, 48, and 168 hours after enrollment in the study. Possible Adverse Events will be assessed using open-ended feedback questions on the follow-up questionnaires. Trained research study staff will review the response provided by the caregiver and will follow up by telephone as soon as possible. Details regarding the event will be obtained via telephone and contact will be made daily, or weekly, as required to obtain the resolution/stabilization details.

8.3.5 ADVERSE EVENT REPORTING

All adverse events (AEs) will be reported to the participating site's responsible Research Ethics Board (REB) in accordance with the participating site's AE reporting guidelines (e.g. *CAREB Guidance on Reporting Unanticipated Problems including Adverse Events to Research Ethics Boards*). The site or co-investigator will assess each adverse event in terms of its expectedness and relationship to the study drug. Information to be collected will include an event description, date of onset, investigator/clinician's assessment of severity, relationship to study product

(assessed by the site investigator), and date of resolution/stabilization of the event and event outcome (resolved/recovered, recovered with sequelae, not recovered/not resolved, death, or unknown).

The following Disease Related Events (DREs) will not be reported per the standard reporting process described above.

- Hospitalization for IV rehydration OR gastroenteritis like symptoms
- Future health care provider visit, ED return visit for vomiting, diarrhea, or dehydration
- IV rehydration
- Nausea
- Loss of appetite
- Abdominal pain, distension, bloating
- Vomiting, diarrhea, fever, flatulence
- Rash or Diaper Rash
- Any event that was present at enrollment during the review of systems and that has not worsened (i.e. not new since enrollment in the study).

The events listed above are part of the natural history of the underlying disease process of acute gastroenteritis in children and will be recorded on study CRFs or in note-to-files, as applicable.

Adverse Event reporting procedures can be found in the study TMOP.

8.3.6 SERIOUS ADVERSE EVENT REPORTING

Any AE considered to be serious by the Principal Investigator or Site Investigator or which meets the definition of an SAE included in section 8.3.2 must be reported to the lead site/Principal Investigator and the study Data Safety and Monitoring Board (DSMB), in accordance with the DSMB charter reporting requirements.

The site investigator/clinician will immediately report to the principal investigator/sponsor any serious adverse drug reaction or adverse event, whether or not considered study intervention related, including those listed in the protocol or product monograph and must include an assessment of whether there is a reasonable possibility that the study intervention caused the event. Study endpoints that are serious adverse events (e.g. all-cause mortality) must be reported in accordance with the protocol unless there is evidence suggesting a causal relationship between the study intervention and the event (e.g. death from anaphylaxis). In that case, the site investigator must immediately report the event to the principal investigator/sponsor.

Reports of Serious Adverse Events and/or Serious Adverse Drug Reactions must be reported to the lead site within 48 hours of the site-investigator becoming aware of the event. Event details should be captured on the appropriate study CRF.

The principal investigator/sponsor is required to inform Health Canada of any serious, unexpected adverse drug reaction (ADR) that has occurred inside or outside Canada. ADR reports must be filed:

- where the ADR is neither fatal nor life-threatening, within 15 days after becoming aware of the information
- where it is fatal or life-threatening, immediately where possible and, in any event, within 7 days after becoming aware of the information
- within 8 days after having informed Health Canada of the ADR, submit as complete as possible, a report which includes an assessment of the importance and implication of any finding

Where the adverse drug reaction is neither fatal nor life-threatening, the sponsor will notify Health Canada and all participating investigators of the event and any potential serious risks, from clinical trials or any other source, as soon as possible, but in no case later than 15 calendar days after the sponsor determines that the information qualifies for reporting.

Each ADR which is subject to expedited reporting should be reported individually in accordance with the Health Canada / ICH Guidance Document E2A: Clinical Safety Data Management: Definitions and Standards for Expedited Reporting.

A completed ADR Expedited Reporting Summary Form should be attached to the front of the completed ADR report – suggested report format: Suspect Adverse Reaction Report - CIOMS form of the Council for International Organizations of Medical Sciences. The report should be submitted by fax or email to the appropriate regulatory directorate.

All serious adverse events (SAEs) will be followed until satisfactory resolution or until the site investigator deems the event to be chronic or the participant is stable. Other supporting documentation of the event may be requested by the lead site/Principal Investigator and should be provided as soon as possible.

SAEs will be reported to the participating and lead site's responsible REB in accordance with the institutional REB Serious Adverse Event reporting guidelines (e.g. *CAREB Guidance on Reporting Unanticipated Problems including Adverse Events to Research Ethics Boards*):

- If the SAE is considered life threatening or fatal, a report must be submitted within 7 calendar days of the investigator becoming aware of the event.
- For all other SAEs, a report must be submitted within 15 calendar days of the investigator becoming aware of the event(s).

8.3.7 REPORTING EVENTS TO PARTICIPANTS

All study participants, or their caregivers, will be notified if at any time new findings become available which may be in the best medical interest of the study participant or impact their willingness to continue participation in the study.

8.3.8 EVENTS OF SPECIAL INTEREST

Not applicable.

8.3.9 REPORTING OF PREGNANCY

The possibility of pregnancy occurring in this population while the participant is on protocol and taking the study intervention is exceedingly unlikely given the age range of the participants and their illness status. Given the routine use of ondansetron in pregnant women, and the duration of study medication administration, pregnancy screening is not indicated. In fact, ondansetron use in the US in 2014 had reached 22% with the greatest amount of use occurring in the first trimester.[38]

Nevertheless, if by remote chance the participant tests positive on a pregnancy test and notifies the study team, the site investigator will be notified immediately. The site investigator will then make an informed decision

regarding the continuation of the study medication. The site investigator may contact the medical monitor for additional support, as applicable. Pregnancy tests will not be administered by the study team and therefore it will be up to the participant/caregiver to notify the study team of a positive pregnancy test.

The pregnancy will be the responsibility of the participant and their treating physician, not the study team. Safety information and a study information sheet is provided to the participant and their caregiver at time of enrollment; which should be shared with the participant's treating physician by the participant/caregiver.

Pregnancy will not be reported as an Adverse Event. Reports of pregnancy will be sent to local Research Ethics Boards, as dictated by local institutional policy.

8.4 UNANTICIPATED PROBLEMS

8.4.1 DEFINITION OF UNANTICIPATED PROBLEMS (UP)

Unanticipated problems are problems involving risks to participants or others to include, in general, any incident, experience, or outcome that meets **all** of the following criteria:

- Unexpected regarding nature, severity, or frequency given (a) the research procedures that are described in the protocol-related documents, such as the Research Ethics Board (REB) approved research protocol and informed consent document; and (b) the characteristics of the participant population being studied;
- Related or possibly related to participation in the research ("possibly related" means there is a reasonable possibility that the incident, experience, or outcome may have been caused by the procedures involved in the research); and
- Suggests that the research places participants or others at a greater risk of harm (including physical, psychological, economic, or social harm) than was previously known or recognized.

8.4.2 UNANTICIPATED PROBLEM REPORTING

The investigator will report unanticipated problems (UPs) to the reviewing REB and the Data Coordinating Center (WCHRI-DCC), principal investigator (PI), and Network Coodinator (NC). The UP report will include the following information:

- Protocol identifying information: protocol title and number, PI's name, and the REB project number;
- A detailed description of the event, incident, experience, or outcome;
- An explanation of the basis for determining that the event, incident, experience, or outcome represent a UP;
- A description of any changes to the protocol or other corrective actions that have been taken or are proposed in response to the UP.

To satisfy the requirement for prompt reporting, UPs will be reported using the following timeline:

- UPs that are serious adverse events (SAEs) will be reported to the REB, and the WCHRI- DCC/study sponsor within 7 days of the site-investigator becoming aware of the event.
- Any other UP will be reported to the REB and the WCHRI-DCC/study sponsor within 15 days of the site investigator becoming aware of the problem.
- All UPs should be reported to appropriate institutional officials (as required by an institution's written reporting procedures).

Additional information regarding reporting procedures (signatories, format, etc.) can be found in the study TMOP and SOP documentation.

8.4.3 REPORTING UNANTICIPATED PROBLEMS TO PARTICIPANTS

All study participants/caregivers will be notified of any new Unanticipated Problems (UP) which may affect the participant's current or future willingness to participate in the study.

Participants/caregivers will be notified individually by telephone or email, based on their chosen follow-up preference and the study team's ability to confirm the individual has received the information.

9 STATISTICAL CONSIDERATIONS

9.1 STATISTICAL HYPOTHESES

The primary objective is to determine if the use of multi-dose oral ondansetron compared with placebo, provided at ED discharge, will result in a reduction in the proportion of children with moderate to severe disease following discharge as reflected by a MVS score $\geq$9.

9.2 SAMPLE SIZE DETERMINATION

The primary analysis will be performed on a binary outcome: Development of moderate-to-severe disease. The power of this analysis is based on the proportion of patients with moderate-to-severe disease. Our pilot data indicate that 30% of patients will have moderate to severe disease following the index visit (personal communication with Dr. Jianling Xie). Furthermore, expert surveys indicated that an absolute risk reduction of 10% would constitute a minimal clinically-important difference (MCID). Therefore, our sample size calculation assumed a 30% event rate in the control group for which we desire to detect an absolute beneficial treatment effect of 10% with 90% power. Using a two-sided type I error of 0.05 and the hypothesized proportions yields a required total sample size of 784 patients. Our expected power, should we find different event rates in our 2 groups, is displayed in Table 2.0.

Table 2.0 – Expected Power

Outcome Control	Outcome Intervention	% Difference	Power
0.25	0.15	0.10	0.94
0.25	0.2	0.05	0.39
0.25	0.25	0.0	NA
0.3	0.15	0.15	0.999
0.3	0.25	0.05	0.35
0.35	0.15	0.2	~1
0.35	0.2	0.15	0.997
0.35	0.25	0.1	0.87

Based on previous work by our group[32], we assume a 10% loss to follow up (784/0.90 = 870), 5% drop out, and 3% drop in (caregivers who are provided with ondansetron outside the study protocol) rate (870/(0.92)2 = 1030. Thus, the total number randomized (final sample size) will be 1030.

9.3 POPULATIONS FOR ANALYSES

Screening Population: The screening population includes all patients who are screened for eligibility in to the trial, regardless of randomization into the trial or treatment status. This population represents all patients who meet inclusion criteria outlined in the study protocol and who are screened in real-time by study staff at the participating site. This population will be used for reporting of study flow per CONSORT guidelines.

Intention-To-Treat Population: The Intention-to-Treat (ITT) population includes all participants who are randomized into the trial, regardless of adherence to the protocol, including, for example, participants who receive no study drug. The ITT population will be used for the primary efficacy analyses in the study, as well as for main efficacy analyses of secondary outcomes. All analyses using the ITT population will be based on each participant's assigned treatment arm, regardless of treatment actually received.

Safety Analysis Population: The safety analysis population will include a subset of participants who took any doses of the study medication. A sub-analysis will be performed including only those who took 3 or more doses to assess the impact of multiple doses of ondansetron on diarrhea (and other safety outcomes). Reporting of results will be summarized according to treatment received. This population will be used for analysis of adverse events to examine safety outcomes.

9.4 STATISTICAL ANALYSES

9.4.1 GENERAL APPROACH

For the primary outcome, the significance level will be set at 0.05. The overall significance level for statistical tests on the secondary outcomes will be set at 0.05.

To control the overall significance level for the secondary outcomes, the significance level will be adjusted for multiple comparisons. Vomiting and diarrhea duration, vomiting and diarrhea frequency, proportion with vomiting, unscheduled healthcare provider visits, intravenous rehydration, and caregiver satisfaction will be subject to Holm's stepdown procedure. Specifically, the smallest of the eight p-values will be compared to a significance level of 0.05/8. If significance is reached, the next-smallest p-value will be compared to 0.05/7, and so on. The final p-value of the six will be compared to 0.05, assuming that all others are significant. Confirmatory and tertiary outcome results will not be adjusted for multiple comparisons, as these are more exploratory in nature.

A 95% confidence interval will be reported around estimates of the treatment effects of the primary and secondary outcomes.

All analytical models will be evaluated and adjustments will be made if any assumptions appear to be seriously violated.

Publication of the primary results will include reporting of key baseline characteristics overall, and by assigned treatment arm (active or placebo). These will include, but will not be limited to,

- Gender
- Age
- Season
- Weight

- Primary care physician access
- Daycare attendance
- Receipt of rotavirus vaccine
- Vomiting and diarrhea duration and frequency
- Presence of fever
- Prior ED visits, IV rehydration, hospitalization
- Baseline MVS
- Duration of symptoms prior to enrollment (<48 hours vs. 48 hours or more)
- Antibiotic usage during the 14 days prior to ED visit
- Hydration assessment (Clinical Dehydration Scale score)
- Need for hospitalization from initial ED visit

9.4.2 ANALYSIS OF THE PRIMARY EFFICACY ENDPOINT(S)

The **primary outcome** for the study is a **post-enrollment (168 hours (7 days)) Modified Vesikari Score (MVS),** defined as an MVS ≥ 9. The post-enrollment score is calculated based on the elements of the score collected Day 0 through Day 7. Each of the seven components will be assigned a score for the entire study period (from ED disposition to Day 7). Scoring of each component is described in Table 3.0.

Table 3.0 Modified Vesikari Scale

Scale Component	Score			
	0 Points	1 Point	2 Points	3 Points
Diarrhea duration (hours)	0	< 96.0	96.0 – < 120.0	≥ 120.0
Maximum number of watery stools/24 hour period	0	1–3	4–5	≥6
Vomiting duration (hours)	0	< 24.0	24.0 – < 48.0	≥ 48.0
Maximum number of vomiting episodes/24 hour period	0	1	2–4	≥5
Maximum recorded rectal temperature (°C)†	<37.0	37.0 – < 38.5	38.5 – < 39.0	≥39.0
Unscheduled health care visit	None	NA	Primary care	Emergency department
Treatment	None	Rehydration with intravenous fluids	Hospitalization	NA

†Temperatures are adjusted for the location of measurement: 1.1°C was added to axillary temperatures and 0.6°C was added to oral temperatures[39]

The components will be calculated by the following rules:

1. **Maximum number of episodes of vomiting in a 24-hour period:** The number of episodes (defined as per the protocol) of vomiting will be collected in 24-hour blocks.

2. **Maximum number of episodes of diarrhea (watery stools) in a 24-hour period:** The number of episodes (defined as per the protocol) of diarrhea will be collected in 24-hour blocks.

3. **Maximum recorded rectal temperature:** The maximal temperature will be collected in 24-hour blocks. The method of measurement will be collected (e.g. temporal, rectal, oral) and the temperature obtained will be adjusted to a rectal temperate as per Table 3.0.

4. **Vomiting duration:** This is calculated as the time between ED disposition and the last vomiting episode. When vomiting is reported to have stopped on study questionnaires, and started again on a later report, but prior to all symptoms (i.e. vomiting and diarrhea) stopping, the vomiting is considered to have continued throughout that time-period. The vomiting episode would thus be calculated from ED disposition date/time until the final vomiting stop date/time. If there is a period of 24 hours without vomiting and without diarrhea, then the episode would be considered to have ended and symptoms reported after that period would not be included in the calculation.

5. **Diarrhea duration:** This component will be calculated using exactly the same procedure described for vomiting when diarrhea is present at randomization. If diarrhea is not present at randomization and never occurs, diarrhea duration will be 0. If diarrhea starts after randomization but prior to a 24 hour period without vomiting, the start time will be calculated from the reported start time and the end time will be computed as described for vomiting duration. Specifically, if there is a period of 24 hours without vomiting and without diarrhea then the episode would be considered to have ended and symptoms reported after that period would not be included in the calculation.

6. **Unscheduled healthcare visit:** Presence of an unscheduled healthcare visit will be collected on the questionnaire and will be classified as "none", "primary care", or "emergency department". All revisits to the participating site are verified by database review.

7. **Treatment:** Treatment will be classified as "none", "intravenous rehydration", or "hospital admission". If the participant is admitted directly following their enrollment ED visit and that admission stay is longer than 48 hours, it will be counted as treatment in the post enrollment MVS. If the participant's enrollment admission stay was less than 48 hours, it will not be counted as "treatment".

Data needed to calculate the MVS will be collected at Enrollment (Day 0), on the 24, 48, and 168 hour questionnaires, and verified using chart review performed within 37 days of participant enrollment.
The primary outcome will be tabulated by treatment for each site and weight stratum. A Mantel-Haenszel statistic, assessing the effect of treatment on the primary outcome controlling for strata, will be calculated to test the primary hypothesis. Summary statistics for the effect sizes will be given. Choice of final summary statistic reported (e.g. risk difference, risk ratio, or odds ratio) will be based, in part, by which statistic is least variable across subgroups (i.e. shows the smallest degree of interaction across subgroups). It is expected that the asymptotic version of the Mantel-Haenszel test will be appropriate for the final analysis. In the case of low outcome counts, the Mantel-Fleiss criterion will be assessed. If the criterion has a value less than 5, the exact version of the Mantel-Haenszel test will be used and the two-sided p-value will be defined as the sum of all probabilities of test statistics with point probabilities less than or equal to that of the observed value. In the event any patients were randomized within an incorrect stratum due to misspecification at time of randomization, the actual, rather than assigned category will be used in the above analysis.

Although randomization should result in approximate balance between treatment arms with respect to critical baseline variables affecting the outcomes, additional exploratory analyses will be conducted to assess the effects of treatment after adjusting for baseline covariates in models for the primary outcome.

The covariates considered are:
- Baseline MVS (actual score)
- Duration of symptoms prior to enrollment (<48 hours vs. 48 hours or more)
- Age
- Gender
- Clinical center

- Antibiotic usage during the 14 days prior to ED visit
- Severity of baseline diarrhea and vomiting
- Hydration assessment
- Need for hospitalization at index visit

9.4.3 ANALYSIS OF THE SECONDARY ENDPOINT(S)

Secondary outcomes and how they will be assessed can be found below:

1. **Frequency of vomiting from study enrollment to day 7:** The total number of episodes of vomiting and diarrhea related to the presenting illness will be compared between groups (active and placebo). This data will be obtained from the study questionnaires performed on Day 0, 24, 48, and 168 (7 days) hours after enrollment. Questionnaires (telephone or email) will contain questions regarding vomiting presence, number of episodes, and vomiting cessation date and time. Frequency will be defined as the total number of episodes across the study duration and will not consider differences in the vomiting duration across participants.
2. **Duration of vomiting:** The total number of days the participant experiences vomiting from Day 0 to Day 7. This data will be obtained from the study questionnaires performed on Day 0, 24, 48, and 168 (7 days) hours after enrollment. Questionnaires (telephone or email) will contain questions regarding vomiting presence, number of episodes, and vomiting cessation date and time. A participant with a 24-hour break in symptoms will be assumed to have ceased vomiting.
3. **Proportion of participants who experience vomiting within 7 days of enrollment:** The proportion of participants will be determined using data from the participant questionnaires performed 24, 48, and 168 hours after enrollment (Time 0).
4. **Proportion of participants who require an unscheduled health care provider visit within 7 days of enrollment:** Return visits for unscheduled care to a health care provider related to vomiting, diarrhea, dehydration, fever, abdominal pain, or fluid refusal within 7 days, not including scheduled visits (e.g. reassessments, vaccinations, etc.) will also be measured using follow up questionnaires 24, 48, and 168 hours after enrollment. Return to care data will be confirmed by reviewing the participant's hospital chart. Not all data within a patient's medical record will be available to the research team therefore the questionnaires will be the primary source of this data.
5. **Proportion of participants who require intravenous rehydration:** If the participant required an unscheduled health care visit (see #4 above), the participant will be asked additional questions related to intravenous (IV) insertion, treatment course, and disposition. Intravenous fluids administered at the index visit will not be included as part of this outcome.
6. **Caregiver satisfaction as measured by a 5-point Likert scale in the 48 hours following ED disposition:** Caregiver satisfaction with the therapy provided (oral ondansetron or placebo) in the 48 hours following ED disposition. This will be measured using a 5 point Likert scale on the questionnaire administered 168 hours after Time 0.

The analysis of secondary outcomes will be undertaken as detailed below:

1. **Frequency of vomiting from study enrollment to Day 7:** The total number of episodes of vomiting and diarrhea related to the presenting illness will be compared between groups (active and placebo) using a generalized linear model to compare differences between Poisson rates whilst adjusting for stratification.

2. **Duration of vomiting :** The total number of days the participant experiences vomiting from Day 0 to Day 7 will be compared using the Van Elteren test, stratified by center and duration of symptoms (pre-enrollment). If a number of patients still experience symptoms at the 7-day follow-up questionnaire, this will be tested using a suitable procedure to take account of censoring such as median regression or additive hazard models. Additional analyses involving this outcome will include linear regression models that adjust for possible effects of baseline characteristics.
3. **Proportion of participants who experience vomiting within 7 days of enrollment:** The proportion of participants with vomiting following enrollment will be compared using Mantel-Haenszel test stratified by site and weight.
4. **Proportion of participants who require an unscheduled health care provider visit within 7 days of enrollment:** The proportion of participants who require an unscheduled health care provider visit within 7 days of enrollment will be compared using Mantel-Haenszel test stratified by site and weight.
5. **Proportion of participants who receive intravenous rehydration:** The proportion of participants who receive intravenous rehydration will be compared using Mantel-Haenszel test stratified by site and weight. If the number of participants requiring intravenous rehydration is low then exact calculations without stratification may be required.
6. **Caregiver satisfaction as measured by a 5-point Likert scale in the 48 hours following ED disposition:** Caregiver satisfaction with the therapy provided (oral ondansetron or placebo) in the 48 hours following ED disposition will be compared using a Mann-Whitney U Test.

9.4.4 SAFETY ANALYSES

Secondary safety outcomes will be obtained via participant follow-up questionnaires performed 24, 48, and 168 hours (7 days) after enrollment (Time 0 – consent date and time).

Summaries of incidence rates (frequencies and percentages), intensity, and relationship to study of individual AEs by System Organ Class and Preferred Term (MedDRA) will be prepared. All AEs beginning after randomization but before discharge will be included. Basic summaries by assigned groups will be prepared. The DSMB may request to see more detailed tables. The occurrence of any adverse event will be considered as a dichotomous outcome.

To obtain SAE, palpitation, pre-syncope/syncope, chest pain, and arrhythmia data; each questionnaire will contain open ended, non-symptom specific questions where caregivers can provide the study team with information about their child's illness or any other concern they may have. Any response to this question will result in a trained study team member discussing the information provided in more detail via telephone. Any symptom or condition that meets the definition of an SAE will be categorized as such and reported appropriately.

1. **Frequency of diarrheal episodes during the 48-hours following ED disposition:** Frequency and maximal number of diarrheal episodes will be obtained by asked about presence of diarrhea on each questionnaire the caregiver completes. If diarrhea is present, further questions related to the number of episodes will be asked. Frequency will be calculated over 48 hours for all patients regardless of whether they have greater than 24 hours with no symptoms.
2. **Maximal number of diarrheal episodes in 24 hours:** Same as above – Frequency
3. **Serious Adverse Events (SAEs)**
4. **Palpitations**
5. **Pre-syncope /syncope**
6. **Chest Pain**

7. **Arrhythmias**

Serious Adverse Events/Deaths will be reported separately in a similar fashion to the more general AE reports. In addition, narratives will be available for each event.

The analysis of safety outcomes will be undertaken as detailed below:

1. **Frequency of diarrheal episodes during the 48-hours following ED disposition:** The total number of episodes of diarrhea related to the presenting illness will be compared between groups (active and placebo) using a generalized linear model to compare differences between Poisson rates whilst adjusting for stratification.
2. **Maximal number of diarrheal episodes in 24 hours:** To test whether this outcome differs between the active and placebo treatments, we will use a Mann-Whitney U-test.
3. **Serious Adverse Events (SAEs):** The frequency of SAEs will be reported for both active and placebo groups. No formal testing will be undertaken.
4. **Palpitations:** The frequency of palpitations will be reported for both active and placebo groups. No formal testing will be undertaken.
5. **Pre-syncope /syncope:** The frequency of pre-syncope/syncope will be reported for both active and placebo groups. No formal testing will be undertaken.
6. **Chest Pain:** The frequency of chest pain will be reported for both active and placebo groups. No formal testing will be undertaken.
7. **Arrhythmias:** The frequency of arrhythmias will be reported for both active and placebo groups. No formal testing will be undertaken

9.4.5 BASELINE DESCRIPTIVE STATISTICS

Baseline characteristics will be compared between groups using frequency counts and percentages for discrete variables, and means, medians, standard deviations, and interquartile ranges for continuous variables.

9.4.6 PLANNED INTERIM ANALYSES

As per the DSMB charter, no interim analysis will be performed for the DOSE-AGE study. In the case that an interim analysis is to take place, it will be done in accordance with the DSMB charter. We will also make the appropriate adjustments to the significance level and update the study Statistical Analysis Plan.

Early stopping for efficacy and futility will not be considered due to concerns for overestimating or underestimating treatment effects. The DSMB will, in collaboration with the trial steering committee, establish safety stopping rules prior to trial initiation.

Data center biostatisticians involved in this study will be partially blinded, aware of results by treatment arm, but not of arm identities. Other data center personnel will be blinded to treatment assignments, however the data center study team for this trial will have access to individual safety and efficacy data. Personnel at the clinical centers will be blinded to aggregate safety and efficacy data until the time of final analysis or until the decision is made to unblind all investigators to study results.

All by-treatment interim analyses will refer to arms as "A" and "B" throughout the report presented to the DSMB. The DSMB will have the option of being unblinded to treatment arm identity at any time.

For additional information about safety stopping rules, reference the DSMB charter.

9.4.7 SUB-GROUP ANALYSES

The following pre-specified subgroup analyses of the primary outcome will be performed:

1. Sex
2. Age: 6 months to 3 years; 3 to 6 years; 6 to 10 years; >10 years
3. Vomiting frequency: >10 episodes in preceding 24 hours
4. Presence of diarrhea (yes/no) in preceding 24 hours

A "subgroup" effect will be declared to be significant only if the interaction between assigned treatment and the subgroup factor is significant in the appropriate statistical model testing for each particular interaction, at a significance level of 0.05/4 = 0.0125. These results must still be viewed with caution given the number of subgroup analyses. As the primary outcome is binary, logistic regression models will be used with a main effect for treatment, a main effect for the subgroup variable of interest, and an interaction between the subgroup variable of interest and the treatment. We will also consider stratifying by site and weight in subgroup analyses.

9.4.8 TABULATION OF INDIVIDUAL PARTICIPANT DATA

In the analyses planned, no individual participant data will be listed.

9.4.9 EXPLORATORY ANALYSES

The final analysis for the comparison of oral ondansetron to placebo will take place in one stage. The main report of the trial will be prepared after every participant has completed the protocol and all data has been collected, cleaned, and the database has been "closed".

Any post hoc, exploratory analyses completed to support planned study analyses, which were not identified in the study SAP, will be documented and reported as such in all study publications.

10 SUPPORTING DOCUMENTATION AND OPERATIONAL CONSIDERATIONS

10.1 REGULATORY, ETHICAL, AND STUDY OVERSIGHT CONSIDERATIONS

10.1.1 INFORMED CONSENT PROCESS

10.1.1.1 CONSENT/ASSENT AND OTHER INFORMATIONAL DOCUMENTS PROVIDED TO PARTICIPANTS

Consent forms describing in detail the study intervention, study procedures, and risks are given to the caregiver or legally authorized representative (LAR) for the participant, and all participants beyond age of maturity. The consent process will be completed by a trained study team member. Written documentation of informed consent is required before collecting any data (with the exception of data related to eligibility determination), performing study procedures, or randomization to the study intervention.

In accordance with TCPS2: Tri Council Policy statement regarding consent of minors, consent from mature minors will be sought where applicable. The age of consent varies by province however if the age of consent has been reached, consent will be obtained from the participant directly, provided the research team deems the individual to have decision making capacity (section 10.1.1.2). Due to the large amount of communication and symptom tracking required, caregivers of mature minors will also be required to provide informed consent for completion of the study questionnaires, where mature minor consent is applicable.

Assent forms describing in detail the study intervention, study procedures, and risks will be provided to participants who are the age of assent. The age of assent will be defined by local REB policy, as outlined by ICH-GCP E11 'Clinical Investigations of Medicinal Products in the Pediatric Population' (*e.g. the University of Calgary REB requires assent be obtained from children age 7 years and older*).

Written documentation of informed consent is required before randomizing to/administering study intervention. Assent reading level will be determined based on REB guidelines at each participating study site (*e.g. University of Calgary REB Assent Requirements: children aged 7-11 years = reading level of grade 2; children 12 years and older = reading level of grade 4*)

All caregiver and mature minor informed consent forms should be written at a grade 6-8 reading level.

The trained research team member will review the applicable consent/assent forms with the caregiver/LAR and participant and provide them with a copy for independent review. The consent process will be documented by the research team member obtaining consent.

10.1.1.2 CONSENT / ASSENT PROCEDURES AND DOCUMENTATION

Informed consent/assent is a process that is initiated before the participant and their caregiver/LAR agree to participate in the study and continues throughout the individual's study participation, as required. All consent and assent forms will be Research Ethics Board (REB) approved. The participant and their caregiver/LAR will be asked to read and review the document prior to agreeing to participate in the study. Trained study staff will explain the research study to the participant and their caregiver and answer any questions that may arise. A verbal explanation will be provided in terms suited to the participant's and their caregivers' comprehension of the purposes, procedures, and potential risks of the study and their rights as research participants. Participants and their caregivers will have the opportunity to carefully review the written consent and assent forms free from undue influence and ask questions before signing. The participants and their caregivers will have the opportunity to review and discuss the study before agreeing to participate.

The caregiver or mature minor will sign the informed consent form and (if applicable) the participant will sign the assent document before any study procedures or intervention are performed. Participants and their caregivers must be informed that participation is voluntary and that they may withdraw from the study at any time, without prejudice. A signed copy of the informed consent and assent (if applicable) documentation will be given to the participant for their records.

Every effort will be made to provide caregivers/LARs and participants who do not speak English and/or French with a translator in their language of choice to assist with the process of informed consent. This individual may be the caregiver of the child or another family member. If translation is required, a narrative summary will be written by the research staff member to detail who translated the information and to document the informed consent process.

Capacity to consent to a research study must be assessed prior to obtaining informed consent from the caregiver/LAR and/or the participant. Capacity to consent will be assessed and documented by the research study team member according to site-specific procedures for assessing capacity.

The informed consent process will be conducted and documented in the patient medical chart and REDCap (including the date), and the form(s) signed before the participant undergoes any study-specific procedures. The rights and welfare of the participants will be protected by emphasizing to them that the quality of their medical care will not be adversely affected if they decline to participate in this study.

For a review of the full informed consent process, reference the TMOP and site specific SOPs for Informed Consent.

10.1.2 STUDY DISCONTINUATION AND CLOSURE

This study may be temporarily suspended or prematurely terminated if there is sufficient reasonable cause. Written notification, documenting the reason for study suspension or termination, will be provided by the suspending or terminating party to study participants, principal investigator, funding agency, the sponsor, REB and Health Canada, as applicable.

If the study is prematurely terminated or suspended, the Principal Investigator (PI) will promptly inform Site Investigators. Site Investigators will then inform study participants and the Research Ethics Board (REB) of record the reason(s) for termination or suspension. Study participants will be contacted, as applicable.

Circumstances that may warrant termination or suspension include, but are not limited to:

- Determination of unexpected, significant, or unacceptable risk to participants
- Demonstration of efficacy that would warrant stopping
- Insufficient compliance with protocol requirements
- Data that are not sufficiently complete and evaluable
- Determination that the primary endpoint has been met
- Determination of futility

The study may resume once concerns about safety, protocol compliance, and data quality are addressed, and satisfy the sponsor, REB and/or Health Canada, as applicable.

10.1.3 CONFIDENTIALITY AND PRIVACY

Participant confidentiality and privacy are strictly held in trust by the participating investigators, their staff, and the sponsor(s). This confidentiality is extended to cover the clinical information relating to participants. Therefore, the study protocol, documentation, data, and all other information generated will be held in strict confidence. No information concerning the study or the data will be released to any unauthorized third party without the prior written approval of the sponsor.

All research activities will be conducted in as private a setting as possible

The study monitor, other authorized representatives of the sponsor, representatives of the Research Ethics Board (REB), or regulatory agencies may inspect all documents and records required to be maintained by the investigator, including but not limited to, medical records (office, clinic, or hospital) and pharmacy records for the participants in this study. The clinical study site will permit access to such records.

The study participant's contact information will be securely stored at each clinical site for internal use during the study. At the end of the study, all records will continue to be kept in a secure location for as long a period as dictated by the reviewing REB, institutional policies, or sponsor requirements.

Study participant research data, which is for purposes of statistical analysis and scientific reporting, will be stored in the REDCap electronic data capture system at the Women and Children's Health Research Institute (WCHRI) Data Coordinating Centre (DCC) at the University of Alberta. This data will not generally include the participant's contact or identifying information. Rather, individual participants and their research data will be identified by a unique study identification number. Permission to store data at the WCHRI DCC will be included in the informed consent.

WCHRI's REDCap installation is housed in a secure data centre at the University of Alberta Hospital and is behind the Faculty of Medicine & Dentistry's firewall. Data is entered through a web-based interface using 128 bit SSL encryption. Login is via a username/password pair with additional two factor authentication (2FA). Additional information is available in WCHRI's privacy document (*https://redcap.ualberta.ca/privacy.pdf*).

Coordinators and investigators at each study site will only have access to data relating to their own study participants. Study management staff (PI and project manager) at the lead site will have access to data for all study participants. Staff at the WCHRI DCC will have access to data for all study participants. This access is required to perform system management functions, data cleaning, and analysis. Once the study database is "locked" and data has been extracted for analysis, read only access to the study database will be granted to the PI and/or their designate if requested.

Participant contact information will be stored in the REDCap data capture system for the purposes of follow-up contact via email. The participants sex, date of birth, weight, and date of ED visit will also be stored in the REDCap database for the purposes of return visit validation, chart reviews, accurate age, and study medication dose calculations. Participant/caregivers will be informed of data collection and storage policies during the informed consent process.

10.1.4 FUTURE USE OF STORED SPECIMENS AND DATA

Data will remain in the REDCap system at the WCHRI DCC until all data management and statistical analysis activity has been completed. Following study completion and publication the data will be deleted from the REDCap system.

The WCHRI DCC will facilitate the deposit of de-identified data in a publicly accessible, secure and curated repository for discovery and reuse by others in accordance with the Tri Agency Statement of Principles on Digital Data Management (2018).

The PI will be responsible for storing copies of the data and other study materials in a secure archival facility in compliance with Health Canada and local institutional research data retention policy. At the end of this retention period these materials will be destroyed.

See also Section 10.1.3, Confidentiality and Privacy, and Section 10.1.9, Data Handling and Record Keeping, for further information on the future use of study records.

10.1.5 KEY ROLES AND STUDY GOVERNANCE

Below is a list of the key individuals involved in the study:

TRIAL PRINCIPAL INVESTIGATOR	***PROGRAM PRINCIPAL INVESTIGATOR***	***TRIAL MEDICAL MONITOR***	***TRIAL PROJECT MANAGER***
Stephen Freedman, MDCM MSc	Terry Klassen, MD MSc	Terry Klassen, MS, MSc	Sarah Williamson-Urquhart, BScKIN CCRP
Alberta Children's Hospital<br>The University of Calgary	Children's Hospital Research Institute of Manitoba (CHRIM)	Children's Hospital Research Institute of Manitoba (CHRIM)	Alberta Children's Hospital<br>The University of Calgary
C4-634<br>28 Oki Drive NW<br>Calgary, AB T3B 6A8	JBRC 513<br>715 McDermot Ave<br>Winnipeg, MB R3E 3P4	JBRC 513<br>715 McDermot Ave<br>Winnipeg, MB R3E 3P4	C4-633-03<br>28 Oki Drive NW<br>Calgary, AB T3B 6A8
403-955-7740 /<br>403-955-7873 (admin)	204-789-3754 /<br>204-977-5605 (admin)	204-789-3754 /<br>204-977-5605 (admin)	403-955-2482
stephen.freedman@ahs.ca /<br>tanya.borthwick@ahs.ca (admin)	*tklassen@chrim.ca* /<br>*jveinot@chrim.ca* (admin)	*tklassen@chrim.ca* /<br>*jveinot@chrim.ca* (admin)	*sarah.urquhart@ahs.ca*

The trial project manager (PM, co-located with the trial principal investigator) as delegated by the Principal Investigator will manage the day-to-day operations of this trial. They will report to the NM (network manager) and Principal Investigator and will liaise continually with coordinators at the six clinical research sites to promote information sharing, coordination, and to monitor progress.

Project managers will be responsible for scheduled reporting to the NCC, study Steering Committee, and Data Safety and Monitoring Board as dictated by the NCC and Principal Investigator.

Each recruiting institution will name a minimum of two site investigators (the site lead investigator and site co-investigator) and at least one individual to be the site coordinator. Site investigators will be held responsible for the conduct of the study at their site in accordance with tri-council, and ICH-GCP policies. The site coordinator will be responsible for day-to-day operations of the trial at their respective sites, as well as communication and reporting to the site investigator, project manager, and principal investigator.

RECRUITING SITE LOCATION	***SITE INVESTIGATOR***	***SITE CO-INVESTIGATOR***
**Alberta Children's Hospital (lead site)**<br>*Calgary, Alberta*	Dr. Stephen Freedman	Dr. Antonia Stang
**Centre Hospitalier Universitaire Sainte Justine**<br>*Montréal, Quebec*	Dr. Serge Gouin	Dr. Jocelyn Gravel
**Children's Hospital of Eastern Ontario**<br>*Ottawa, Ontario*	Dr. Amy Plint	Dr. Maala Bhatt
**Children's Hospital of Winnipeg**<br>*Winnipeg, Manitoba*	Dr. Darcy Beer	Dr. Scott Sawyer
**London Health Sciences Centre**<br>*London, Ontario*	Dr. Gary Joubert	Dr. Naveen Poonai

**Stollery Children's Hospital**<br>*Edmonton, Alberta*	Dr. Andrew Dixon	Dr. Samina Ali

Expanded role details for all study collaborators and staff (Principal Investigator, Co-investigator, Project Manager, Site Coordinator, Research Nurse, etc.) can be found in the TMOP, along with institutional contact details.

10.1.6 SAFETY OVERSIGHT

Safety oversight will be under the direction of a Data and Safety Monitoring Board (DSMB) composed of individuals with the appropriate expertise, including: Pediatric Emergency Medicine, trial methodology, and biostatistics. Members of the DSMB should be independent from study conduct and free of conflict of interest, or measures should be in place to minimize perceived conflict of interest. The DMSB will operate under the rules of an approved charter which will outline all terms of reference, as well as the frequency of meetings, that will be reviewed at the organizational meeting of the DSMB. At this time, each data element that the DSMB needs to assess for this study will be defined. The DSMB will provide its input to the Program PI, the Network Coordinator (NC), the Study Principal Investigator, the Project Manager, and when applicable to the responsible REB.

The Data Safety Monitoring Board (DSMB) membership, responsibilities, and administrative duties can be found in the DSMB approved Charter. A list of DSMB member names and contact information can be found in the TMOP.

10.1.7 CLINICAL MONITORING

Monitoring for this study will be performed by the University of Alberta's Quality Management in Clinical Research office (QMCR) (*https://www.ualberta.ca/quality-management-in-clinical-research*). Details of clinical site monitoring will be documented and conducted in accordance with a Clinical Monitoring Plan (CMP). The CMP will describe in detail who will conduct the monitoring, at what frequency monitoring will be done, at what level of detail monitoring will be performed, and the distribution of monitoring reports.

Clinical site monitoring is conducted to ensure that the rights and well-being of trial participants are protected, that the reported trial data are accurate, complete, and verifiable, and that the conduct of the trial is in compliance with the currently approved protocol/amendment(s), with International Conference on Harmonization Good Clinical Practice (ICH GCP), Tri-Council Policy Statement 2 (TCPS2) and with applicable regulatory requirement(s) (e.g. Health Canada).

Monitoring of data integrity, regulatory compliance and participant safety will include centralized review of CRFs and other study documents for compliance, data accuracy and completeness. During the study, monitoring will include remote and on-site monitoring visits for source data verification, review of the investigator's site file and drug handling records. QMCR will be given direct access to source documents, CRFs and other study-related documents. By signing the informed consent form, the caregiver or legally authorized representative (LAR) gives authorized QMCR staff direct access to the participant's medical records and the study data. This study may be subject to audit or inspection by representatives of QMCR or any participating institution.

All site investigator(s) and institution(s) will permit trial-related monitoring, audits, REB review and regulatory inspection(s) by providing direct access to source data/documentation, as required.

Noncompliance with the protocol, SOPs, GCP, and/or applicable regulatory requirement(s) by a site investigator/institution, or by a member of the Sponsor-Investigator staff will lead to prompt action by the Sponsor-Investigator to secure compliance.

If noncompliance that significantly affects or has the potential to significantly affect human subject protection or reliability of trial results is discovered, the Sponsor-Investigator will perform a root cause analysis and implement appropriate corrective and preventive actions.

Should the monitoring and/or audit identify serious and/or persistent noncompliance on the part of a site investigator/institution, the Sponsor-investigator will terminate the investigator's/institution's participation in the trial. When an investigator's/institution's participation is terminated because of noncompliance, the Sponsor-Investigator will notify promptly the regulatory authorities, as applicable.

The Sponsor-investigator will be responsible for implementing and maintaining quality assurance and quality control systems with written SOPs to ensure that trials are conducted and data are generated, documented (recorded), and reported in compliance with the protocol, GCP, and the applicable regulatory requirements. The Sponsor-Investigator will be responsible for securing agreements from all involved parties to ensure direct access to all trial related sites, source data/documents, and reports for the purpose of monitoring and auditing by the sponsor, and inspection by regulatory authorities.

All research personnel will be required to complete the Tri-Council Policy Tutorial (version 2.0): Ethical Conduct for Research Involving Humans, the Good Clinical Practices course, and the Division 5 Health Canada training module. Completion will be documented prior to implementation of the study. Privacy and confidentiality policy and procedure will also be reviewed at the study recruitment training session for all study personnel.

For more information regarding monitoring, including scheduled time points, and contact information for monitoring agents please reference the TMOP.

10.1.8 QUALITY ASSURANCE AND QUALITY CONTROL

Quality control (QC) procedures will be implemented beginning with the data entry system and data QC checks that will be run on the database will be generated. Any missing data or data anomalies will be communicated to the site(s) for clarification/resolution.

Each clinical site will perform internal quality management of study conduct, data collection, documentation and completion. An individualized quality management plan will be developed to describe each site's quality management.

Following written Standard Operating Procedures (SOPs), the monitors will verify that the clinical trial is conducted and data are generated, documented (recorded), and reported in compliance with the protocol, International Conference on Harmonisation Good Clinical Practice (ICH GCP), and applicable regulatory requirements (e.g. Good Manufacturing Practices (GMP)).

The investigational site will provide direct access to all trial-related source data/documents, and reports for the purpose of monitoring and auditing by the sponsor, and inspection by local and regulatory authorities. Regular monitoring will be performed in accordance with ICH-GCP; see section ***10.1.7 Clinical Monitoring*** for more information on monitoring procedures and schedule.

Each clinical site will perform internal quality management of study conduct, data collection, documentation and completion. An individualized quality management plan will be developed to describe each site's quality management. Site specific policies and procedures must be documented in site specific standard operating procedure (SOP) documentation.

As stated in section 10.1.7, all research personnel will be required to complete the Tri-Council Policy Tutorial: Ethical Conduct for Research Involving Humans (TCPS2) or equivalent, a Good Clinical Practice (GCP) course, and the Division 5 Health Canada training module. Each participating site may have additional research training requirements for study personnel. Additional training should be outlined in site training SOPs and documented locally.

All sites will receive one in-person training session prior to the initiation of the trial. Training of site staff will occur at the in-person training session. Site investigators, along with the site coordinator will be responsible for training any staff who are unable to attend the in-person training session. Additional training resources can be made available by the lead site (e.g. webinars, presentations). Should there be study staff turnover, the site investigator and coordinator will be responsible for study specific training with new staff members. Site provided training must be documented and proof of training must be sent to the principal investigator and project manager at the lead site.

Study specific training requirements are outlined in the TMOP.

10.1.9 DATA HANDLING AND RECORD KEEPING

10.1.9.1 DATA COLLECTION AND MANAGEMENT RESPONSIBILITIES

Data collection and entry is the responsibility of the clinical trial staff at the site under the supervision of the site investigator. The investigator will be responsible for ensuring the accuracy, completeness, legibility, and timeliness of the data reported. All source documents will be completed in a neat, legible manner to ensure accurate interpretation of data. Paper records (e.g. copies of consent and assent forms) will be stored exclusively in a secure, locked location with limited access.

Data management services will be provided by the WCHRI-DCC. Data will be entered into a validated electronic, web based, data capture system (REDCap) and will be managed according to approved data management and quality plans. During the data collection process data may be collected on paper and transcribed into the study database or, in some cases, information obtained from the participants may be entered directly into the study database. Under these circumstances the study database may be considered to be an electronic source document. Selected data elements will be validated electronically on an ongoing basis throughout the study and any discrepancies will be assigned to members of the study team for resolution.

The study team will provide hardcopies of the study worksheets for use as source documentation for recording data for participants enrolled in the study if REDCap or internet access is unavailable. Hardcopy data will be entered into REDCap as soon as the online systems are available. Data derived from hardcopies should be consistent with the data recorded on the source documents.

Clinical data (including adverse events (AEs), concomitant medications, and expected adverse reactions data) and clinical laboratory data will be entered into REDCap. REDCap includes password protection and internal quality checks, such as automatic range checks, to identify data that appear inconsistent, incomplete, or inaccurate. Clinical data will be entered directly from the source documents, participants, or study team.

10.1.9.2 STUDY RECORDS RETENTION

The site investigator will be responsible for retaining (archiving) their own essential study documents that individually or collectively permit the evaluation and conduct of the study and the quality of data, in accordance

with ICH-GCP and applicable regulatory requirements. All study documents, including source, are to be stored in a confidential location with secured and limited access. Paper data (e.g. copies of consent and assent forms) should be stored in the participating site investigator's research space in a locked, secure location. All electronic records and data sets will be encrypted and password protected with access only permitted by the PIs, project manager, site coordinators, and research team members. Results will not be reported in a way that identifies any individuals.

All study related documentation will be retained in accordance with Health Canada's Food and Drug Regulations Part C, Division 5 (C.05.012) for 25 years and per the investigational site's institutional record management and retention policies. No records will be destroyed without the written consent of the Principal Investigator and/or Sponsor. It is the responsibility of the Principal Investigator to inform the Participating Site Investigator when these documents no longer need to be retained.

In addition, and in accordance with section C.05.013 of the regulations, information shall be made available within 2 days if there is a concern regarding the use of the drug for the purposes of a clinical trial, including a risk to health of the subjects involved in that trial. In other cases, records shall be provided within 7 days of a request.

10.1.10 PROTOCOL DEVIATIONS

A protocol deviation is any noncompliance with the clinical trial protocol as contained in this document, International Conference on Harmonization Good Clinical Practice (ICH-GCP), or Trial Manual of Procedures (TMOP) requirements. The noncompliance may be either on the part of the participant, the investigator, or the study site staff. As a result of deviations, corrective actions are to be developed by the site and implemented promptly.

These practices are consistent with ICH-GCP sections:

- 4.5 Compliance with Protocol, sub-sections 4.5.1, 4.5.2, and 4.5.3
- 5.1 Quality Assurance and Quality Control, sub-section 5.1.1
- 5.20 Noncompliance, sub-sections 5.20.1, and 5.20.2.

It is the responsibility of the site investigator to use continuous vigilance to identify and report protocol deviations to the principal investigator/sponsor. Deviations related to the safety of individuals participating in the study should be reported within 7 calendar days of identification of the deviation to the principal investigator/sponsor. All other protocol deviations must be reported to the principal investigator/sponsor within 15 calendar days of the scheduled protocol-required activity.

All deviations must be addressed in study source documents and reported to the reviewing Research Ethics Board (REB) per their protocol deviation reporting policies. The site investigator is responsible for knowing and adhering to the reviewing REB requirements.

Any unanticipated deviation(s) from the original statistical plan will be described and justified in the published protocol and/or in the final report, depending on when a decision is made to change the approach

Further details regarding handling of protocol deviations are outlined in the study TMOP.

10.1.11 PUBLICATION AND DATA SHARING POLICY

This study will comply with the CIHR Open Access Policy. This trial will be registered at ClinicalTrials.gov, and results information from this trial will also be submitted to ClinicalTrials.gov. Every attempt will be made to

publish the study protocol and the results in peer-reviewed journals. Data from this study may be requested from other researchers by contacting the principal investigator.

Conduct, reporting, editing, and publication of resultant scholarly work will be guided by the International Committee of Journal Medical Editors (ICJME)'s published recommendations.

Publication guidelines will be developed by the National Coordinating Centre and study principal investigator, in consultation with site investigators, the network manager, and the study steering committee and will be listed in the applicable study agreements, SOPs, and TMOP.

The identity of participants will not be revealed in any published data or presentation and only de-identified datasets will be shared with approved collaborators.

10.1.12 CONFLICT OF INTEREST POLICY

The independence of this study from any actual or perceived influence, such as by the pharmaceutical industry, is critical. Therefore, any actual conflict of interest of persons who have a role in the design, conduct, analysis, publication, or any aspect of this trial will be disclosed and managed appropriately. Furthermore, persons who have a perceived conflict of interest will be required to have such conflicts managed in a way that is appropriate to their participation in the design and conduct of this trial. The study leadership in conjunction with the CIHR has established policies and procedures for all study group members to disclose all conflicts of interest and will establish a mechanism for the management of all reported dualities of interest.

10.2 ADDITIONAL CONSIDERATIONS

Not applicable.

10.3 ABBREVIATIONS

ADR	Adverse Drug Reaction
AE	Adverse Event
AGE	Acute Gastroenteritis
ANCOVA	Analysis of Covariance
CAREB	Canadian Association of Research Ethics Boards
CFR	Code of Federal Regulations
CIHR	Canadian Institutes for Health Research
CIOMS	The Council for International Organizations of Medical Sciences
CMP	Clinical Monitoring Plan
CONSORT	Consolidated Standards of Reporting Trials
CRA	Clinical Research Associate (Monitor)
CRF	Case Report Form
CTA	Clinical Trial Application
DCC	Data Coordinating Center
DCF	Data Collection Form
DSMB	Data Safety Monitoring Board
DRE	Disease-Related Event
ED	Emergency Department
eCRF	Electronic Case Report Forms
GCP	Good Clinical Practice
GLP	Good Laboratory Practices
GMP	Good Manufacturing Practices
HC	Health Canada
ICH	International Conference on Harmonization
ISM	Independent Safety Monitor
ISO	International Organization for Standardization
ITT	Intention-To-Treat
LAR	Legally Authorized Representative
LSMEANS	Least-squares Means
MAOI	Monoamine Oxidase Inhibitor
MedDRA	Medical Dictionary for Regulatory Activities
MVS	Modified Vesikari Scale Score
TMOP	Manual of Procedures
NCC	Network Coordinating Centre
NC	Network Coordinator
NCT	National Clinical Trial
NOL	No Objection Letter
PI	Principal Investigator
PM	Project Manager
QA	Quality Assurance
QC	Quality Control
REB	Research Ethics Board
SAE	Serious Adverse Event
SAP	Statistical Analysis Plan
SMC	Safety Monitoring Committee
SNRI	Serotonin-Norepinephrine Reuptake Inhibitor
SoA	Schedule of Activities
SOC	System Organ Class
SOP	Standard Operating Procedure
SPOR	Strategy for Patient-Oriented Research

SSRI	Selective Serotonin Reuptake Inhibitor
UP	Unanticipated Problem
US	'United States' of America
WCHRI	Women and Children's Health Research Institute

10.4 PROTOCOL AMENDMENT HISTORY

The table below is intended to capture changes of REB-approved versions of the protocol, including a description of the change and rationale. A Summary of Changes table for the current amendment is located in the Protocol Title Page.

Version/Date	Description of Change	Brief Rationale
03 February 2019	-	Initial REB Approved Version (board of record – UofC)
14 March 2019	Minor edits as described in the summary of changes table	Sterile bottles not needed for preparation of Intervention.
02 March 2020	Addition of exclusion criteria. Addition of Nausea and Loss of Appetite as DREs. Minor edits as described in the summary of changes table. Updated MVS scale score. Removal of Appendix C – discharge materials.	Safety and consistency. Added nausea and loss of appetite as DREs because these are common presenting complaints prior to enrollment and consistent with the disease process. Updated MVS for consistency across SAP, and protocol manuscript. Documents in Appendix C provided separately (not necessary to attach to protocol).
25 November 2022	Addition of a generic oral ondansetron product to be used in the absence of availability of Zofran® and inclusion of the generic product monograph and matching placebo formulation in study protocol appendices.	Due to intermittent supply issues with the Novartis Zofran® product, it was determined that the generic product could be used as a substitution, along with an updated matching placebo formulation. This allows the study to continue recruitment as there are no ingredient differences between the generic and name brand product.

11 REFERENCES

1. Scallan E, Griffin PM, Angulo FJ, Tauxe RV, Hoekstra RM. Foodborne illness acquired in the United States--unspecified agents. Emerging infectious diseases 2011;17:16-22.

2. Ozuah PO, Avner JR, Stein RE. Oral rehydration, emergency physicians, and practice parameters: a national survey. Pediatrics 2002;109:259-61.

3. Freedman SB, Willan AR, Boutis K, Schuh S. Effect of Dilute Apple Juice and Preferred Fluids vs Electrolyte Maintenance Solution on Treatment Failure Among Children With Mild Gastroenteritis: A Randomized Clinical Trial. JAMA 2016;315:1966-74.

4. Freedman SB, Adler M, Seshadri R, Powell EC. Oral ondansetron for gastroenteritis in a pediatric emergency department. N Engl J Med 2006;354:1698-705.

5. Freedman SB, Pasichnyk D, Black KJ, et al. Gastroenteritis Therapies in Developed Countries: Systematic Review and Meta-Analysis. PLoS One 2015;10:e0128754.

6. Freedman SB, Steiner MJ, Chan KJ. Oral ondansetron administration in emergency departments to children with gastroenteritis: an economic analysis. PLoS Med 2010;7.

7. Gray JM, Maewal JD, Lunos SA, Furnival RA, Hendrickson MA. Ondansetron Prescription for Home Use in a Pediatric Emergency Department. Pediatr Emerg Care 2017.

8. Eltorki M. Re: M Xu, M Reider. A supplementary home dose of oral ondansetron given in anticipation of recurrent emesis in paediatric acute gastroenteritis. Paediatr Child Health 19(2):107-108. Paediatrics & child health 2014;19:500 -

9. Xu M, Rieder M. A supplementary home dose of oral ondansetron given in anticipation of recurrent emesis in paediatric acute gastroenteritis. Paediatrics & child health 2014;19:107-8.

10. Freedman SB, Powell EC, Nava-Ocampo AA, Finkelstein Y. Ondansetron dosing in pediatric gastroenteritis: a prospective cohort, dose-response study. Paediatr Drugs 2010;12:405-10.

11. Nunez J, Liu DR, Nager AL. Dehydration treatment practices among pediatrics-trained and non-pediatrics trained emergency physicians. Pediatr Emerg Care 2012;28:322-8.

12. Freedman SB, Ali S, Oleszczuk M, Gouin S, Hartling L. Treatment of acute gastroenteritis in children: an overview of systematic reviews of interventions commonly used in developed countries. Evid Based Child Health 2013;8:1123-37.

13. Rerksuppaphol S, Rerksuppaphol L. Randomized study of ondansetron versus domperidone in the treatment of children with acute gastroenteritis. J Clin Med Res 2013;5:460-6.

14. Kharbanda AB, Hall M, Shah SS, et al. Variation in resource utilization across a national sample of pediatric emergency departments. J Pediatr 2013;163:230-6.

15. Freedman SB, Hall M, Shah SS, et al. Impact of increasing ondansetron use on clinical outcomes in children with gastroenteritis. JAMA Pediatr 2014;168:321-9.

16. Keren R. Ondansetron for acute gastroenteritis: a failure of knowledge translation. JAMA Pediatr 2014;168:308-9.

17. Mullarkey C, Crowley E, Martin C. The addition of ondansetron to a oral rehydration protocol for children with acute gastroenteritis. Ir Med J 2013;106:266-8.

18. Kwon KT, Rudkin SE, Langdorf MI. Antiemetic use in pediatric gastroenteritis: a national survey of emergency physicians, pediatricians, and pediatric emergency physicians. Clin Pediatr (Phila) 2002;41:641-52.

19. Elliott EJ. Acute gastroenteritis in children. BMJ 2007;334:35-40.

20. Haines E, van Amerongen R, Birkhahn R, Wen W, Gaeta T. Evaluating outcomes associated with a discharge action plan employing single-dose home use of ondansetron in patients with acute gastroenteritis. Open Access Emerg Med 2012;4:1-4.

21. Sturm JJ, Hirsh DA, Schweickert A, Massey R, Simon HK. Ondansetron use in the pediatric emergency department and effects on hospitalization and return rates: are we masking alternative diagnoses? Annals of emergency medicine 2010;55:415-22.

22. Ramsook C, Sahagun-Carreon I, Kozinetz CA, Moro-Sutherland D. A randomized clinical trial comparing oral ondansetron with placebo in children with vomiting from acute gastroenteritis. Ann Emerg Med 2002;39:397-403.

23. Yilmaz HL, Yildizdas RD, Sertdemir Y. Clinical trial: oral ondansetron for reducing vomiting secondary to acute gastroenteritis in children--a double-blind randomized study. Aliment Pharmacol Ther 2010;31:82-91.

24. Freedman SB, Thull-Freedman JD, Rumantir M, Atenafu EG, Stephens D. Emergency department revisits in children with gastroenteritis. J Pediatr Gastroenterol Nutr 2013;57:612-8.

25. Freedman SB, Uleryk E, Rumantir M, Finkelstein Y. Ondansetron and the risk of cardiac arrhythmias: a systematic review and postmarketing analysis. Ann Emerg Med 2014;64:19-25.e6.

26. Moeller JR, Gummin DD, Nelson TJ, Drendel AL, Shah BK, Berger S. Risk of Ventricular Arrhythmias and Association with Ondansetron. J Pediatr 2016;179:118-23 e1.

27. ZOFRAN® Tablets, Oral Solution and Injection [package insert]. Novartis Pharmaceuticals Canada Inc. Dorval, QC. 2016.

28. Tomasik E, Ziolkowska E, Kolodziej M, Szajewska H. Systematic review with meta-analysis: ondansetron for vomiting in children with acute gastroenteritis. Alimentary pharmacology & therapeutics 2016;44:438-46.

29. Freedman SB, Eltorky M, Gorelick M, Pediatric Emergency Research Canada Gastroenteritis Study G. Evaluation of a gastroenteritis severity score for use in outpatient settings. Pediatrics 2010;125:e1278-85.

30. Schnadower D, Tarr PI, Gorelick MH, et al. Validation of the modified Vesikari score in children with gastroenteritis in 5 US emergency departments. J Pediatr Gastroenterol Nutr 2013;57:514-9.

31. Schnadower D, Tarr PI, Casper TC, et al. Lactobacillus rhamnosus GG versus Placebo for Acute Gastroenteritis in Children. N Engl J Med 2018;379:2002-14.

32. Freedman SB, Williamson-Urquhart S, Farion KJ, et al. Multicenter Trial of a Combination Probiotic for Children with Gastroenteritis. N Engl J Med 2018;379:2015-26.

33. Freedman SB, Powell E, Seshadri R. Predictors of outcomes in pediatric enteritis: a prospective cohort study. Pediatrics 2009;123:e9-16.

34. Coffin SE, Elser J, Marchant C, et al. Impact of acute rotavirus gastroenteritis on pediatric outpatient practices in the United States. Pediatr Infect Dis J 2006;25:584-9.

35. Freedman SB, Tung C, Cho D, Rumantir M, Chan KJ. Time-series analysis of ondansetron use in pediatric gastroenteritis. J Pediatr Gastroenterol Nutr 2012;54:381-6.

36. Hoffman RJ, Alansari K. Effect of intravenous ondansetron on QTc interval in children with gastroenteritis. Am J Emerg Med 2017.

37. Millan MJ, Maiofiss L, Cussac D, Audinot V, Boutin JA, Newman-Tancredi A. Differential actions of antiparkinson agents at multiple classes of monoaminergic receptor. I. A multivariate analysis of the binding profiles of 14 drugs at 21 native and cloned human receptor subtypes. J Pharmacol Exp Ther 2002;303:791-804.

38. Taylor LG, Bird ST, Sahin L, et al. Antiemetic use among pregnant women in the United States: the escalating use of ondansetron. Pharmacoepidemiol Drug Saf 2017;26:592-6.

39. Alpern ER, Henretig FM. Fever. In: Fleisher GR, Ludwig S, eds. Textbook of Pediatric Emergency Medicine. 6th ed. Philadelphia, PA: Lippincott Williams & Wilkins; 2010:266-75.

12 APPENDICES

See appendices as labeled.

Appendix	Description
Appendix A	Zofran® Product Monograph
Appendix B	Ondansetron Oral Solution (JAMP Pharmaceuticals) Product Monograph
Appendix C	Placebo Comparator Formulation – Zofran® (Novartis Pharmaceuticals)
Appendix D	Placebo Comparator Formulation – Ondansetron (JAMP Pharmaceuticals)
