# Supplementary material for: Persistent Vomiting Among Children With Acute Gastroenteritis: A Secondary Analysis of a Randomized Clinical Trial
Source: JAMA Netw Open. 2026 May 6;9(5):e2610898. doi: 10.1001/jamanetworkopen.2026.10898 (PMC13150644; doi:10.1001/jamanetworkopen.2026.10898)
Supplement: Supplement 2. — eAppendix. Model Derivation eFigure 1. Receiver Operating Characteristic Curve of the Derived Score to Correctly Classify the Occurrence of ≥3 Episodes of Vomiting 24 hours Following ED Discharge eFigure 2. Predicted and Observed Probabilities of (A) ≥3 Vomiting Episodes During the 24-Hours Following the Index Emergency Department Visit and (B) Unscheduled Health Care Visit in 7 Days After Index Emergency Department Discharge eFigure 3. Receiver Operating Characteristic Curve of the Derived Score to Correctly Classify the Occurrence of (A) Unscheduled Healthcare Revisit, (B) Intravenous Fluid Administration, and (C) Hospitalization Within 7-Days of Index ED Discharge eTable 1. Results of Mixed-Effect Logistic Regression Model for Each Outcome, With the Derived Score as the Independent Variable eTable 2. Effect Estimates for Occurrence of ≥3 Episodes of Vomiting in the 24-Hours Following Emergency Department Discharge Evaluated Using Complete Case and Multiple Imputation Datasets Analyzed Using Mixed-Effects Logistic Regression, Linear Probability Models With Robust Standard Errors, and LASSO-Selected Models eReferences. [file jamanetwopen-e2610898-s002.pdf]

Supplementary Online Content

Sumner M, Xie J, Williamson-Urquhart S, et al. Persistent vomiting among children with acute gastroenteritis: a secondary analysis of a randomized clinical trial. *JAMA Netw Open.* 2026;9(5):e2610898. doi:10.1001/jamanetworkopen.2026.10898

eAppendix. Model Derivation

eFigure 1. Receiver Operating Characteristic Curve of the Derived Score to Correctly Classify the Occurrence of ≥3 Episodes of Vomiting 24 hours Following ED Discharge

eFigure 2. Predicted and Observed Probabilities of (A) ≥3 Vomiting Episodes During the 24-Hours Following the Index Emergency Department Visit and (B) Unscheduled Health Care Visit in 7 Days After Index Emergency Department Discharge

eFigure 3. Receiver Operating Characteristic Curve of the Derived Score to Correctly Classify the Occurrence of (A) Unscheduled Healthcare Revisit, (B) Intravenous Fluid Administration, and (C) Hospitalization Within 7-Days of Index ED Discharge

eTable 1. Results of Mixed-Effect Logistic Regression Model for Each Outcome, With the Derived Score as the Independent Variable

eTable 2. Effect Estimates for Occurrence of ≥3 Episodes of Vomiting in the 24-Hours Following Emergency Department Discharge Evaluated Using Complete Case and Multiple Imputation Datasets Analyzed Using Mixed-Effects Logistic Regression, Linear Probability Models With Robust Standard Errors, and LASSO-Selected Models

eReferences.

This supplementary material has been provided by the authors to give readers additional information about their work.

© 2026 Sumner M et al. *JAMA Network Open.*

eAppendix. Model Derivation

Model tuning was performed using 5-fold cross-validation stratified by study site to preserve representation across folds. The penalty parameter was optimized according to the "1 standard error" (1 SE) rule selecting the most parsimonious model within 1 SE of the minimum cross-validated log-loss.[1] During exploratory cross-validation, the unconstrained 1 SE solution selected zero predictors, reflecting potential underfitting. To promote clinical interpretability, we imposed a minimum constraint of four non-zero predictors. The final penalty parameter selected had the largest value satisfying both the 1 SE rule and the minimum predictor constraints, favoring parsimony while maintaining acceptable discrimination. To assess model robustness, we compared the mean cross-validated log-loss between the unconstrained (0 predictors) and constrained (≥4 predictors) solutions. Across imputations, the constrained models achieve slightly lower mean log-loss (difference: -0.0095) with an average of 5 predictors, indicating improved or equivalent predictive performance despite increase model complexity. Predictors with non-zero coefficients in ≥5 of the 10 imputed models were retained as stable variables.[2] To enhance interpretability, predictors with mean absolute log-odds ratios <0.10 were assigned a score of 0. Although we adjusted for the effects of ondansetron administration within 24 hours of ED discharge, it was not assigned a score as it does not have clinical meaning for a predictive score applied at initial ED presentation. For the remaining predictor variables, mean log-odds ratios across the selected models were computed, divided by 0.10, and rounded to the nearest integer to create the final point-based score.[3] Total scores were calculated by summing the assigned point values.

© 2026 Sumner M et al. *JAMA Network Open.*

eFigure 1. Receiver Operating Characteristic Curve of the Derived Score to Correctly Classify the Occurrence of ≥3 Episodes of Vomiting 24 hours Following ED Discharge

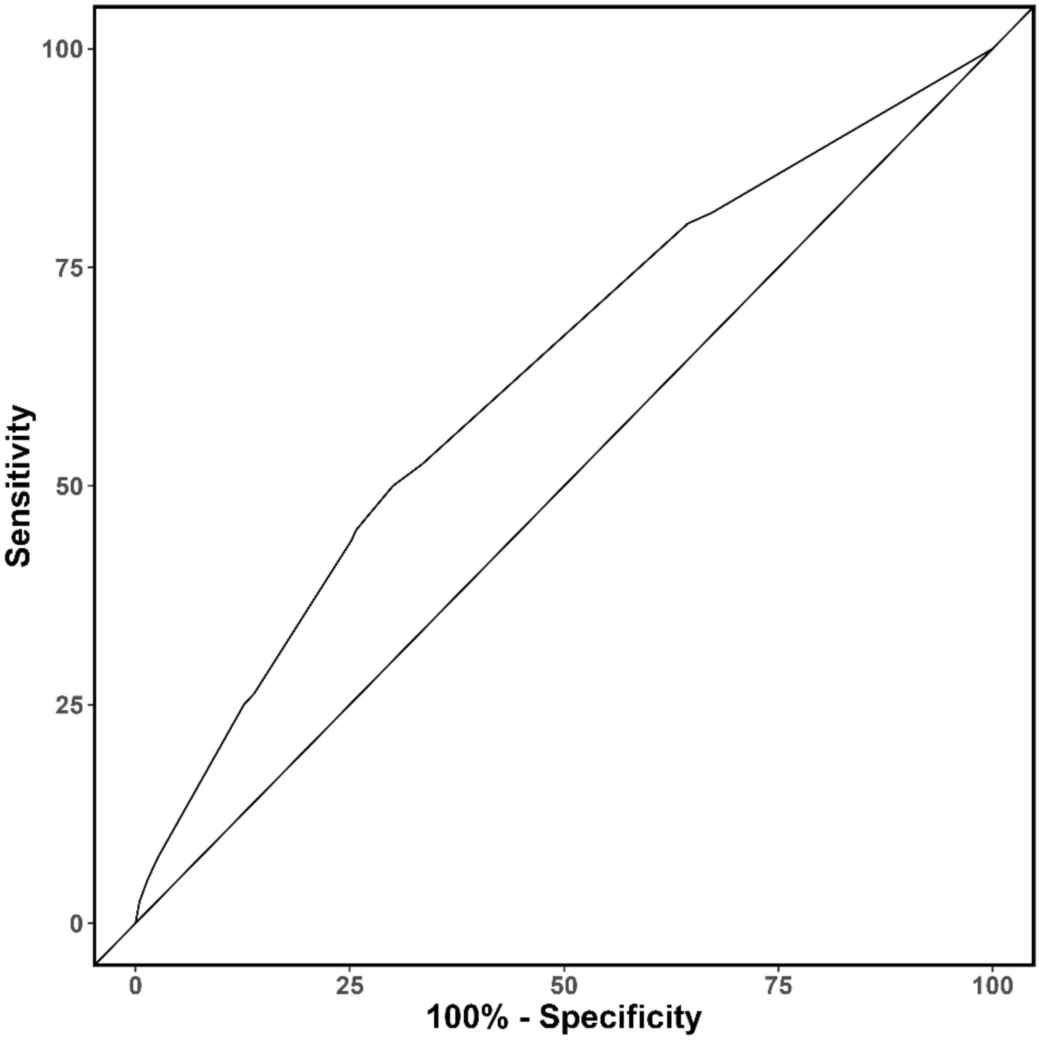

- Area under the curve (AUC) of the score to classify the occurrence of ≥3 episodes of ongoing vomiting in the 24 hours after index emergency department visit: AUC = 0.63 (95%CI: 0.56, 0.69)
- Mean AUC from 1000 bootstrap samples: 0.63 (95%CI: 0.57, 0.69)
- Standard deviation of AUC from 1000 bootstrap samples: 0.03
- Standard error of AUC from 1000 bootstrap samples: 0.001
- Using Youden Index we identified that a score ≥3 maximizes overall correct classification with a sensitivity= 0.50 (95%CI 0.39, 0.61), specificity = 0.70 (95%CI 0.67, 0.73).

© 2026 Sumner M et al. *JAMA Network Open.*

eFigure 2. Predicted and Observed Probabilities of (A) ≥3 Vomiting Episodes During the 24-Hours Following the Index Emergency Department Visit and (B) Unscheduled Health Care Visit in 7 Days After Index Emergency Department Discharge

A) ≥3 vomiting episodes

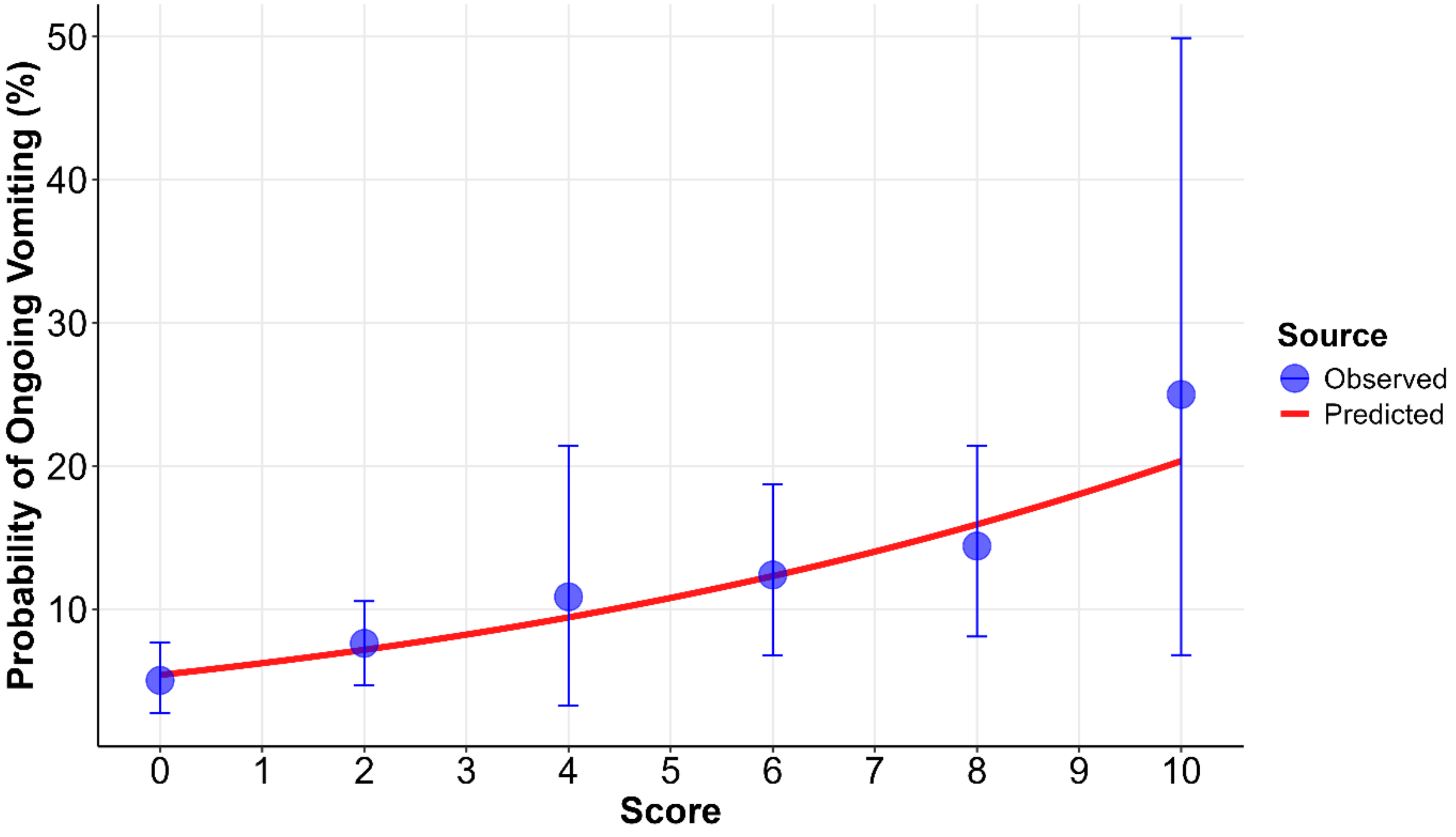

© 2026 Sumner M et al. *JAMA Network Open.*

B) Unscheduled health care visit

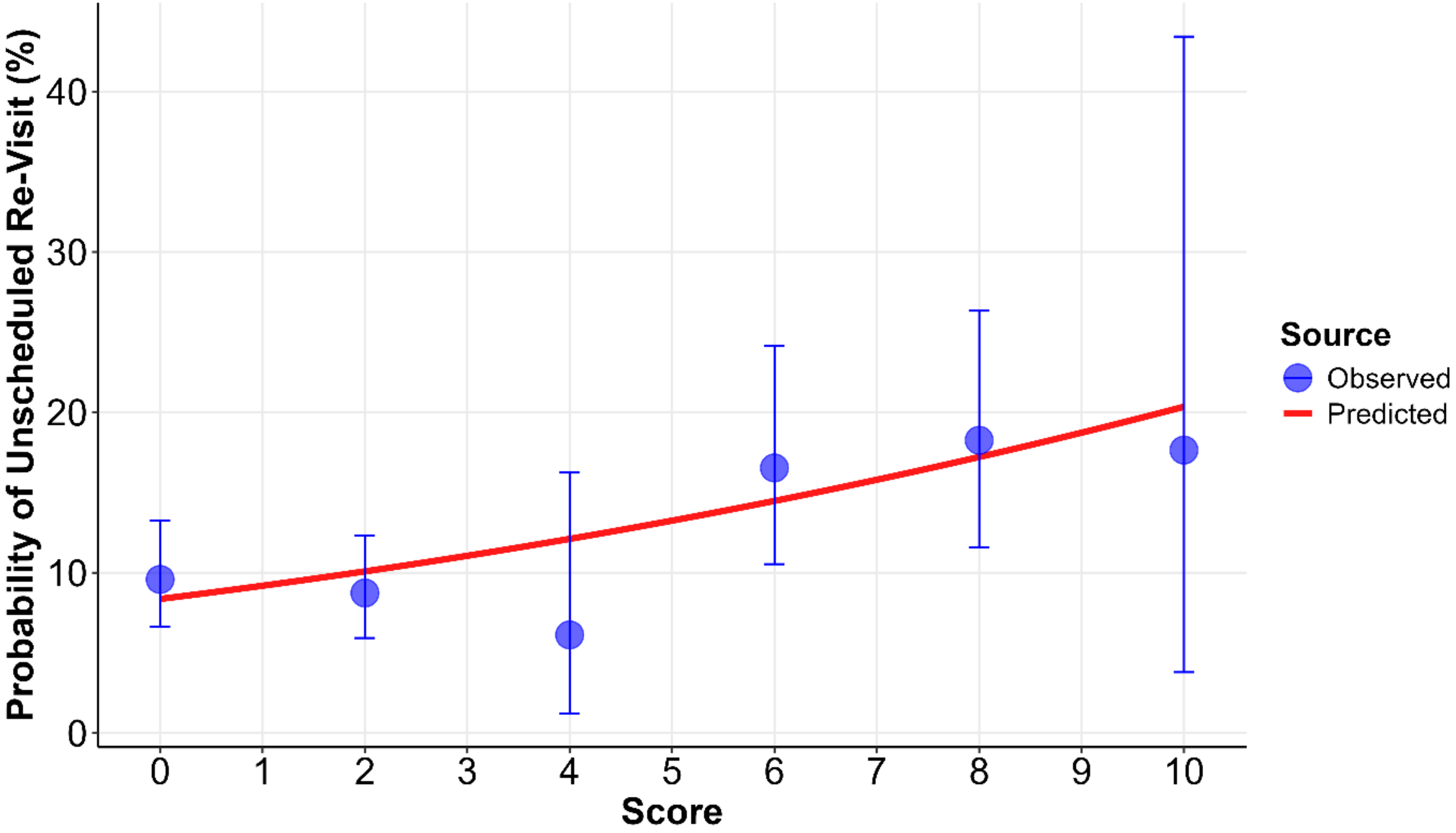

© 2026 Sumner M et al. *JAMA Network Open.*

eFigure 3. Receiver Operating Characteristic Curve of the Derived Score to Correctly Classify the Occurrence of (A) Unscheduled Healthcare Revisit, (B) Intravenous Fluid Administration, and (C) Hospitalization Within 7-Days of Index ED Discharge

A. Unscheduled healthcare revisit

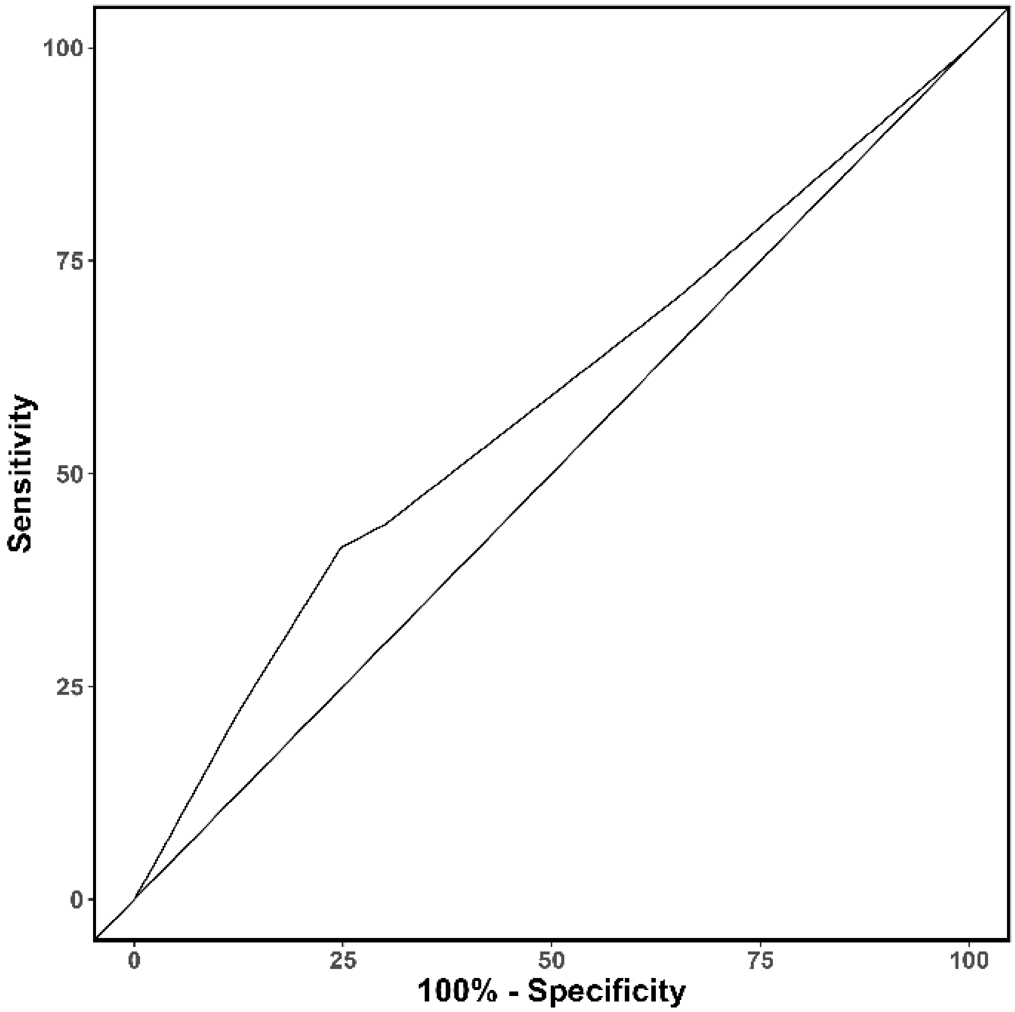

- Area under the curve (AUC) of score for classifying an unscheduled health care visit within 7 days of ED discharge: AUC = 0.57 (95%CI: 0.52, 0.63)
- Mean AUC from 1000 bootstrap samples: 0.57 (95%CI: 0.52, 0.63)
- Standard deviation of AUC from 1000 bootstrap samples: 0.03
- Standard error of AUC from 1000 bootstrap samples: 0.001

© 2026 Sumner M et al. *JAMA Network Open.*

B. Intravenous fluid administration

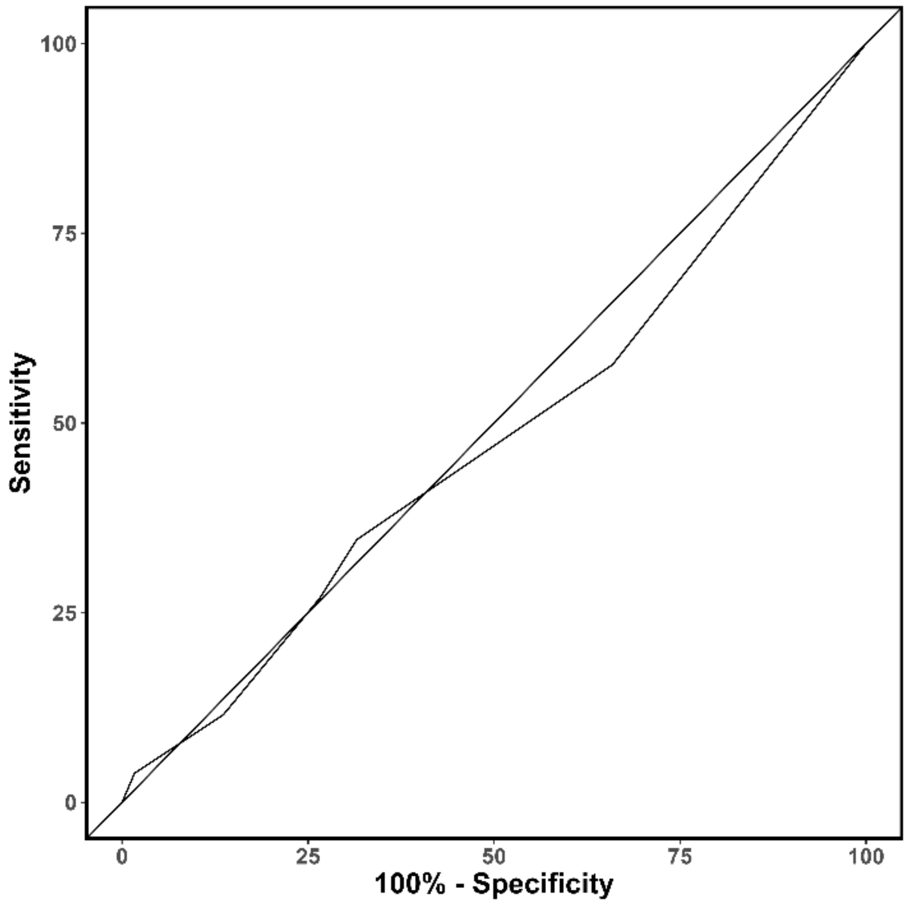

- AUC of revisit-score for classifying receiving intravenous fluid within 7 days of ED discharge: AUC = 0.48 (95%CI: 0.36, 0.59)
- Mean AUC from 1000 bootstrap samples: 0.52 (95%CI: 0.43, 0.64)
- Standard deviation of AUC from 1000 bootstrap samples: 0.06
- Standard error of AUC from 1000 bootstrap samples: 0.002

© 2026 Sumner M et al. *JAMA Network Open.*

C. Hospitalization

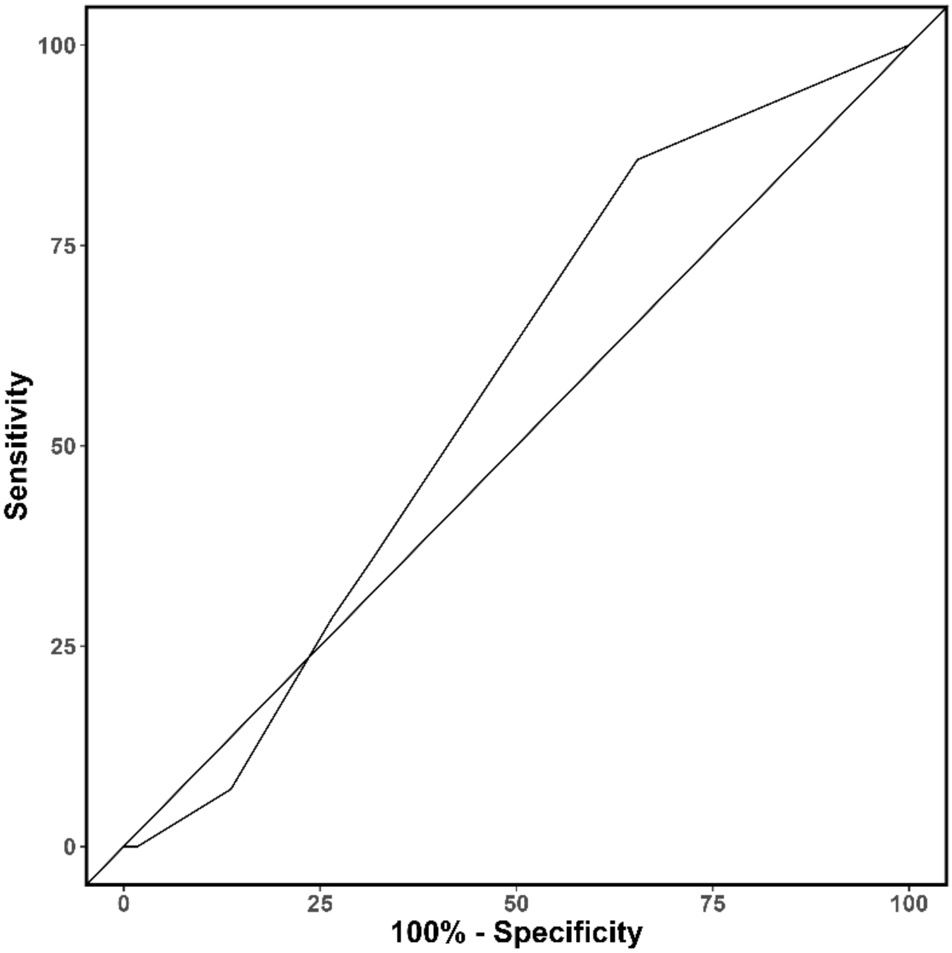

- AUC of score for classifying hospitalization within 7 days of ED discharge: AUC = 0.57, 95%CI: 0.45, 0.69
- Mean AUC from 1000 bootstrap samples: 0.57 (95%CI: 0.45, 0.69)
- Standard deviation of AUC from 1000 bootstrap samples: 0.06
- Standard error of AUC from 1000 bootstrap samples: 0.002

© 2026 Sumner M et al. *JAMA Network Open.*

eTable 1. Results of Mixed-Effect Logistic Regression Model for Each Outcome, With the Derived Score as the Independent Variable

Outcome	Odds ratio (95%CI)	P value
≥3 episodes of vomiting within 24 hours	1.16 (1.08, 1.25)	<0.001
Unscheduled healthcare revisit within 7-days	1.11 (1.04, 1.18)	<0.001
Intravenous fluid administration within 7-days	0.99 (0.86, 1.13)	0.87
Hospitalization within 7-days	1.04 (0.88, 1.24)	0.64

© 2026 Sumner M et al. *JAMA Network Open.*

eTable 2. Effect Estimates for Occurrence of ≥3 Episodes of Vomiting in the 24-Hours Following Emergency Department Discharge Evaluated Using Complete Case and Multiple Imputation Datasets Analyzed Using Mixed-Effects Logistic Regression, Linear Probability Models With Robust Standard Errors, and LASSO-Selected Models

Variables	Complete case data				Multiple imputation data			
	**GLMM, full model Adjusted OR (95%CI)**	**GLMM, reduced model Adjusted OR (95%CI)**	**OLS with Robust SE, full model Estimate (95%CI)**	**GVIF**	**LASSO Penalized log-odds coefficient**	**GLMM, full model Adjusted OR (95%CI)**	**GLMM, reduced model Adjusted OR (95%CI)**	**FMI**
**Sex**				1.02				
Female	0.82 (0.51, 1.31)	NA	-0.02 (-0.05, 0.02)		NA	0.83 (0.51, 1.34)	NA	0.11
Male	reference	NA	reference		NA	reference	NA	
**Age**				1.06				
6 months – < 2 years	2.05 (0.81, 5.24)	2.05 (0.81, 5.15)	0.07 (-0.01, 0.14)		0.630	1.68 (0.72, 3.94)	1.66 (0.72, 3.83)	0.03
2 years – < 5 years	0.87 (0.33, 2.29)	0.86 (0.33, 2.25)	-0.01 (-0.07, 0.06)		NA	0.75 (0.31, 1.84)	0.73 (0.3, 1.77)	0.06
5 years – < 10 years	0.90 (0.34, 2.40)	0.88 (0.33, 2.33)	-0.01 (-0.07, 0.06)		-0.08	0.75 (0.30, 1.85)	0.72 (0.3, 1.77)	0.04
≥ 10 years	reference	reference	reference		NA	reference	reference	
**Baseline duration of symptoms**				1.14				
< 24 hours	reference	reference	reference		NA	reference	reference	
24 hours – < 48 hours	1.44 (0.81, 2.60)	1.39 (0.79, 2.44)	0.03 (-0.02, 0.08)		0.16	1.51 (0.84, 2.7)	1.48 (0.85, 2.6)	0.12
48 hours – 72 hours	1.25 (0.57, 2.76)	1.20 (0.56, 2.58)	0.02 (-0.05, 0.08)		NA	1.22 (0.54, 2.74)	1.22 (0.56, 2.66)	0.11
**Baseline vomiting episodes in 24 hours prior to the ED visit**				1.08				
3 – < 5 episodes	reference	reference	reference		NA	reference	reference	
5 – < 10 episodes	1.14 (0.56, 2.34)	1.17 (0.58, 2.38)	0.01 (-0.04, 0.06)		NA	1.17 (0.57, 2.41)	1.21 (0.59, 2.47)	0.05
≥ 10 episodes	1.57 (0.77, 3.20)	1.62 (0.81, 3.25)	0.04 (-0.02, 0.09)		0.15	1.71 (0.84, 3.48)	1.77 (0.88, 3.55)	0.06
**Baseline diarrhea episodes in 24 hours prior to the ED visit,**				1.06				
≥ 5 episodes	0.90 (0.42, 1.94)	NA	-0.01 (-0.07, 0.06)		NA	1.09 (0.52, 2.28)	NA	0.15
< 5 episodes	reference	NA	reference		NA	reference	NA	
**Fever at baseline**				1.10				
Yes	0.85 (0.50, 1.42)	NA	-0.01 (-0.05, 0.03)		NA	0.85 (0.51, 1.43)	NA	0.07

© 2026 Sumner M et al. *JAMA Network Open.*

No	reference	NA	reference		NA	reference	NA	
**Intravenous fluids during index ED visit**				1.03				
Yes	1.05 (0.42, 2.62)	NA	0.01 (-0.07, 0.08)		NA	1.06 (0.43, 2.64)	NA	0.12
No	reference	NA	reference		NA		NA	
**Ondansetron administered within 24 hours of ED discharge**				1.01				
Yes	2.65 (1.61, 4.38)	2.65 (1.61, 4.36)	0.09 (0.04, 0.15)		0.901	2.38 (1.46, 3.9)	2.39 (1.46, 3.91)	0.03
No	reference	reference	reference			reference	reference	

GLMM: Generalized Linear Mixed-Effects Model – logistic regression with random intercept for site
OLS: Ordinary Least Squares – linear probability model
Robust SE: Eicker–Huber–White heteroskedasticity-robust standard errors
GVIF: Generalized Variance Inflation Factor
LASSO: Least Absolute Shrinkage and Selection Operator
FMI: Fraction of Information Missing
OR: Odds Ratio
CI: Confidence Interval
ED: Emergency Department
The full model includes all candidate predictors selected a priori based on clinical relevance.
The reduced model includes predictors retained following LASSO variable selection.
GVIF values are reported to assess multicollinearity.

FMI values are reported for each model parameter to quantify the impact of missing data on estimation precision. The maximum FMI observed was 0.15, corresponding to a relative efficiency of approximately 98.5% with 10 imputations. This indicates minimal loss of statistical information due to missing data and suggests that multiple imputation estimates closely approximate those that would be obtained with fully observed data.

© 2026 Sumner M et al. *JAMA Network Open.*

eReferences.

1. Schelldorfer J, Meier L, Bühlmann P. GLMMLasso: An Algorithm for High-Dimensional Generalized Linear Mixed Models Using ℓ1-Penalization. *Journal of Computational and Graphical Statistics.* 2014;23(2):460-477.
2. Zahid FM, Faisal S, Heumann C. Variable selection techniques after multiple imputation in high-dimensional data. *Statistical Methods & Applications.* 2020;29(3):553-580.
3. Austin PC, Lee DS, D'Agostino RB, Fine JP. Developing points-based risk-scoring systems in the presence of competing risks. *Stat Med.* 2016;35(22):4056-4072.

© 2026 Sumner M et al. *JAMA Network Open.*
