# Supplementary material for: Persistent Vomiting Among Children With Acute Gastroenteritis: A Secondary Analysis of a Randomized Clinical Trial
Source: JAMA Netw Open. 2026 May 6;9(5):e2610898. doi: 10.1001/jamanetworkopen.2026.10898 (PMC13150644; doi:10.1001/jamanetworkopen.2026.10898)
Supplement: Supplement 3. — Members of Pediatric Emergency Research Canada [file jamanetwopen-e2610898-s003.pdf]

Supplemental Online Content: Nonauthor Collaborators
*First name, last name, and suffix (if applicable) are required and will appear in PubMed.

*Group Name(s): Pediatric Emergency Research Canada							
***First Name and Middle Initial(s)**	***Last Name**	***Suffix (eg, Jr, III)**	Academic Degrees	Institution	Location (city, state/province, country)	Role or Contribution, eg, chair, principal investigator	Group (if more than 1 Group listed in the byline) and/or Subgroup (eg, Steering Committee)
Samina	Ali		MDCM	University of Alberta	Edmonton, AB, Canada	Chair	
Roger	Zemek		MD	University of Ottawa	Ottawa, ON, Canada	Past-Chair	
Naveen	Poonai		MD, Msc	University of Western Ontario	London, ON, Canada	Member	
Bruce	Wright		MD	University of Alberta	Edmonton, AB, Canada	Member	
Gabrielle	Freire		MDCM, MH	University of Toronto	Toronto, ON, Canada	Member	
Vikram	Sabhaney		MD	University of British Columbia	Vancouver, BC, Canada	Member	
Jocelyn	Gravel		MD, Msc	Universite de Montreal	Montreal, QC, Canada	Member	
Tyrus	Crawford		BSc	University of Ottawa	Ottawa, ON, Canada	Member	
Henry	Li		MD	University of Alberta	Edmonton, AB, Canada	Member	
Andrea	Eaton		RN, MSc	University of Alberta	Edmonton, AB, Canada	Network Coordinator	
