# Supplementary material for: Persistent Vomiting Among Children With Acute Gastroenteritis: A Secondary Analysis of a Randomized Clinical Trial
Source: JAMA Netw Open. 2026 May 6;9(5):e2610898. doi: 10.1001/jamanetworkopen.2026.10898 (PMC13150644; doi:10.1001/jamanetworkopen.2026.10898)
Supplement: Supplement 4. — Data Sharing Statement [file jamanetwopen-e2610898-s004.pdf]

Data Sharing Statement

Sumner. Persistent Vomiting Among Children with Acute Gastroenteritis. *JAMA Netw Open*. Published May 06, 2026. doi:10.1001/jamanetworkopen.2026.10898

Data
Additional Information: ClinicalTrials.gov number: NCT03851835
Data available: Yes
Data types: Deidentified participant data, Data dictionary
How to access data: Data will be shared, upon reasonable request, for academic purposes, with appropriate individuals who have obtained appropriate ethics permissions and data sharing agreements.
When available: With publication

Supporting Documents
Document types: Statistical/analytic code, Informed consent form
How to access documents: Data will be shared, upon reasonable request, for academic purposes, with appropriate individuals who have obtained appropriate ethics permissions and data sharing agreements.
When available: With publication

Additional Information
Who can access the data: Data will be shared, upon reasonable request, for academic purposes, with appropriate individuals who have obtained appropriate ethics permissions and data sharing agreements.
Types of analyses: Data will be shared, upon reasonable request, for academic purposes, with appropriate individuals who have obtained appropriate ethics permissions and data sharing agreements.
Mechanisms of data availability: Data will be shared, upon reasonable request, for academic purposes, with appropriate individuals who have obtained appropriate ethics permissions and data sharing agreements.
